# Naphthalene Diimide‐Based Metallacage as an Artificial Ion Channel for Chloride Ion Transport

**DOI:** 10.1002/advs.202308181

**Published:** 2024-03-08

**Authors:** Qing‐Hui Ling, Yuanyuan Fu, Zhen‐Chen Lou, Bangkun Yue, Chenxing Guo, Xinyu Hu, Weiqiang Lu, Lianrui Hu, Wei Wang, Min Zhang, Hai‐Bo Yang, Lin Xu

**Affiliations:** ^1^ State Key Laboratory of Molecular & Process Engineering Shanghai Key Laboratory of Green Chemistry and Chemical Processes Shanghai Frontiers Science Center of Molecule Intelligent Syntheses School of Chemistry and Molecular Engineering East China Normal University Shanghai 200062 China; ^2^ Shanghai Key Laboratory of Regulatory Biology Institute of Biomedical Sciences and School of Life Sciences East China Normal University Shanghai 200062 China; ^3^ Key Laboratory of Micro‐Nano Optoelectronic Devices (Wenzhou) College of Electrical and Electronic Engineering Wenzhou University Wenzhou 325035 China; ^4^ College of Chemistry and Environmental Engineering Shenzhen University Guangdong 518055 China

**Keywords:** coordination‐driven self‐assembly, ion channel, metallacage, naphthalene diimide, supramolecular chemistry

## Abstract

Developing synthetic molecular devices for controlling ion transmembrane transport is a promising research field in supramolecular chemistry. These artificial ion channels provide models to study ion channel diseases and have huge potential for therapeutic applications. Compared with self‐assembled ion channels constructed by intermolecular weak interactions between smaller molecules or cyclic compounds, metallacage‐based ion channels have well‐defined structures and can exist as single components in the phospholipid bilayer. A naphthalene diimide‐based artificial chloride ion channel is constructed through efficient subcomponent self‐assembly and its selective ion transport activity in large unilamellar vesicles and the planar lipid bilayer membrane by fluorescence and ion‐current measurements is investigated. Molecular dynamics simulations and density functional theory calculations show that the metallacage spans the entire phospholipid bilayer as an unimolecular ion transport channel. This channel transports chloride ions across the cell membrane, which disturbs the ion balance of cancer cells and inhibits the growth of cancer cells at low concentrations.

## Graphical abstract

A series of metallacages with the activity of ion transmembrane transport are prepared through subcomponent self‐assembly. These metallacages can achieve selective anion transport in living cells and inhibit the growth of cancer cells.

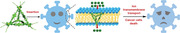

## 1 Introduction

The cell membrane is a semipermeable membrane composed of a phospholipid bilayer. It selectively absorbs nutrients, discharges metabolic waste, and maintains the cell's balance of osmotic pressure.^[^ ^1^ ^]^ Water and carbon dioxide molecules diffuse relatively easily across the phospholipid bilayer, but the transmembrane exchange of most physiologically important anions and cations depends on the help of ion channels or transmembrane carrier proteins.^[^ ^2^ ^]^ If the function of these proteins is impaired, the balance of substances inside and outside the cell is disrupted, causing serious illness. For example, chloride is the most abundant anion in the human body ^[^ ^3^ ^]^; chloride ion channels are widely distributed in the plasma membrane of excitatory cells, non‐excitatory cell membranes, lysosomes, mitochondria, endoplasmic reticulum, and other organoids of the body.^[^ ^4^ ^]^ Chloride ion channel dysfunction can cause diseases in many tissues, including congenital myotonia, recessive generalized myotonia, cystic fibrosis, and hereditary renal lithiasis.^[^ ^5^ ^]^ The mechanism of most ion channel diseases is still unknown, however, and the complex structure and unpredictability of natural ion channels have hindered their applications in disease treatment and other fields.^[^ ^6^ ^]^ Therefore, the development of well‐structured artificial ion channels can provide a theoretical model for the study of ion channel diseases.^[^ ^7^ ^]^ Moreover, artificial ion channels can disrupt the homeostasis of pathogenic cells, offering a promising avenue for antimicrobial and cancer treatment potentially overcoming the problem of drug resistance in traditional cancer therapy.^[^ ^8^ ^]^ The first artificial ion channel, based on cyclodextrin, was reported in 1982.^[^ ^9^ ^]^ Since then, many artificial ion channels have emerged ^[^ ^10^ ^]^; they were constructed from supramolecular structures such as macrocyclic stacked channels,^[^ ^11^ ^]^ nanotube‐based artificial channels,^[^ ^12^ ^]^ and supramolecular cage‐based ion channels.^[^ ^13^ ^]^

Metallacages are 3D structures constructed by coordination between metal centers and organic ligands.^[^ ^14^ ^]^ Due to their unique 3D cavity, which can take a variety of shapes, and efficient self‐assembly, metallacages have a wide range of applications in cargo transport, mixture separation, catalysis, and chemical sensing.^[^ ^15^ ^]^ They also play important roles in biology,^[^ ^16^ ^]^ such as drug and biomolecule delivery, cell imaging, and cancer therapy. In recent years, metallacages have gained interest as unimolecular artificial ion channels. Compared with self‐assembled channels, unimolecular channels are large enough to insert into phospholipid membranes and remain intact inside the phospholipid bilayer.^[^ ^6a^ ^]^ For example, the stable metallacage MOP‐18 was used to construct an artificial ion channel that transported protons and alkali metal ions across the phospholipid bilayer.^[^ ^13b^ ^]^ In addition, a metallacage with the geometry of a cuboctahedron was found to generate two conductance states in the phospholipid bilayer.^[^ ^13c^ ^]^ A pentagonal prismatic metallacage was used to construct a gated artificial ion channel, which utilized the difference in host‐guest interaction strengths between the metallacage and guest molecules to achieve gated ion transport.^[^ ^13d^ ^]^ Furthermore, metallacage‐based artificial ion channels have some potential advantages in cancer treatment. For example, the shape, size, and chemical environment of the cavities and windows of the metallacages were easily tuned by the replacement of metal centers and simple modification of the organic ligands, which is beneficial to improving the selectivity and adaptability of ion channels. Moreover, metal centers in metallacages with imaging, diagnosis, and therapy properties could assist metallacage‐based artificial ion channels to achieve multi‐modal cancer diagnosis and treatment.^[^ ^17^ ^]^

**Figure 1:**
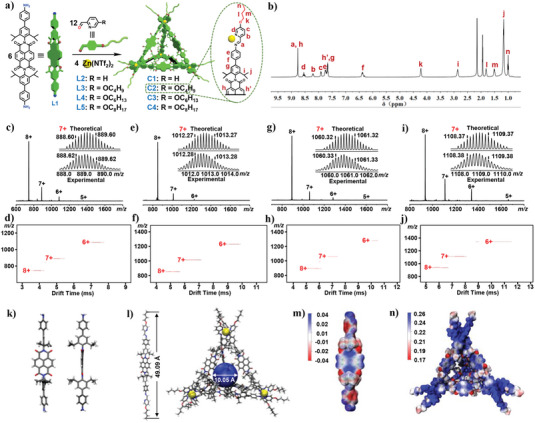
a) Subcomponent self‐assembly of tetrahedra **C1**‐**C4** and b) ^1^HNMR spectrum of **C2** (CD_3_CN, 500 MHz, 298 K). Mass spectrometric characterization of metallacages **C1**‐**C4**. ESI‐MS spectra of c) **C1**, e) **C2**, g) **C3**, and i) **C4**. TWIM‐MS plots (m/z vs drift time) of d) **C1**, f) **C2**, h) **C3**, and j) **C4**. k) Front view (left) and side view (right) of single‐crystal structure of ligand **L1**. l) Simulated geometric structures of flat side (right) and edge (left) of metallacage **C2** (right). m,n) DFT‐computed electrostatic potential maps for ligand **L1** (m) and metallacage **C2** (n).

**Scheme 1:**
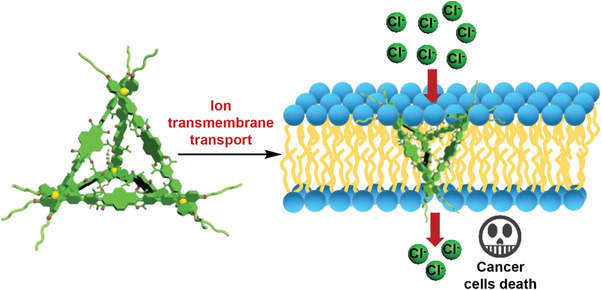
Schematic diagram for the unimolecular ion channel formed by metallacage **C2** for chloride ion transport.

Several criteria are important in the design of artificial ion channels based on metallacages.^[^ ^6^, ^18^ ^]^ First, the channel needs to be lipophilic to ensure insertion into the phospholipid bilayer. Second, the channel must be long enough to span the entire phospholipid bilayer thickness, ≈3.5 nm. Third, because ion selectivity is critical to the performance of artificial ion channels, there must be ion recognition sites on the channel. So far, only a few examples of metallacages as artificial ion channels have been reported. In particular, no metallacage‐based artificial ion channels have yet been reported for ion transmembrane transport in living cells. We designed a series, **C1**–**C4**, of metallacages based on naphthalene diimide (NDI), as anion channels, with the aim of transporting chloride ions in cancer cells and so disturbing their ion balance (**Figure** **1**a). Alkyl chains were introduced at the apex of the metallacages to facilitate their embedding into the phospholipid bilayer. We selected zinc(II) bis(trifluoromethane)sulfonimide (Zn(NTf_2_)_2_) as the coordination node because Zn is an essential trace element in the human body and thus has good biocompatibility.^[^ ^19^ ^]^ Therefore, the NDI‐based subcomponent **L1** was rigid to ensure that **C1**‐**C4** have fixed cavity structures in the phospholipid bilayer. In addition, NDI surfaces have high π acidity and have anion‐π interactions with halogen ions,^[^ ^20^ ^]^ introducing ion recognition sites into the metallacages. We found that **C2** with appropriate alkyl chain length transported chloride ions across phospholipid membrane with the best activity. Furthermore, **C2** inhibited the activity of cancer cells at low concentrations by disrupting the chloride balance of cancer cells, which might provide a new type of cancer treatment (**Scheme** **1**). Among these reported metallacages with the activity of ion transmembrane transport, this is the first case of metallacage‐based artificial ion channels achieving inhibitory activity of cancer cells, to the best of our knowledge.

## 2 Results and Discussion

The self‐assembly of NDI‐based metallacages **C1**–**C4** is described in Figure 1a. First, subcomponent **L1** was prepared by Suzuki coupling 4‐aminophenylboronic acid pinacol ester and compound **2**, with moderate yield (Scheme S2, Supporting Information). Single crystal X‐ray crystallography of **L1** revealed that it was a linear rigid ligand with the NDI plane perpendicular to the benzene ring at both longitudinal ends due to the steric hindrance from the diisopropyl group (Figure 1k). Since the hydrophobic alkyl chain has a great influence on the insertion of the metallacage into the phospholipid bilayer, subcomponents **L2** (without alkyl chains) and **L3**–**L5** (with alkyl chains of different lengths) were chosen to prepare metallacages **C1**–**C4**. As shown in Schemes S6–S9 (Supporting Information), the tetrahedral Zn^II^_4_L_6_ metallacages **C1**‐**C4** were obtained by the self‐assembly of subcomponents **L2**–**L5** (12 equiv), NDI‐based diamine **L1** (6 equiv), and Zn(NTf_2_)_2_ (4 equiv) in acetonitrile for 12 h at 65 °C, respectively. Multinuclear NMR (^1^H, ^13^C, 2D diffusion ordered spectroscopy (2D DOSY) and ^1^H‐^1^H correlation spectroscopy (^1^H‐^1^H COSY)) analysis of assemblies **C1**‐**C4** revealed the formation of discrete metallacages. For instance, in the ^1^H NMR spectra of the assemblies **C1**‐**C4**, the characteristic aldehyde peak at 9.90 ppm disappeared and was replaced by a peak at ≈8.77 ppm characteristic of imidyl hydrogen. This demonstrated successful aldehyde‐amine condensation (Figures S26,  S31,  S36, and S41, Supporting Information). Since the NDI plane was perpendicular to the benzene ring at both vertical ends, the hydrogen h’ (Figure 1b) on the NDI was inside the metallacage cavity after assembly. Compared with hydrogen h outside of the cavity, chemical shift of hydrogen h' was shifted upfield by ≈1.0 ppm due to shielding by the metallacage cavity.^[^ ^21^ ^]^ In addition, the rotation of benzene ring close to the coordination site was blocked, splitting the peak of hydrogen f into two. Variable temperature ^1^H NMR spectra showed that the signal of hydrogen f gradually changed into a set of peaks as the temperature increased (Figure S1, Supporting Information).

Electrospray ionization mass spectrometry (ESI‐MS) was also employed to confirm the formation of the discrete metal‐lacages. For example, as shown in Figure 1e, the ESI‐MS spectrum of metallacage **C2** revealed one dominant set of peaks with continuous charge states ranging from 6+ to 8+ due to the successive loss of counterions, that is, NTf_2_^−^. Experimental isotope patterns agreed well with simulated isotope patterns (Figure S49, Supporting Information). Similar ESI‐MS spectra were obtained in the case of metallacages **C1**, **C3**, and **C4** (Figure 1c,g,i), and undesired fragmentation was negligible in the ESI‐MS spectra of **C1**–**C4**. After deconvolution, the average molar masses of metallacages **C1**–**C4** were determined to be 8183.3, 9048.2, 9385.5, and 9721.8 Da, respectively, matching well with their expected chemical compositions, that is, [**L1**_6_ **L2**_12_Zn_4_(NTf_2_)_8_] (**C1**), [**L1**_6_ **L3**_12_Zn_4_(NTf_2_)_8_] (**C2**), [**L1**_6_ **L4**_12_Zn_4_(NTf_2_)_8_] (**C3**), [**L1**_6_ **L5**_12_Zn_4_(NTf_2_)_8_] (**C4**). Furthermore, traveling wave ion mobility mass spectrometry (TWIM‐MS)^[^ ^22^ ^]^ was applied to confirm that tetrahedral metallacages were the predominant ensembles for **C1**–**C4** (Figure 1d,f,h,j). Each spectrum showed a series of narrowly distributed bands for each charge state, excluding the formation of discernible structural isomers or conformers.

To unambiguously confirm the structures of these tetrahedral metallacages, we attempted to obtain single crystals of **C1**–**C4**. Unfortunately, all attempts to obtain X‐ray‐quality single crystals of metallacages **C1**–**C4** proved unsuccessful because of the low stability of single crystals of the metallacages. In order to improve the stability of a single crystal,^[^ ^23^ ^]^ [**L1**_6_ **L3**_12_Fe_4_(NTf_2_)_8_] (**C2’**) was prepared. Although a stable purple quadrate single crystal of **C2’** was obtained, its X‐ray diffraction result was far away from the requirements of data acquisition (Figure S55, Supporting Information). Thus, we obtained a MOPAC optimized structure of **C2** with the PM6 semiempirical method. As shown in Figure 1l, the length of an edge of the simulated **C2** model was ≈4.9 nm, long enough to insert in the phospholipid bilayer as an unimolecular channel. In addition, the optimized model showed that the hydrogens on one side of the NDI core face into the metallacage cavity, consistent with the shielding effect in the ^1^H NMR spectrum. The electrostatic potential map of **C2** computed by density functional theory (DFT) showed a large electrostatic potential on the metal ions and NDI core (Figure 1m,n), suggesting that **C2** was strongly electrophilic and has the potential to transport anions.

**Figure 2:**
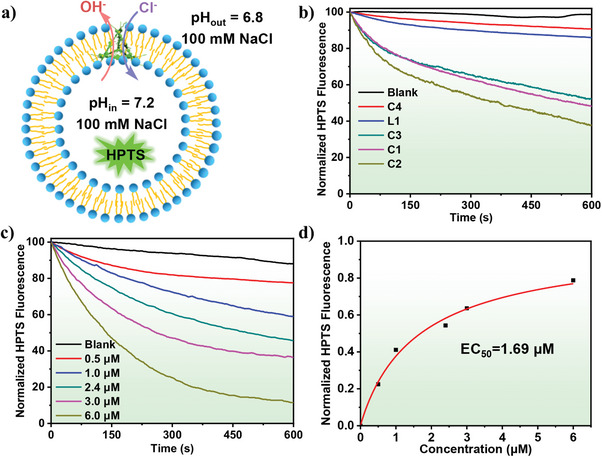
a) Schematic illustration of the pH‐sensitive LUVs⊃HPTS assay. b) Ion transport activities of control compounds **L1** and **C1**–**C4** at 1.0 mm. c) Concentration‐dependent enhancement of ion transport activity of **C2** (0–6.0 µm) across LUVs⊃HPTS; d) The concentration‐activity curve. Red lines are fitted curves from the Hill equation.

Fluorometric analyses were carried out to determine the ion transport activity of **C1**–**C4** and **L1** across large unilamellar vesicles (LUVs). As shown in **Figure** **2**a, 8‐hydroxypyrene‐1,3,6‐trisulfonate (HPTS)^[^ ^3d^ ^]^ was used as a pH‐sensitive fluorescence probe to evaluate the channeling activities of NDI metallacages. HPTS‐encapsulated LUVs were prepared as previously reported from 1,2‐diacyl‐sn‐glycero‐3‐phosphocholine, 3‐hydroxy‐5‐cholestene, and HPTS.^[^ ^24^ ^]^ The aqueous interior of LUVs was buffered at pH 7.2 (100 mm NaCl) and the exterior was buffered at pH 6.8 (100 mm NaCl) to generate a pH gradient. The change in the fluorescence intensity was monitored after the addition of **C1**‐**C4** and **L1**, separately. In the presence of a NaCl exterior buffer solution (*M* = Na+ and *X* = Cl‐), a pronounced reduction in HPTS fluorescence emission was observed upon the addition of 0.9 mol% of metallacage **C2**. This decrease amounted to 63% compared to the blank control group, as illustrated in Figure 2b. This decrease in HPTS fluorescence suggested proton influx or OH^−^ efflux in response to the transmembrane pH gradient of 0.4. The metallacages’ ionophoric activities were strongly influenced by the peripheral hydrophobic alkyl chain substituents. Higher activity was observed with metallacages containing flexible hydrophobic alkyl chains of moderate length (*R* = ‐OC_4_H_9_). Metallacage **C1** without alkyl chains displayed impaired channel activity (52%) because the metallacage was difficult to embed into the phospholipid bilayer without the help of hydrophobic alkyl chains. The longer alkyl chain‐substituted metallacage **C3** (*R* = ‐OC_6_H_13_) with the same loading (0.9 mol%) showed a moderate decrease in HPTS fluorescence emission (≈51%). Metallacage **C4**, which is substituted with extended alkyl chains (*R* = −OC8H17), exhibited minimal channel activity. The fluorescence emission in the presence of **C4** only decreased by 10% compared to the blank control group. The reduced activity of **C3** and negligible activity of **C4** might be due to excessive conformational distortion from the flexible chain closing the channel.^[^ ^6^, ^13^, ^15^ ^]^ To investigate the critical role of the 3D cavity of metallacage as a unimolecular ion channel, the subcomponent **L1** was used as a control. As shown in Figure 2b, **L1** was inactive in ion transport, indicating that the 3D metallacages played a vital role in ion transport.

In order to confirm that the fluorescence intensity increase was associated with the presence of metallacage **C2**, a blank experiment in the absence of **C2** under otherwise identical conditions was performed. Only a very small increase (≤3%) of fluorescence intensity was observed in the blank experiment even after 600 s, supporting the hypothesis that metallacage **C2** was responsible for the observed increase in fluorescence intensity. At a concentration of 1.8 mol% (6.0 µm), metallacage **C2** reaches 88% maximal fluorescence intensity without pre‐incubation of the metallacage with LUVs (Figure 2c). The corresponding EC_50_ value (the concentration required to reach 50% transmembrane performance) was determined via the Hill equation to be 1.69 µm (Figure 2d). These results indicated that **C2** with alkyl chains of appropriate length has good activity as a unimolecular artificial ion channel.

**Figure 3:**
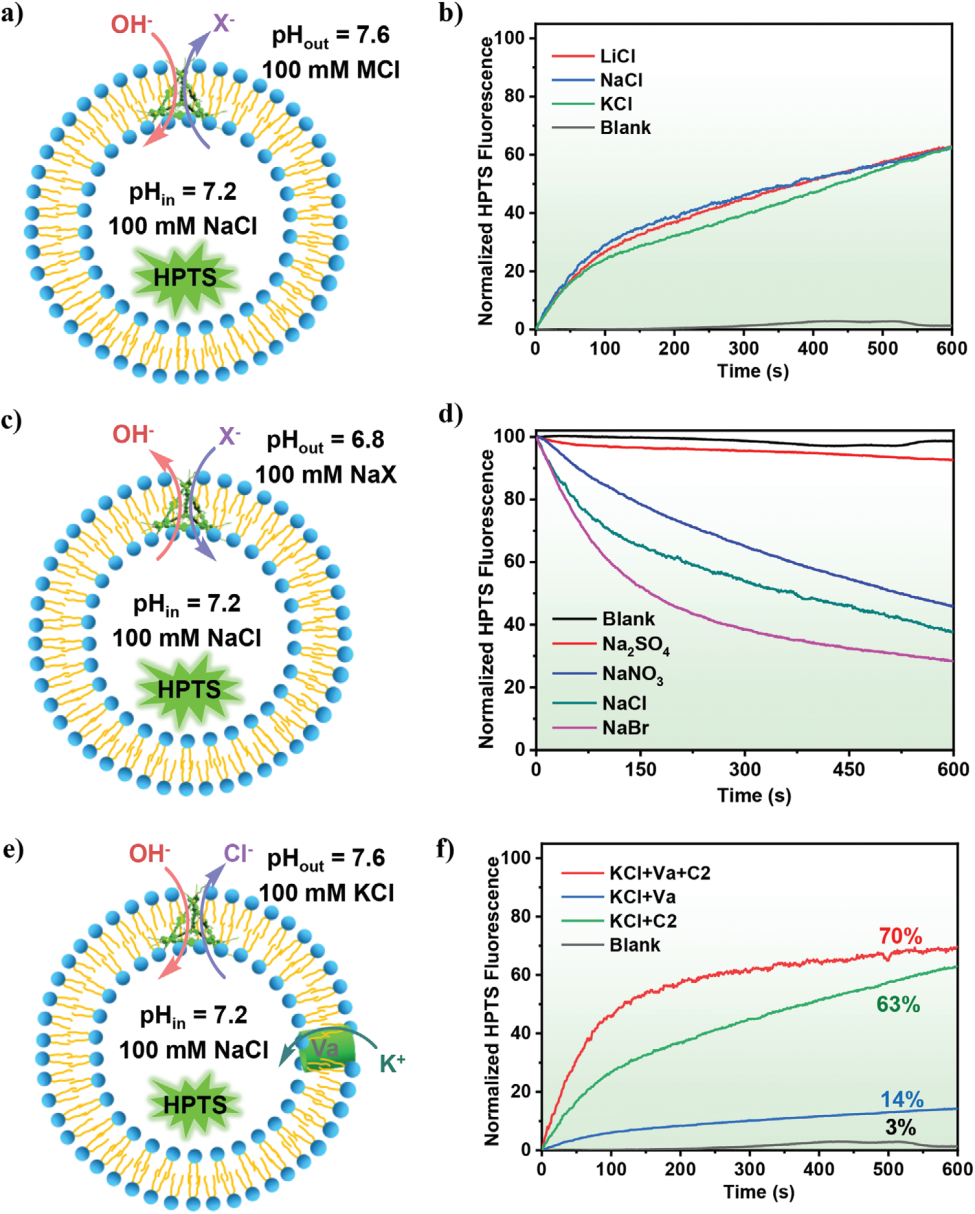
a), c), and e) Schematic illustration of the pH‐sensitive LUVs⊃HPTS assay. b) Transport selectivity of alkali metal ions by **C2** (3.0 µm) obtained from the HPTS assay by varying the extravesicular MCl (M = Li^+^, Na^+^, or K^+^). d) Transport selectivity of anions by **C2** (3.0 µm) obtained from the HPTS assay by varying the extravesicular NaX (*X* ^−^ = Cl^−^, Br^−^, NO_3_^−^, or SO_4_^2−^). f) Ion transport activity of **C2** in the absence or presence of a potassium carrier, valinomycin (Va), indicating a preferential transport of Cl^−^ over OH^−^.

The pronounced ion transport activity of **C2** prompted us to investigate its ion selectivity. An almost unchanged decrease in HPTS fluorescence emission was observed when the extravesicular cation was changed from Na^+^ to Li^+^ or K^+^ (**Figure** **3**a,b), which suggested that the cations were not involved in the ion transport process. Since the HPTS assay was insensitive to external cation change, metallacage **C2** was unlikely to mediate cation transport. We then performed HPTS assays in the presence of different internal and external anions, using NaX as the salt in the buffer solution (*X^−^* = Cl^−^, Br^−^, NO_3_^−^, or SO_4_^2−^) (Figure 3c). The HPTS fluorescence intensity decreased gradually after the addition of 3.0 µm **C2**. The rate of OH^−^ efflux or H^+^ influx from LUVs correlated with the anion influx rate, which was computed from the decrease rate of HPTS fluorescence intensity. When the cations (*M^+^* = Na^+^) remained the same, as shown in Figure 3d, obvious differences in the fluorescence intensity decrease were observed when different anions were used. A much faster decrease in HPTS fluorescence emission was observed when halides (Cl^−^, Br^−^) and NO_3_^−^ were used. The anion transport activity of metallacage **C2** decreased in the order of Br^−^>Cl^−^>NO_3_^−^>SO_4_^2−^. The anion transport capability of **C2** was likely due to the electrostatic interaction with the metal cation center with potential minor contributions from anion‐π interactions between **C2** and anions.^[^ ^10c,d,f^ ^]^

OH^−^ is involved in the OH^−^/Cl^−^ antiporter by balancing charge. In particular, OH^−^ regulates the pH of body fluids, while Cl^−^ is the most abundant anion in biological systems. We introduced a potassium carrier, valinomycin (Va) ^[^ ^25^ ^]^ with **C2**, to compare the transport rate between OH^−^ and Cl^−^. We observed the decay rate of transmembrane pH difference in the HPTS assay, with KCl as the external salt (Figure 3e). K^+^ transport, mediated by Va, induced anion symport of OH^−^ or Cl^−^ by **C2** to maintain overall charge balance. If OH^−^ was preferentially transported, an increase in fluorescence intensity should be observed, while if Cl^−^ was preferentially transported the increase in fluorescence intensity should remain almost unchanged. Va without **C2** (0.0036 mol% relative to lipid) produced a normalized HPTS fluorescence intensity of 14%, while **C2** without Va (0.9 mol% relative to lipid) reached 63%. The combination of Va and **C2** significantly increased fluorescence intensity to 70%. However, the trend of the ion transport rate curves of Va+**C2** as well as **C2** alone were essentially identical after 150 s. In addition, Va+**C2** had a nearly constant ion transport rate after 600 s (67% = 70%−3%) relative to the sum of the individual components, 71% = (63%–3%)+(14%–3%) from **C2** and Va, respectively (Figure 3f). Therefore, the results indicated preferential transport of Cl^−^ over OH^−^.

In order to investigate whether ion transport was mediated by the metallacage **C2**, the possibility of transporting the large dye calcein was explored. Calcein is a concentration‐sensitive fluorescent dye; transporting it from the intravesicular to the extravesicular solution will lead to an increase in fluorescence.^[^ ^5b^ ^]^ The simulated structure of **C2** has a cavity diameter of ≈1.0 nm, which was not large enough to transport a large dye like calcein (with a hydrated diameter of ≈1.6 nm). We performed assays with calcein‐encapsulated LUVs (40 mm calcein, 100 mm NaCl, pH 7.4; external buffer 100 mm NaCl, pH 7.4). In the absence of **C2**, the fluorescence intensity of calcein increased <10% after 300 s. Adding **C2** yielded an almost negligible increase in the fluorescence intensity (Figure S11, Supporting Information), indicating that **C2** was incapable of transporting calcein across the membrane and maintained the integrity of LUVs.

The interaction between the ion and its recognition site on an artificial ion channel should be neither too weak nor too strong. Initial ^1^H NMR titrations of tetrabutylammonium chloride (TBACl), tetrabutylammonium bromide (TBABr) and tetrabutylammonium nitrate (TBANO_3_) into a solution of **C2** in CD_3_CN suggested that there was a host‐guest interaction between **C2** and the anion X^−^ (*X^−^* = Cl^−^, Br^−^, or NO_3_^−^). The chemical shift peak at ≈8.8 ppm changed obviously (Figures S3–S5, Supporting Information), indicating possible interactions between anions and the electron‐deficient NDI π‐plane or electrostatic interaction between anions and the four vertices with high electrostatic potential (Figure 1n). Subsequently, UV–vis titrations were used to measure the binding between **C2** and the TBAX anions in CH_3_CN. As shown in Figures S6–S8 (Supporting Information), the results obtained from Job plot experiments indicated 1:2 stoichiometry for the host‐guest complexes of **C2** and Cl^−^ or Br^−^ in CH_3_CN and 1:1 stoichiometry of **C2** and NO_3_^−^. In addition, analysis of the data on the basis of a 1:2 and 1:1 noncooperative binding model by using Bindfit^[^ ^26^ ^]^ indicated that the first binding constants of **C2** with Cl^−^ and Br^−^ were K1 = (1.4 ± 0.1) × 10^3^ m ^−1^ and (1.5 ± 0.4) × 10^4^ m ^−1^, respectively, and the binding constant of **C2** with NO_3_^−^ was (2.9 ± 0.1) × 10^4^ m ^−1^. The moderate binding between **C2** and anions might favor ion transport in the phospholipid bilayer.

**Figure 4:**
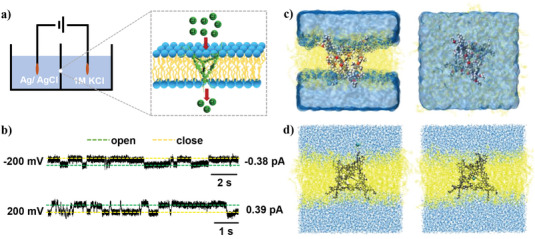
a) Schematic representation of current recording in patch clamp experiments. b) Single‐channel current traces recorded at −200 mV (top) and +200 mV (bottom) holding potentials in 1 m symmetrical KCl solution. c) Front (left) and vertical view (right) of **C2** simulated system embedded in a solvated lipid bilayer computed by diPhyPC (blue area: water, yellow area: lipid bilayer.) d) Snapshots of **C2** with chloride in lipid bilayer, used in umbrella sampling simulation.

To obtain evidence for ion channel formation by **C2**, we measured the electrical conductance across a planar lipid bilayer membrane.^[^ ^27^ ^]^ The planar lipid bilayer, made from 1,2‐diphytanoyl‐sn‐glycero‐3‐phosphocholine (diPhyPC) lipid, was prepared over an orifice connecting two electrolyte chambers (**Figure** **4**a). The electrical conductance between the two chambers was measured for different applied voltages, providing information about the formation of ion channels.^[^ ^8b^ ^]^ As shown in Figure 4b, a significant ion conduction signal was observed after adding **C2** into the *cis* chamber of the electrolyte solution, unambiguously confirming that **C2** mediated chloride transmembrane transport via a channel mechanism. Furthermore, we calculated the Cl^−^ conductance of **C2** to be 1.95 ± 0.034 pS from the fitted linear current–voltage (*I*–*V*) plot. The permeability ratio of K^+^ (P_K_^+^/P_Cl_^−^) was calculated to be 0.28, the relatively low permeability of K^+^ indicated that **C2** has obvious anion selectivity (Figure S12d, Supporting Information). These results suggested that **C2** transported chloride ions efficiently, validating the initial goal of exploring **C2**‐mediated chloride ion transport in living cells.

In addition, to substantiate the reasonableness of the molecular design of **C2**, we simulated **C2** in a diPhyPC lipid bilayer with molecular dynamics (MD) simulations. As shown in Figure 4c, **C2** retained its structure over a 100 ns equilibration. Four vertices with hydrophobic alkyl chains were stably embedded into the planar diPhyPC lipid bilayer, with two edges of the tetrahedral metallacage **C2** parallel to the upper and lower sides of the lipid bilayer. We additionally performed an umbrella sampling simulation to explore the free energy associated with chloride movement through **C2** (Figure 4d). The estimated free energy barriers between the chloride ion and **C2** were low (<10 kcal mol^−1^), suggesting that chloride entered **C2** quickly and reversibly (Figure S14, Supporting Information). These results provided theoretical support for the proposed mechanism of chloride ion transport mediated by **C2**.

**Figure 5:**
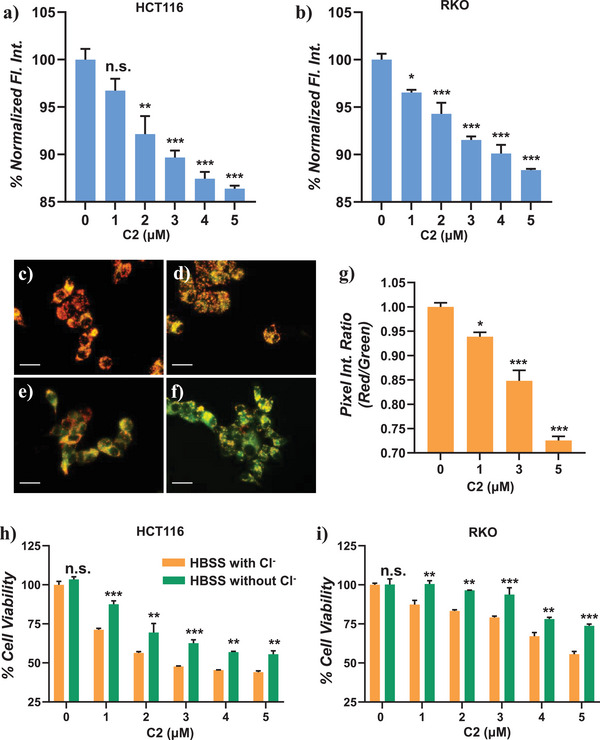
Normalized fluorescence intensity of a) HCT116 cells and b) RKO cells, incubated with MQAE (5 mm) for 3 h followed by treatment of **C2** (0–5 µm) for 24 h. Cell imaging of HCT116 cells incubated with c) 0, d) 1, e) 3, f) 5 µm of **C2** for 24 h followed by staining with JC‐1. g) The pixel ratio (red/green) for each set of cells is plotted in the bar graph. h) HCT116 cells and i) RKO cells were cultured in two different HBSS solutions (with/without chloride ions) followed by treatment of **C2** (0–5 µm) for 72 h. Cell viability was assessed by MTT assay. Error bars represent mean ± SEM. In (a), (b), (g) one‐way ANOVA was performed (*n* = 3); in h,i) two‐way ANOVA was performed (*n* = 3). ^*^ *p* < 0.05; ^**^ *p* < 0.01; ^***^ *p* < 0.001, n.s., not significant.

Having successfully constructed the artificial ion channel in LUVs and a planar lipid bilayer membrane, we investigated the ion transmembrane transport activity of metallacage **C2** in living cells. Intracellular Cl^−^ concentration was measured via a sensitive and specific chloride fluorescent probe, N‐(Ethoxycarbonylmethyl)−6‐methoxyquinolinium bromide (MQAE),^[^ ^8c^ ^]^ whose fluorescence intensity decreases as the amount of chloride ions increases. As shown in **Figure** **5**a,b, the fluorescence intensity of MQAE was significantly reduced in human colon carcinoma HCT116 and RKO cell lines after treating with **C2** (0, 1, 2, 3, 4, and 5 µm), while a control experiment with the same dye for ligand **L1** under identical conditions showed no change in fluorescence intensity of MQAE (Figure S15a,b, Supporting Information). These results suggested that **C2** acted as an artificial ion channel and effectively triggered influx of chloride ion in cancer cells.

The transcellular membrane chloride ion transport activity of **C2** further inspired us to investigate the impact of Cl^−^ transport in cell viability. Recent research on synthetic ion channels has shown that selective ion transport leads to apoptosis of cancer cells by altering pH or disrupting cell ion balance.^[^ ^8^, ^28^ ^]^ JC‐1 is a reliable and specific fluorescent probe for detecting mitochondrial membrane potential change in the early stage of cell apoptosis, so it is widely used for the monitoring of apoptotic cell death.^[^ ^29^ ^]^ Apoptotic cells were observed by a reduced ratio of red to green fluorescence. A stepwise dose‐dependent decrease of red fluorescence, with a concurring increase in green fluorescence, was observed in HCT116 cells upon treatment with **C2** (Figure 5c–g). On the other hand, no significant difference in the ratio of red to green fluorescence was detected upon treatment by the ligand **L1** (Figure S16a–e, Supporting Information). In addition, human colorectal carcinoma (HCT116, RKO, and HCT8 cells) and lung adenocarcinoma (epithelial A549 cells) were incubated with **C2** and **L1** for 24 h, followed by an evaluation of cell viability using an MTT assay. Significant cell death was observed when cancer cell lines were treated with **C2**, but not for those treated with **L1** (Figure S17, Supporting Information). Collectively, these results indicated that artificially constructed ion channels **C2** increased intracellular Cl^−^ levels and triggered cancer cell apoptosis.

In addition, to examine whether **C2**‐induced cancer cell death depended on chloride homeostasis, two distinct types of Hank's balanced salt solution (HBSS), one containing chloride and the other chloride‐free, were prepared.^[^ ^8c^ ^]^ HCT116 cells were incubated with one of the two HBSS solutions as an extracellular medium, and each cell was treated with different concentrations of **C2** (0, 1, 2, 3, 4, and 5 µm). Significantly higher cytotoxic effects of **C2** upon incubation of chloride ions containing extracellular medium were observed (Figure 5h,i). The cytotoxicity of compound **C2** against human normal cell lines (human embryonic kidney HEK293 and human colon epithelial NCM460) has been determined as well. As shown in Figure S17c,d (Supporting Information), compound **C2** has no cytotoxicity against HEK293 and NCM460 cells. These data highlight the potential applicability of the cage **C2** for treating cancer. The discrepancy in cell viability with or without chloride ions clearly suggested that cellular chloride ion transport was a critical contributor to **C2**’s cytotoxic effects.

## 3 Conclusion

We constructed the metallacage‐based ion channel **C2** from NDI organic precursors and Zn(II) metal salts through subcomponent self‐assembly. The efficient and selective transmembrane transport of anions might be due to the anion‐π and electrostatic interactions between **C2** and anions. Detailed MD simulations also demonstrated that **C2** is stably embedded into the phospholipid bilayer as an unimolecular channel at ambient temperature and pressure. Furthermore, the artificial unimolecular channel **C2** was active, with the EC_50_ value for chloride transport reaching 1.69 µm. The channel‐mediated Cl^−^ transmembrane transport across HCT116 and RKO cancer cells, disturbed their ion balance and inhibited their further growth at low concentrations. It should be noted that this work demonstrates the first case utilizing an artificial ion channel based on a metallacage for ion transport in living cells.

[CCDC 2 255 701 (for ligand **L1**) contains the supplementary crystallographic data for this paper. These data can be obtained free of charge from The Cambridge Crystallographic Data Centre via www.ccdc.cam.ac.uk/data_request/cif.]

## Conflict of Interest

The authors declare no conflict of interest.

## Supporting information

Supporting Information

## Data Availability

Data sharing is not applicable to this article as no new data were created or analyzed in this study.
