# Supplementary material for: Naphthalene Diimide‐Based Metallacage as an Artificial Ion Channel for Chloride Ion Transport
Source: Adv Sci (Weinh). 2024 Mar 8;11(21):2308181. doi: 10.1002/advs.202308181 (PMC11151027; doi:10.1002/advs.202308181)
Supplement: Supplementary file 1 — Supporting Information [file ADVS-11-2308181-s001.pdf]

ADVANCED SCIENCE
Open Access

Supporting Information

for *Adv. Sci.*, DOI 10.1002/advs.202308181

Naphthalene Diimide-Based Metallacage as an Artificial Ion Channel for Chloride Ion Transport

Qing-Hui Ling, Yuanyuan Fu, Zhen-Chen Lou, Bangkun Yue, Chenxing Guo, Xinyu Hu, Weiqiang Lu*, Lianrui Hu, Wei Wang, Min Zhang, Hai-Bo Yang and Lin Xu**

Supporting Information

Naphthalene Diimide-Based Metallacage as an Artificial Ion Channel for Chloride Ion Transport

Qing-Hui Ling, Yuanyuan Fu, Zhen-Chen Lou, Bangkun Yue, Chenxing Guo, Xinyu Hu, Weiqiang Lu*, Lianrui Hu, Wei Wang, Min Zhang, Hai-Bo Yang, Lin Xu**

Table of Contents

1. Materials and methods
2. Synthesis of ligands and metallacages
3. Variable temperature ^{1}H NMR spectra of **C3**
4. The UV-vis and fluorescence spectra of ligand **L1** and metallacages
5. ^{1}H NMR titration experiments with TBACl, TBABr, $TBANO_3$
6. Job Plot and UV-vis titration experiments of TBACl, TBABr, $TBANO_3$
7. Preparation of HPTS-entrapped large unilamellar vesicles.
8. Determination of cation and anion selectivity sequence through HPTS assay
9. Calcein-encapsulated large unilamellar vesicles
10. Conductance measurement in planar lipid bilayer
11. The molecular dynamic (MD) simulation of **C2** in lipid bilayer
12. Biological experiment
13. The ^{1}H NMR, ^{13}C NMR, COSY NMR, NOESY NMR DOSY NMR, and ESI-MS spectra of ligands and metallacages
14. References

1. Materials and methods

All reagents were of analytical purity and used without further treatment. TLC analyses were performed on silica-gel plates, and flash chromatography was conducted using silica-gel column packages. 1,2-diacyl-*sn*-glycero-3-phosphocholine (PC) were obtained from Avanti Polar Lipids as a solution (100 mg/mL in $CHCl_3$). HEPES buffer, HPTS, calcein, Triton X-100, NaOH and inorganic salts were purchased of molecular biology grade from Sigma and Aladdin.

^{1}H NMR and ^{13}C NMR spectra were recorded on Bruker 400 MHz Spectrometer (^{1}H: 400 MHz; ^{13}C: 101 MHz) or Bruker 500 MHz Spectrometer (^{1}H: 500 MHz; ^{13}C: 126 MHz) at 298 K. The ^{1}H NMR chemical shifts are reported relative to residual solvent signals. Coupling constants (J) are denoted in Hz and chemical shifts (δ) are denoted in ppm. Multiplicities are denoted as follows: s = singlet, d = doublet, m = multiplet. ESI-MS and TWIM-MS spectra were collected on a Waters Synapt G2-Si tandem mass spectrometer, using solutions of 1 mg sample in 1 mL of CH_3CN for cages **C1-C4**. Crystallographic data were collected using Rigaku XtaLAB PRO MM003-DS dual System. UV-vis spectra were recorded in a quartz cell (light path 10 mm) on a Shimadzu UV2700 UV-visible spectrophotometer. Steady-state fluorescence spectra were recorded in a conventional quartz cell (light path 10 mm) on a Shimadzu RF-6000 fluorescence spectrophotometer. The conductance measurement on planar lipid bilayer was performed on Warner Planar Lipid Bilayer Workstation.

2. Synthesis of ligands and metallacages

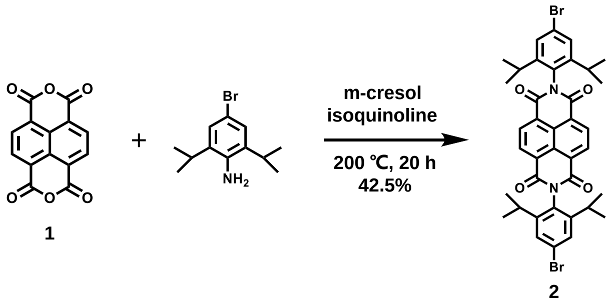

Scheme S1. Synthesis of compound **2**.

Compounds **2** was synthesized according to the literature.[1]

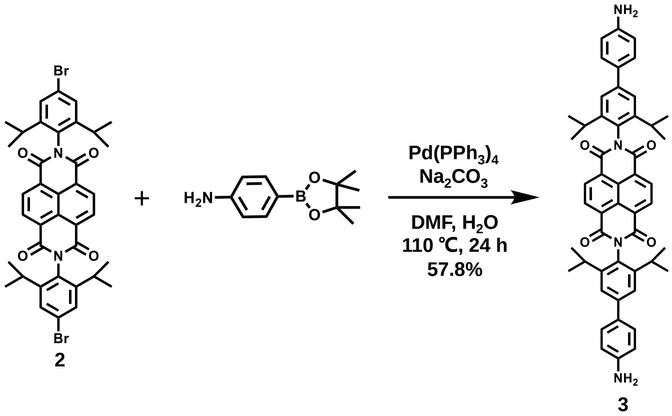

Scheme S2. Synthesis of compound **3**.

A mixture of compounds **2** (500 mg, 0.67 mmol) and 4-Aminophenylboronic acid pinacol ester (442 mg, 2.02 mmol) and Tetrakis(triphenylphosphine)palladium (80 mg, 0.067 mmol) and sodium carbonate (140 mg, 1.34 mmol) were added in a Schlenk flask, and the Schlenk flask was evacuated and back-filled with N_2 three times. Then the DMF (90 mL) and distilled water (30 mL) were bubbled under nitrogen for 30 min. Next, the solvent was added to the mixing system with a syringe under an inert atmosphere. The reaction was stirred 24 h at 110 °C. The solvent was evaporated and the residue was purified by column chromatography on silica gel (dichloromethane: methanol = 600 : 1, v/v) to give **3** as a dark pink solid (226 mg, 54.7%). **^{1}H NMR** (500 MHz, $CDCl_3$) δ (ppm): 8.90 (s, 2H), 7.50 -7.45 (m, 4H), 6.80 (d, J = 8.4 Hz, 2H), 3.78 (s, 2H), 2.73 (dt, J = 13.6, 6.8 Hz, 2H), 1.21 (d, J = 6.8 Hz, 12H).**^{13}C NMR** (126 MHz, $CDCl_3$) δ (ppm): 163.20, 145.76, 142.93, 131.94, 131.76, 128.51, 127.14, 122.79, 115.49, 29.62, 24.17.

ESI-HRMS *m/z***:** Calculated for [**3** + H]$^+$: 769.3676; Found: 769.3734.

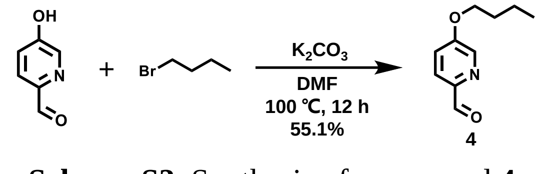

Scheme S3. Synthesis of compound **4**.

A mixture of 5-hydroxypyridine-2-carbaldehyde (1.0 g, 8.12 mmol), anhydrous potassium carbonate (1.1 g, 8.12 mmol), 1-bromobutane (0.9 ml, 8.12 mmol) were stirred vigorously in dry N, N-dimethylformamide (50 ml) at 100 °C for 12 h. The solvent was evaporated and the residue was purified by column chromatography on silica gel (petroleum ether : ethyl acetate = 300 : 9, v/v) to give **4** as a light yellow liquid (805 mg, 55.1%). **^{1}H NMR** (400 MHz, CD_3CN) δ (ppm): 9.93 (s, 1H), 8.44 (s, 1H), 7.93 (d, J = 8.7 Hz, 1H), 7.43 (d, J = 8.6 Hz, 1H), 4.17 (t, J = 6.5 Hz, 2H), 1.87-1.75 (m, 2H), 1.57-1.46 (m, 2H), 1.00 (t, J = 7.4 Hz, 3H).**^{13}C NMR** (101 MHz, CD_3CN) δ (ppm): 192.62, 159.34, 146.77, 139.49, 122.51, 117.87, 69.25, 31.27, 19.34,

13.61.

ESI-HRMS *m/z*: Calculated for [**4** + H]$^+$: 180.0947; Found: 180.1018.

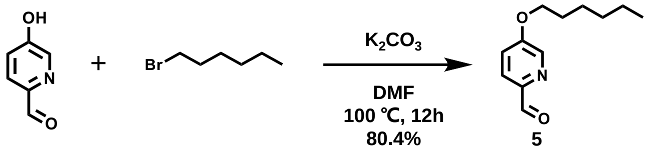

Scheme S4. Synthesis of compound **5**.

A mixture of 5-hydroxypyridine-2-carbaldehyde (1.0 g, 8.12 mmol), anhydrous potassium carbonate (1.1 g, 8.12 mmol), 1-hromohexane (1.14 ml, 8.12 mmol) were stirred vigorously in dry N, N-dimethylformamide (50 ml) at 100 °C for 12 h. The solvent was evaporated and the residue was purified by column chromatography on silica gel (petroleum ether : ethyl acetate = 300 : 9, v/v) to give **5** as a light yellow liquid (1.3506 g, 80.4%). **^{1}H NMR** (400 MHz, CD_3CN) δ (ppm): δ 9.90 (s, 1H), 8.41 (s, 1H), 7.90 (d, J = 8.7 Hz, 1H), 7.40 (dd, J = 8.8 Hz, 1H), 4.13 (t, J = 6.6 Hz, 2H), 1.79 (p, J = 6.9 Hz, 2H), 1.46 (m, 2H), 1.35 (m, 4H), 0.95-0.87 (m, 3H). **^{13}C NMR** (101 MHz, CD_3CN) δ (ppm): 192.99, 159.73, 147.15, 139.88, 124.16, 121.64, 69.93, 32.19, 29.60, 26.19, 23.28, 14.29.

ESI-HRMS *m/z*: Calculated for [**5** + H]$^+$: 208.1259; Found: 208.1332.

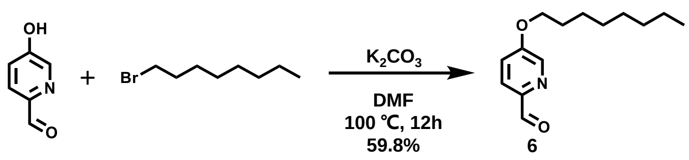

Scheme S5. Synthesis of compound **6.**

A mixture of 5-hydroxypyridine-2-carbaldehyde (1.0 g, 8.12 mmol), anhydrous potassium carbonate (1.1 g, 8.12 mmol), 1-bromooctane (1.4 ml, 8.12 mmol) were stirred vigorously in dry N, N-dimethylformamide (50 ml) at 100 °C for 12 h. The solvent was evaporated and the residue was purified by column chromatography on silica gel (petroleum ether : ethyl acetate = 300 : 9, v/v) to give **6** as a brownish yellow liquid (1.1852 g, 59.8%). **^{1}H NMR** (500 MHz, CD_3CN) δ (ppm): 9.90 (s, 1H), 8.41 (s, 1H), 7.90 (d, J = 8.6 Hz, 1H), 7.40 (dd, J = 8.6, 2.9, Hz, 1H), 4.13 (t, J = 6.6 Hz, 2H), 1.79 (q, J = 7.9, Hz, 2H), 1.50-1.41 (m, 2H), 1.40-1.23 (m, 8H), 0.92-0.84 (m, 3H). **^{13}C NMR** (126 MHz, CD_3CN) δ (ppm): 192.97 , 159.67 , 147.05 , 139.82 , 122.86 (d, J = 322.7 Hz), 69.86 , 32.48 , 29.88 , 29.56 , 26.45 , 23.30 , 14.32 .

ESI-HRMS *m/z*: Calculated for [**6** + H]$^+$: 236.1572; Found: 236.1647.

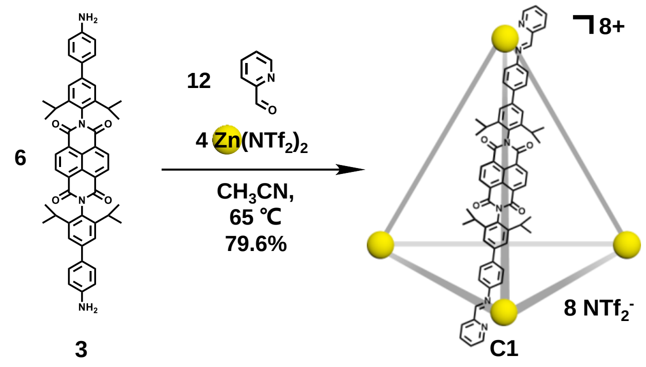

Scheme S6. Self-assembly of metal coordination cage **C1**.

Subcomponent **3** (30.0 mg, 0.039 mmol), 2-pyridinecarboxaldehyde (8.4 mg, 0.078 mmol), and $Zn(NTf_2)_2$ (16.3 mg, 0.026 mmol) were combined in CH_3CN (6 mL) and stirred at 65 °C overnight. The solvent was evaporated mostly and diethyl ether (10 mL) was added to get precipitated suspension. Then the mixture centrifuged and the supernatant diethyl ether was decanted. This procedure was repeated three times with fresh diethyl ether. The residue was then dried in vacuo to afford the solid product **C1** as faint yellow powder (42.4 mg, 79.6%).**^{1}H NMR** (500 MHz, CD_3CN) δ (ppm): 8.78 (d, J = 11.4 Hz, 2H), 8.72-8.63 (m, 1H), 8.52 (t, J = 7.1 Hz, 1H), 8.34-8.24 (m, 1H), 8.08 (dd, J = 11.9, 7.0 Hz, 1H), 7.95 (d, J = 4.8 Hz, 1H), 7.81-7.72 (m, 2H), 7.71-7.65 (m, 2H), 6.44-6.28 (m, 2H), 2.91-2.80 (m, 2H), 1.17 (d, J = 6.6 Hz, 12H).**^{13}C NMR** (126 MHz, CD_3CN) δ (ppm): 164.34, 148.07, 147.49, 143.66, 132.16, 131.69, 129.45, 128.67, 127.96, 123.94, 122.86, 122.10, 119.56, 29.94, 24.15, 24.11.

ESI-MS: calcd for [**C1**-5Tf_2N^-]$^{5+}$: m/z = 1356.3995, found: 1356.3999; calcd for [**C1**-6Tf_2N^-]$^{6+}$: m/z = 1084.3933, found: 1084.3441; calcd for [**C1**-7Tf_2N^-]$^{7+}$: m/z = 889.1673, found: 889.1910; calcd for [**C1**-8Tf_2N^-]$^{8+}$: m/z = 743.0475, found: 743.3036.

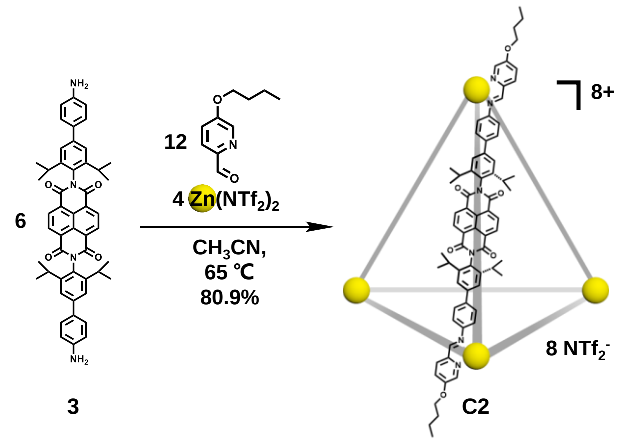

Scheme S7. Self-assembly of metal coordination cage **C2**.

Subcomponent **3** (30.0 mg, 0.039 mmol), **4** (14.0 mg, 0.078 mmol), and $Zn(NTf_2)_2$ (16.3 mg, 0.026 mmol) were combined in CH_3CN (6 mL) and stirred at 65 °C overnight. The solvent was

evaporated mostly and diethyl ether (10 mL) was added to get precipitated suspension. Then the mixture centrifuged and the supernatant diethyl ether was decanted. This procedure was repeated three times with fresh diethyl ether. The residue was then dried in vacuo to afford the solid product **C2** as faint yellow powder (47.6 mg, 80.9%).^{1}**H NMR** (500 MHz, CD_3CN) δ (ppm): 8.77 (s, 2H), 8.60-8.47 (m, 1H), 8.24-8.16 (m, 1H), 7.91 (d, J = 8.7 Hz, 1H), 7.82-7.60 (m, 5H), 6.44- 6.25 (m, 2H), 4.22 (s, 2H), 2.91-2.78 (m, 2H), 1.82 (d, J = 6.1 Hz, 2H), 1.51 (dd, J = 13.6, 6.2 Hz, 2H), 1.17 (d, J = 6.7 Hz, 12H), 1.00 (t, J = 7.3 Hz, 3H). ^{13}**C NMR** (126 MHz, CD_3CN) δ (ppm): 164.44, 161.69, 148.10, 142.14, 141.03, 139.88, 132.25, 131.65, 129.36, 128.73, 128.03, 124.83, 123.97, 123.19, 122.16, 119.62, 70.73, 31.46, 30.01, 24.21, 19.69, 14.01.

ESI-MS: calcd for [**C2**-5Tf$_2$N$^-$]$^{5+}$: m/z = 1529.5377, found: 1529.5118; calcd for [**C2**-6Tf$_2$N$^-$]$^{6+}$: m/z = 1228.6311, found: 1228.6042; calcd for [**C2**-7Tf$_2$N$^-$]$^{7+}$: m/z = 1012.6945, found: 1012.7078; calcd for [**C2**-8Tf$_2$N$^-$]$^{8+}$: m/z = 851.6171, found: 851.6282.

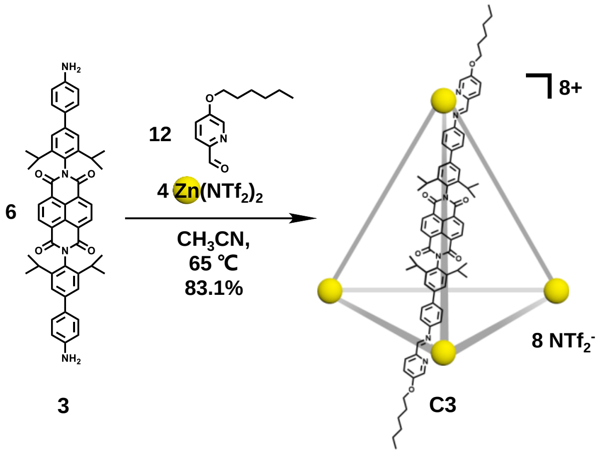

Scheme S8. Self-assembly of metal coordination cage **C3**.

Subcomponent **3** (50.0 mg, 0.065 mmol), **5** (27.0 mg, 0.13 mmol), and $Zn(NTf_2)_2$ (27.0 mg, 0.043 mmol) were combined in CH_3CN (8 mL) and stirred at 65 °C overnight. The solvent was evaporated mostly and diethyl ether (10 mL) was added to get precipitated suspension. Then the mixture centrifuged and the supernatant diethyl ether was decanted. This procedure was repeated three times with fresh diethyl ether. The residue was then dried in vacuo to afford the solid product **C3** as faint yellow powder (84.5 mg, 83.1%).^{1}**H NMR** (500 MHz, CD_3CN) δ (ppm): 8.77 (s, 2H), 8.59-8.48 (m, 1H), 8.25-8.16 (m, 1H), 7.90 (d, J = 8.7 Hz, 1H), 7.79-7.61 (m, 5H), 6.45-6.24 (m, 2H), 4.21 (s, 2H), 2.92-2.81 (m, 2H), 1.83 (d, J = 6.1 Hz, 2H), 1.48 (s, 2H), 1.37 (s, 4H), 1.15 (dd, J = 20.1, 6.9 Hz, 12H), 0.94 (d, J = 6.2 Hz, 3H). ^{13}**C NMR** (126 MHz, CD_3CN) δ (ppm): 164.35, 161.63, 148.03, 142.03, 140.98, 139.81, 132.17, 131.60,

129.31, 128.68, 127.98, 124.78, 123.92, 123.12, 122.13, 119.58, 70.99, 32.11, 29.95, 29.34, 26.09, 24.16, 23.25, 14.30.

ESI-MS: calcd for [**C3**-5Tf_2N^-]$^{5+}$: *m/z* = 1596.6097, found: 1596.6567; calcd for [**C3**-6Tf_2N^-]$^{6+}$: *m/z* = 1284.3645, found: 1284.3605; calcd for [**C3**-7Tf_2N^-]$^{7+}$: *m/z* = 1060.8888, found: 1060.7574; calcd for [**C3**-8Tf_2N^-]$^{8+}$: *m/z* = 893.6641, found: 893.6743.

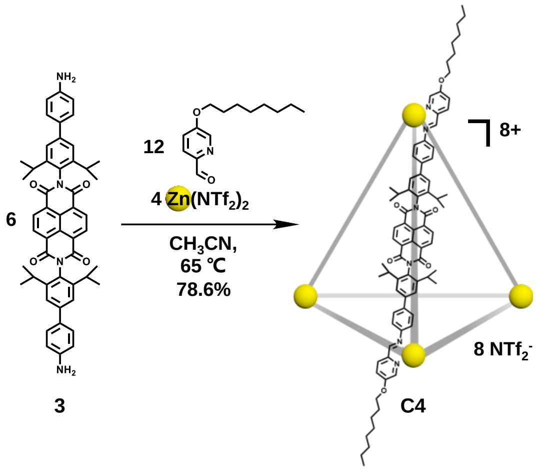

Scheme S9. Self-assembly of metal coordination cage **C4**.

Subcomponent **3** (30.0 mg, 0.039 mmol), **6** (18.4 mg, 0.078 mmol), and $Zn(NTf_2)_2$ (16.3 mg, 0.026 mmol) were combined in CH_3CN (6 mL). The solvent was evaporated mostly and diethyl ether (10 mL) was added to get precipitated suspension. Then the mixture centrifuged and the supernatant diethyl ether was decanted. This procedure was repeated three times with fresh diethyl ether. The residue was then dried in vacuo to afford the solid product **C4** as faint yellow powder (50.0 mg, 78.6%).**^{1}H NMR** (500 MHz, CD_3CN) *δ* (ppm): 8.77 (s, 2H), 8.62-8.45 (m, 1H), 8.21 (qd, *J* = 10.2, 5.0 Hz, 1H), 7.91 (d, *J* = 8.8 Hz, 1H), 7.79-7.60 (m, 5H), 6.46-6.23 (m, 2H), 4.21 (s, 2H), 2.90 -2.82 (m, 2H), 1.82 (d, *J* = 5.1 Hz, 2H), 1.47 (d, *J* = 5.4 Hz, 2H), 1.40-1.30 (m, 8H), 1.20-1.09 (m, 12H), 0.92 (t, *J* = 6.7 Hz, 3H). **^{13}C NMR** (126 MHz, CD_3CN) *δ* (ppm): 164.36, 161.64, 148.05, 142.04, 140.96, 139.83, 132.95, 132.18, 131.61, 129.33, 129.20, 128.69, 127.99, 124.82, 123.93, 123.13, 122.14, 119.59, 70.99, 32.53, 29.93, 29.90, 26.42, 24.20, 24.17, 24.14, 23.25, 14.38. **ESI-MS**: calcd for [**C4**-5Tf_2N^-]$^{5+}$: *m/z* =1664.8850, found: 1664.8518; calcd for [**C4**-6Tf_2N^-]$^{6+}$: *m/z* = 1340.0873, found: 1340.0836; calcd for [**C4**-7Tf_2N^-]$^{7+}$: *m/z* = 1108.9449, found: 1108.9467; calcd for [**C4**-8Tf_2N^-]$^{8+}$: *m/z* = 935.8361, found: 935.8251.

3. Variable temperature ^{1}H NMR spectra of C3

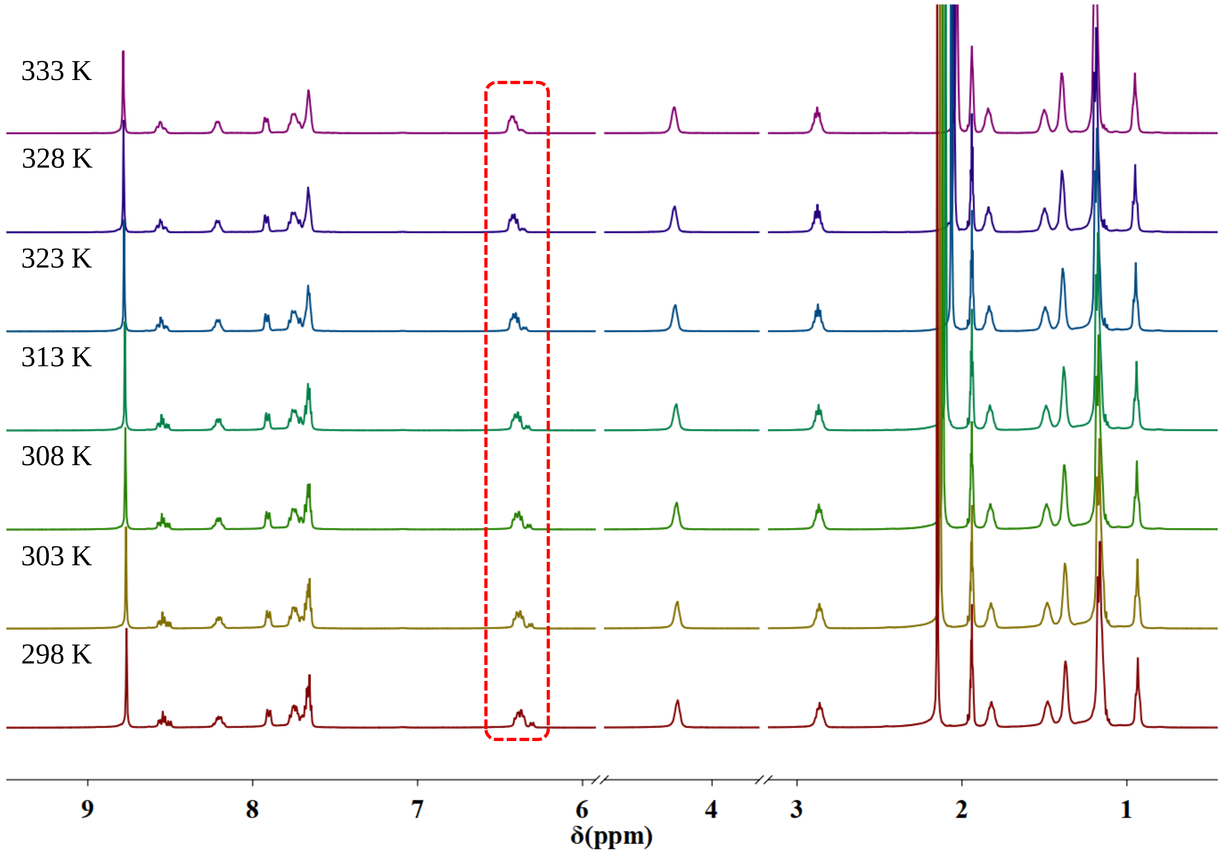

Figure S1. ^{1}H NMR (500 MHz, CD_3CN) spectra of **C3** at variable temperature from 298 K to 333 K.

4. The UV-vis and fluorescence spectra of ligand and metallacages

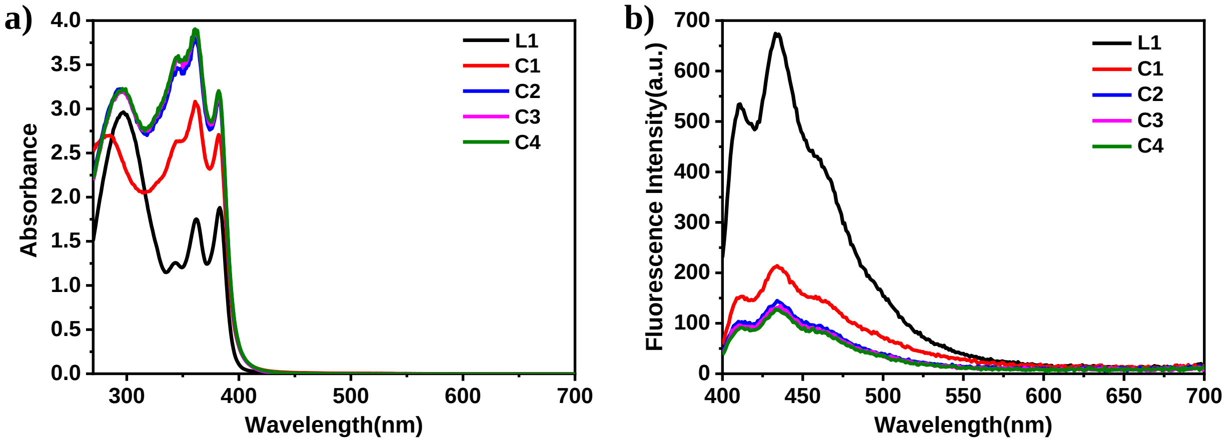

Figure S2. a) The UV-vis and b) fluorescence spectra of ligands **L1** (60 μM) and metallacages **C1**, **C2**, **C3**, **C4** (10 μM) in DMF (5 nm, 5 nm).

5. ^{1}H NMR titration experiments with TBACl, TBABr, $TBANO_3$

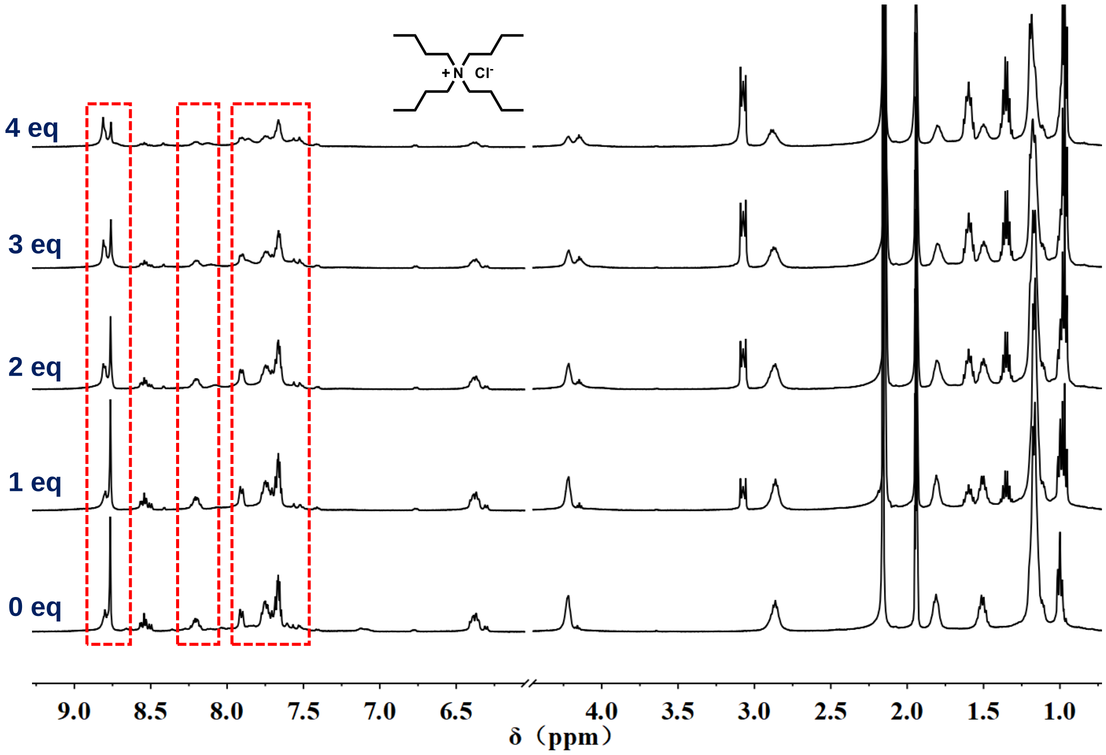

Figure S3. ^{1}H NMR (500 MHz, CD_3CN, 298 K) titration experiments of a 1 mM solution of cage **C2** with TBACl.

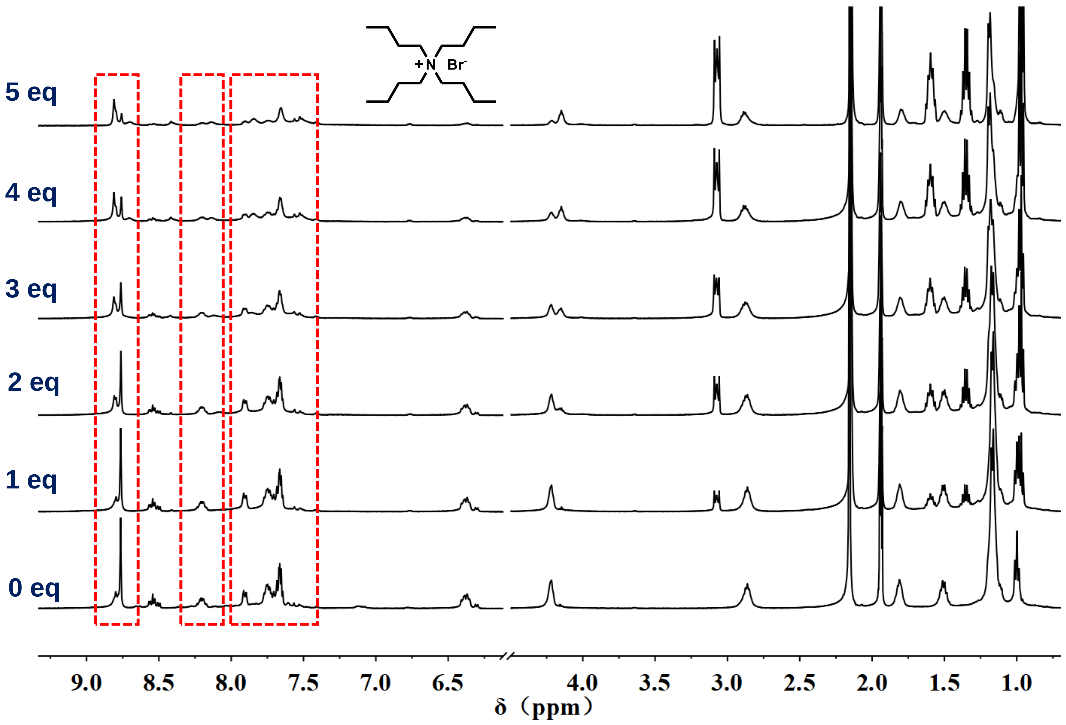

Figure S4. ^{1}H NMR (500 MHz, CD_3CN, 298 K) titration experiments of a 1 mM solution of cage **C2** with TBABr.

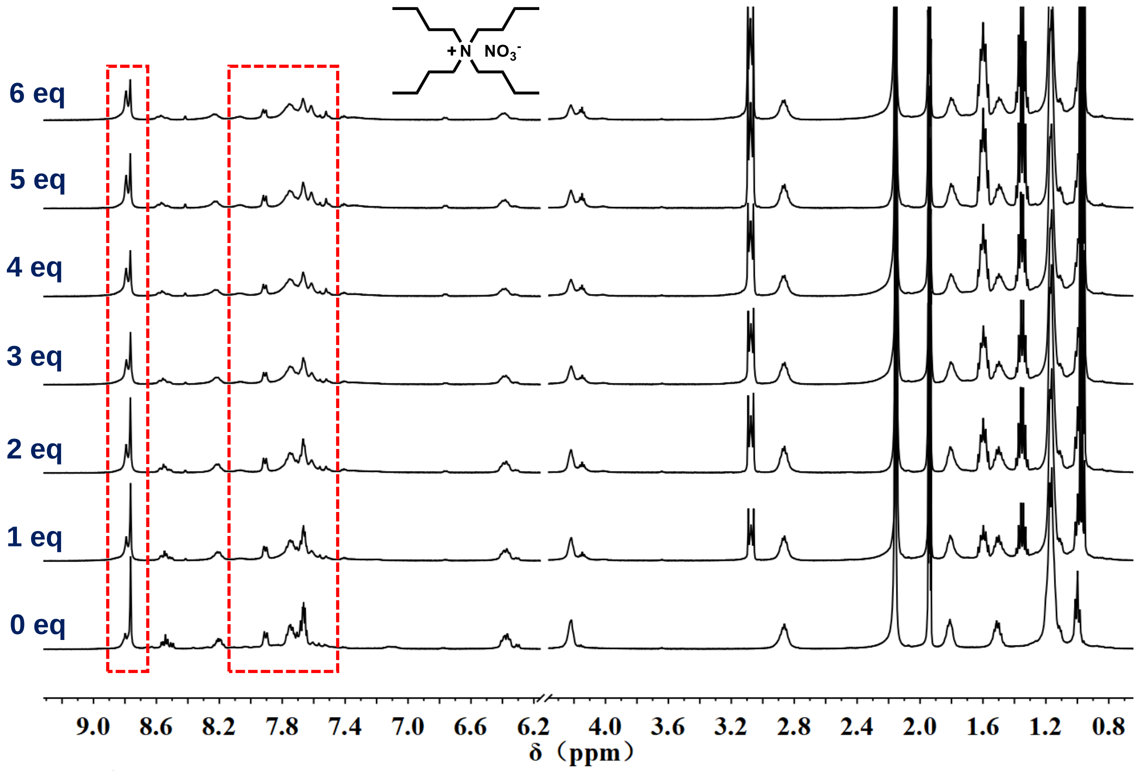

Figure S5. ^{1}H NMR (500 MHz, CD_3CN, 298 K) titration experiments of a 1 mM solution of cage **C2** with $TBANO_3$.

6. Job Plot and UV-vis titration experiments of TBACl, TBABr, $TBANO_3$

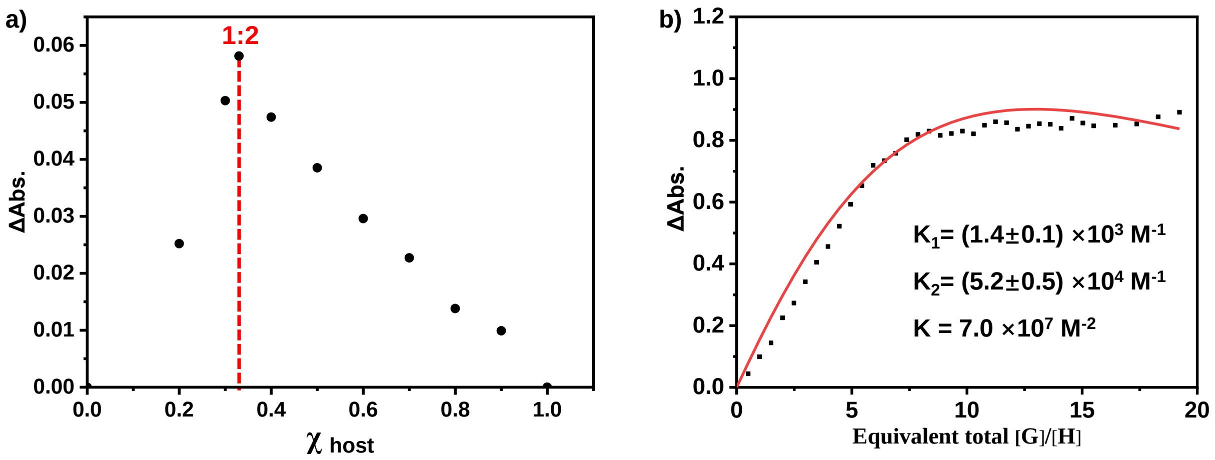

Figure S6. a) Job plot for the complexation of **C2** and TBACl based on the absorbance at 393 nm in CH_3CN. [**C2**] + [Cl^-] = 20 μM. b) Determination of the association constant of **C2** (10 μM) and Cl^- by 1:2 binding model in CH_3CN. Fitting result based on the absorbance at 329 nm.

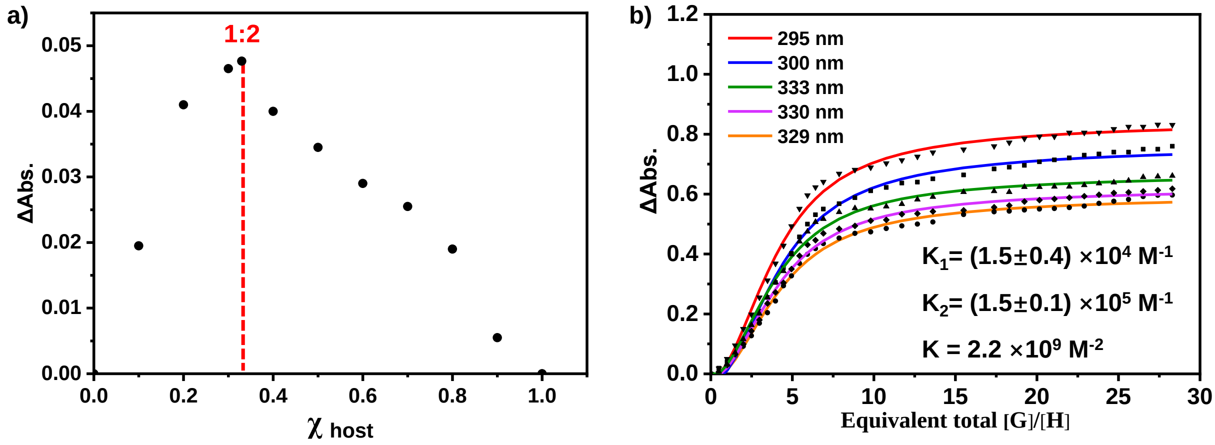

Figure S7. a) Job plot for the complexation of **C2** and TBABr based on the absorbance at 390 nm in CH_3CN. [**C2**] + [Br^-] = 20 µM. b) Determination of the association constant of **C2** (10 µM) and Br^- by 1:2 binding model in CH_3CN. Fitting result based on the absorbance at 295 nm, 300 nm, 333 nm, 330 nm and 329 nm.

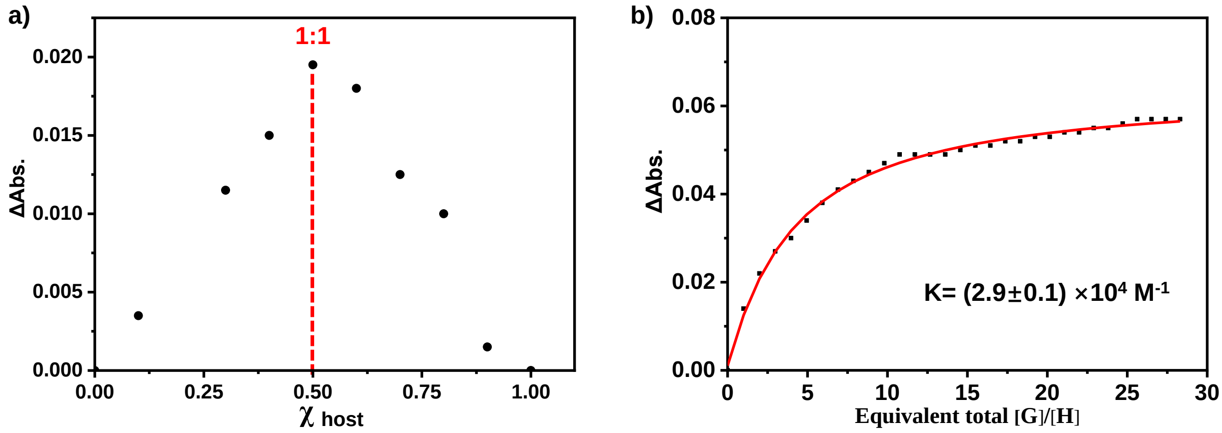

Figure S8. a) Job plot for the complexation of **C2** and $TBANO_3$ based on the absorbance at 390 nm in CH_3CN. [**C2**] + [NO_3^-] = 20 µM. b) Determination of the association constant of **C2** (10 µM) and NO_3^- by 1:1 binding model in CH_3CN. Fitting result based on the absorbance at 395 nm.

7. Preparation of HPTS-entrapped large unilamellar vesicles.

1,2-diacyl-sn-glycero-3-phosphocholine (PC, 100 mg/ml, 0.15 mL) and 3-*β*-hydroxy-5-cholestene (CH, 3.75 mg) were dissolved in $CHCl_3$ (10 mL) in a round-bottom flask. The solvent was removed under reduced pressure (5 min, 20 °C) to produce a uniform thin film. The film was dried under high vacuum for 3 h at room temperature. Then the film was hydrated with 4-(2-hydroxyethyl)-1-piperazineethanesulfonic acid (HEPES) buffer solution (1.5 mL, 10 mM HEPES, 100 mM NaCl, pH = 7.2) containing a pH sensitive dye 8-hydroxypyrene-1,3,6-

trisulfonic acid (HPTS, 0.1 mM) in thermostatic shaker-incubator at 37 °C for 2 h to give a milky suspension. The mixture was then subjected to ten freeze-thaw cycles: freeze in liquid N_2 for 30 s, warm it up at 37 °C for 1.5 min, then gentle vortex mixing for 3 min at room temperature. The vesicle suspension was extruded through polycarbonate membrane (0.22 μM) to produce homogeneous suspension of large unilamellar vesicles (LUVs) with encapsulated HPTS. The suspension of LUVs was divided into two equal aliquots and dialyzed using membrane tube (MWCO = 8000-14000) against the same HEPES buffer solution (300 mL, without HPTS) for 36 h with gentle stirring (200 r/min). The dialysis was repeated for six times to remove free HPTS.

The aqueous interior of LUVs was buffered at pH = 7.2 (10 mM HEPES, 100 mM NaCl, ~0.1 mM HPTS), and the exterior was buffered at pH = 6.8 (10 mM HEPES, 100 mM NaCl). The solution of NDI metallacages in DMF (1.0 mM) was then added into the above suspension of HPTS-encapsulated LUVs, and the fluorescence emission of the dye was immediately monitored at room temperature. After 10 minutes, Triton X-100, a surfactant, was added to completely disrupt LUVs and achieve the minimum fluorescence emission of HPTS as the 100% reference point.

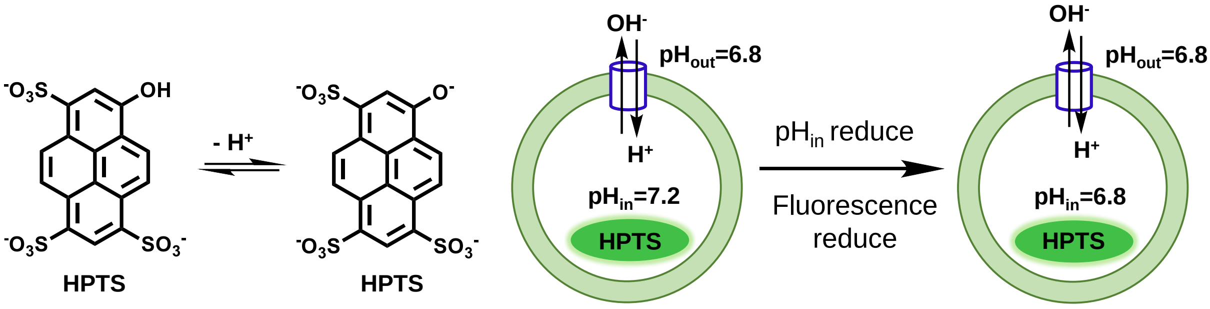

Figure S9. Schematic illustration of the mechanism of pH-sensitive HPTS fluorescence change.

Determination of ion transport activity through HPTS assay:

The prepared LUVs suspension (40 μL) was added to HEPES buffer solution (total volume 2000 μL, 10 mM HEPES, 100 mM NaCl, pH = 6.8), followed by the addition of metallacage solution in DMF (1.0 mM) with gentle mixing. The fluorescence intensity was then measured as a function of time to investigate the channeling activity of the metallacages. Fluorescence intensity of HPTS (I_1) was continuously monitored at 510 nm (excitation at 454 nm) for 10 min. Then, aqueous solution of Triton X-100 (16 μL, 20% v/v,) was added to completely disrupt LUVs and achieve the minimum changes in dye fluorescence emission (I_2). The collected data were then normalized into the fractional change in fluorescence intensity according to the

following equation: $R(\%) = \frac{I_0-I_1}{I_0-I_2} \times 100\%$ where I_0 is the initial intensity.

8. Determination of cation and anion selectivity sequence through HPTS assay

Cation selectivity sequence:

The HPTS-encapsulated LUVs (intravesicular buffer solution: 10 mM HEPES, ~0.1 mM HPTS, 100 mM NaCl, pH = 7.2) were prepared as described above. The extravesicular buffer solution with different cations (pH = 7.6, HEPES 10 mM, MCl 100 mM, M = Li^+, Na^+, K^+) was prepared and the pH was adjusted using MOH. The suspension of LUVs (13.3 mM, 40 μL) was then added to the extravesicular buffer solution, followed by the addition of the cage solution in DMF (metallacage **C2**: 0.9 mol% relative to lipid). The total volume of the assay solution was 2000 μL. The HPTS fluorescence change (I_t) was then continuously monitored at 510 nm (excitation at 454 nm) for 10 min. The aqueous solution of Triton X-100 (16 μL, 20% v/v) was added to achieve the minimum change in dye fluorescence emission (I_2) at the end of experiment. The collected data were normalized into the fractional change in fluorescence following the equation: $R(\%) = \frac{I_t-I_0}{I_2-I_0} \times 100\%$ where I_0 is the initial intensity.

Anion selectivity sequence:

The experiments were performed using the HPTS-encapsulated LUVs as described above. The extravesicular buffer solution containing different anions (pH = 6.8, HEPES 10 mM, NaX 100 mM, X = Cl^-, Br^-, NO_3^-, SO_4^{2-}) were prepared and the pH was adjusted to 6.8 using NaOH. The suspension of LUVs (13.3 mM, 40 μL) was then added to the extravesicular buffer solution, followed by the addition of the metallacage solution in DMF (cage **C2**: 0.9 mol% relative to lipid). The total volume of the assay solution was 2000 μL. The HPTS fluorescence change (I_t) was then continuously monitored at 510 nm (excitation at 454 nm) for 10 min. The aqueous solution of Triton X-100 (16 μL, 20% v/v) was added to achieve the minimum changes in dye fluorescence emission (I_2) at the end of experiment. The collected data were then normalized into the fractional change in fluorescence intensity following the equation: $R(\%) = \frac{I_0-I_t}{I_0-I_2} \times 100\%$ where I_0 is the initial intensity.

9. Calcein-encapsulated large unilamellar vesicles

PC (100 mg/mL, 0.10 mL) and CH (2.5 mg) were dissolved in $CHCl_3$ (20 mL). The solution was evaporated under reduced pressure (10 min, 20 °C), and further dried under high vacuum for 3 h. The lipid film was then hydrated with HEPES buffer solution (1.0 mL, 10 mM HEPES,

100 mM NaCl, pH = 7.4) containing calcein (40 mM) at 37 °C for 2 h in thermostatic shaker-incubator to give a milky suspension. Ten freeze-thaw cycles (freeze in liquid nitrogen for 30 s, warm it up at 37 °C for 1.5 min, and then gentle vortex mixing for 3 min) were performed. The LUVs suspension was extruded through polycarbonate membrane (0.22 μm) to produce homogeneous suspension of LUVs. The suspension of LUVs was divided into two equal aliquots and dialyzed using membrane tube (MWCO = 8000-14000) against the same HEPES buffer solution (300 mL, without calcein) for 36 h with gentle stirring (200 r/min). The dialysis was repeated for six times to remove free HPTS.

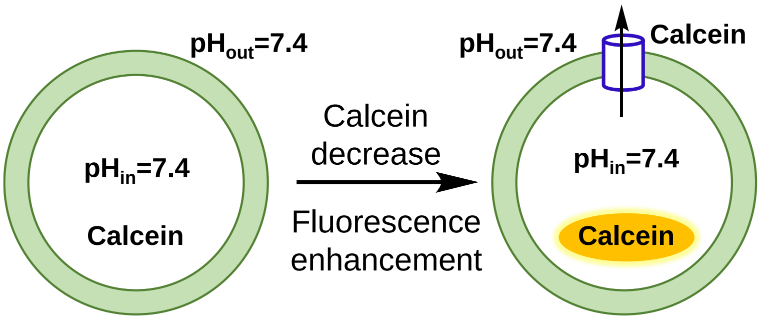

Figure S10. Schematic illustration of the concentration-sensitive calcein fluorescence change.

Determination of calcein transport through calcein assay:

Calcein was an indicator of lipid vesicle leakage. The above suspension of LUVs with entrapped-calcein (40 μL) was added to HEPES buffer solution (total volume 2000 μL, 10 mM HEPES, 100 mM NaCl, pH = 7.4), followed by the solution of metallacage (1.0 mM) in DMF with gentle mixing. Fluorescence intensity of calcein (I_1) was continuously monitored at 505 nm (excitation at 493 nm) for 5 min. Then, aqueous solution of Triton X-100 (16 μL, 20% v/v) was added to disrupt LUVS and achieve the maximum changes in dye fluorescence emission (I_2) at the end of experiment. The collected data were then normalized into the fractional change in fluorescence according to the following equation: $R(\%) = \frac{I_1 - I_0}{I_2 - I_0} \times 100\%$ where I_0 is the initial intensity.

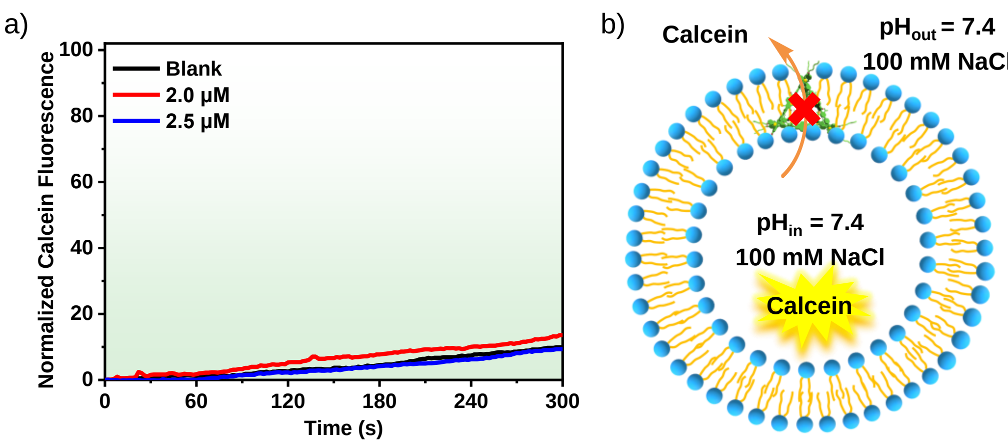

Figure S11. a) Calcein transport activities of **C2**, indicating calcein cannot be transported across membranes via **C2**; b) Schematic illustration of the calcein-sensitive LUVs ⊃ calcein assay.

10. Conductance measurement in planar lipid bilayer

The solution of the diPhyPC in chloroform (10 mg/ml, 20 μL) was evaporated with nitrogen gas to form a thin film and re-dissolved in *n*-decane (5 μL). The diPhyPC solution (0.5 μL) was injected on to the aperture (diameter = 200 μm) of the Delrin® cup (Warner Instruments, Hamden, CT) and then evaporated with nitrogen gas. In a typical experiment for measurement of the channel conductance, the chamber (*cis* side) and the Delrin cup (*trans* side) were filled with aqueous KCl solution (1.0 M, 1.0 mL). Ag-AgCl electrodes were applied directly to the two solutions and the *cis* one was grounded. Planar lipid bilayers were formed by painting the lipids solution (1.0 μL) around the pretreated aperture and by judgment of capacitance (80-120 pF). The solution of the test metallacage **C2** in DMF was added to the *cis* chamber and the solution was stirred for 5 min. Membrane currents were measured using a Warner BC-535D bilayer clamp amplifier and were collected by PatchMaster (HEKA) with sample interval at 5 kHz and then filtered with an 8-pole Bessel filter at 1 kHz (HEKA).[2] The data were analyzed by FitMaster (HEKA) with a digital filter at 100 Hz.

To claim anion selectivity, the corresponding permeability ratios P_{Cl^-}/P_{K^+} was calculated to be 3.6 and the permeability ratios of K^+ (P_{K^+}/P_{Cl^-}) was calculated to be 0.28. The ion permeability ratio between Cl^- and K^+ (P_{Cl^-}/P_{K^+}) was determined by measuring the reversal potential (Vr) in the salt gradient solution (1.0/0.30 M KCl, *trans/cis*). P_{Cl^-}/P_{K^+} was obtained using the equation derived from the Goldman–Hodgin–Katz voltage equation as follows.[3]

$$P_{Cl}/P_K = [a_{Kc} - a_{Kt}\exp(-V_rF/RT)]/[a_{Clc}\exp(-V_rF/RT) - a_{Clt}]$$

where a_{Kc} and a_{Kt} is the activity of K^+ in *cis* and *trans* chamber, respectively; a_{Clc} and a_{Clt} is the activity of Cl^- in *cis* and *trans* chamber, respectively; F is the Faraday constant; R is the gas constant; and T is the absolute temperature.

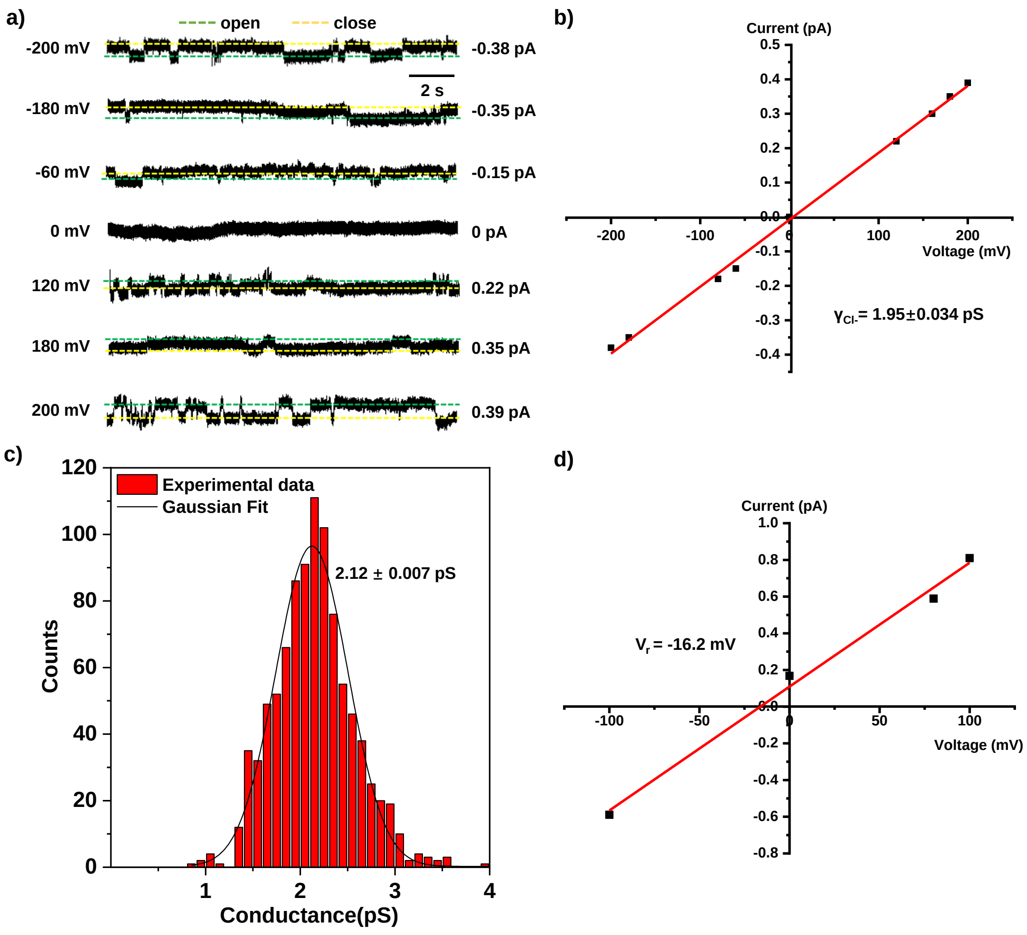

Figure S12. a) The recorded current profiles of **C2** from a single channel at different holding voltage (-200 mV to 200 mV) in a symmetrical 1 M KCl solution. The current value represents the mean value generated by the program Fitmaster. b) Determination of chloride conductance (γ_{Cl}^-) for **C2** using a linear *I-V* curve with the corresponding single current traces. c) Histogram of the conductance of **C2** obtained from the insertions and closures of the channel across a planar diPhyPC and a Gaussian fit to the data. Current was recorded in 1.0 M KCl at 200 mV. d) Linear fitting of *I-V* curve plot gives Cl^-/K^+ selectivity of 3.6 for **C2** transport of chloride.

11. The molecular dynamic (MD) simulation of C2 in lipid bilayer

All MD simulations were performed using the GROMACS 2019.6 package.[4] For **C2**, the MOPAC optimized structure using the PM6 semiempirical method was adopted as the initial conformation. RESP partial charges were obtained by fitting to the electrostatic potential of metallacage **C2** at the B3LYP/6-31G* level. For the membrane, a solvated lipid bilayer of diPhyPC with a total of 288 lipids (144 lipids in each layer) was built by genmixmem. The general AMBER force field (GAFF)[5] built by Multiwfn[6] and sobtop (http://sobereva.com/soft/Sobtop) was applied to the **C2** system, and the Lipid17 force field (https://zenodo.org/record/3610470#.ZFNrFmBlJzA) was used for the diPhyPC.

An energy minimization process was performed to prepare the membrane system, followed by equilibration for 80 ns in the NPT ensemble at 300 K. All bonds involving H atoms were constrained using the LINCS constraint algorithm, and integration of the equations of motion was achieved using the leap-frog algorithm with a time step of 2 fs. A constant temperature of 300 K was maintained by coupling the system to a Nose-Hoover thermostat.[7] For a constant pressure, semi-isotropic coupling was applied at 1 bar pressure bath using a Parrinello-Rahman barostat.[8] The Lenard Jones interactions were handled by the smooth particle-mesh Ewald (PME) [9] method with a cutoff radius of 1.2 nm.

To simulate **C2** embedded lipid bilayer system with a normal saline in a neutral simulation box, **C2** was embedded into the equilibrated lipid bilayer system contained with 56 chloride ions and 48 sodium ions. Except for Berendsen barostat,[10] the simulation parameters were kept the same as described above for the equilibration simulations. To equilibrate lipids and solvent around **C2**, 500 ps equilibration with harmonic position restraints (1000 $kJ\cdot mol^{-1}\cdot nm^{-2}$) was applied to all atoms of **C2** in the NPT ensemble.

Subsequent to the initial equilibration, the harmonic position restraint potentials were turned off, allowing for a more realistic modeling of the system. A 100 ns MD simulation was performed to obtain a well-equilibrated structure of **C2** within the hydrated lipid bilayer. Despite being initially randomly inserted into the lipid bilayer, **C2** eventually became stable within the bilayer.

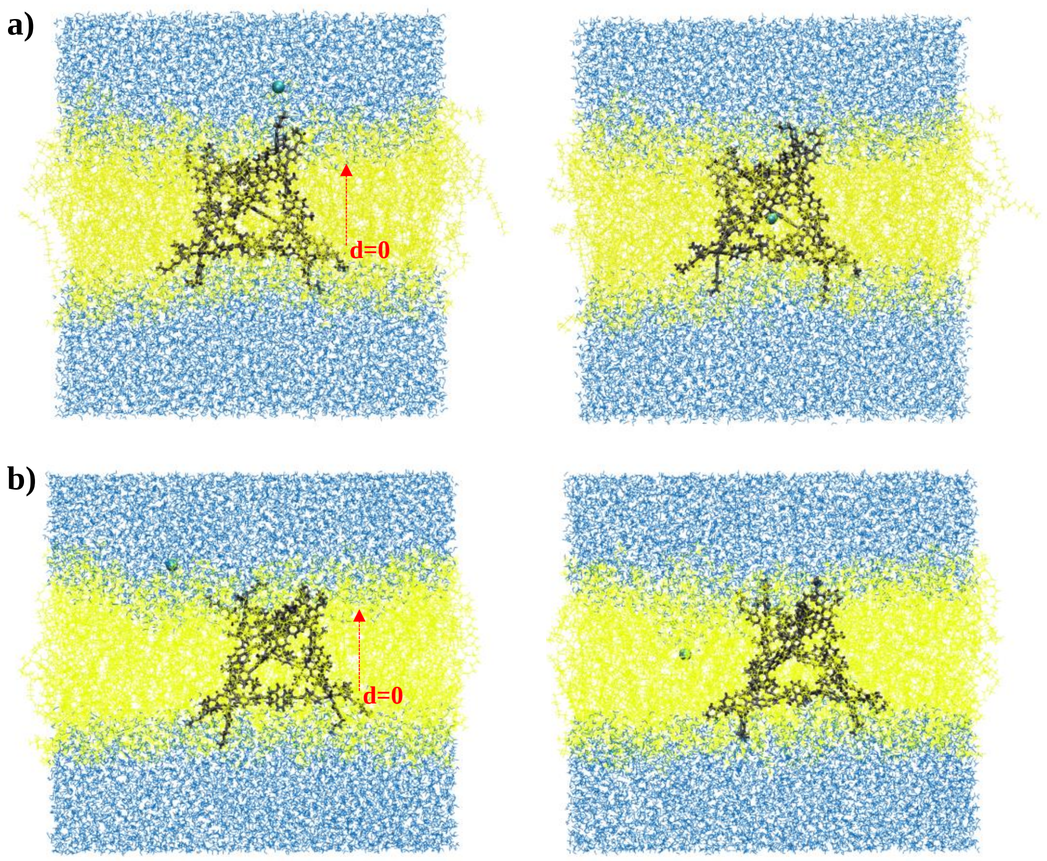

Figure S13. Snapshot of **C2** displaying the collective variable in umbrella simulation: a) Cl^- transport via the channel and b) Cl^- transport across the membrane without channel.

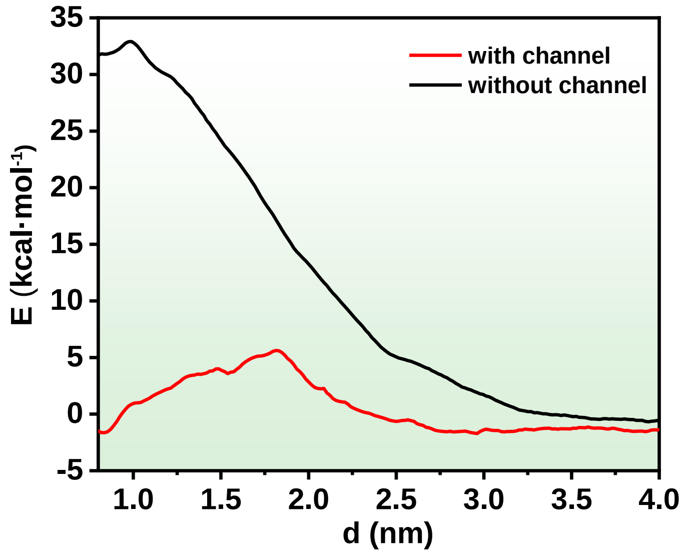

Figure S14. Free energy profile corresponding to Cl^- transport via the channel and across the membrane without any channel.

An umbrella sampling simulation was performed to explore the free energy associated with the ion movement through **C2**. Specifically, the movement of one chloride ion at the rate of 0.02 $nm \cdot ps^{-1}$ from aqueous phase to the inside of **C2** or lipid bilayer was simulated, respectively. To achieve this, the final equilibrated structure was taken. After generating 80 windows with the above procedure, we conducted simulations on the stored configurations for 500 ps by constraining the distance. Finally, the last 200 ps of the simulation was used to calculate the PMF.

12. Biological experiment

Cell culture

Human cancer cell lines (HCT116, RKO, HCT8 and A549), human embryonic kidney HEK293 cells and human colon epithelial NCM460 cells were purchased from National Infrastructure of Cell Line Resource (Shanghai, China). HCT116, RKO and HEK293 were cultured in Dulbecco's Modified Eagle Medium (DMEM, Gibco) and NCM460 were maintained in Roswell Park Memorial Institute 1640 (RPMI 1640, Gibco) medium. All cell lines were cultured in Dulbecco's Modified Eagle Medium (DMEM, Gibco) supplemented with 10% Fetal Bovine Serum (FBS, Gibco) and 1% Penicillin-streptomycin (Gibco) at 37 °C in humidified 5% CO_2 incubator (Thermo Scientific).

Measurement of Cl^- influx

HCT116 and RKO were seeded into a 96-well plate (Corning) at a density of 10^4 cells per well and cultured for 14 h. Intracellular Cl^- content was assessed using chloride specific dye *N*-(ethoxycarbonylmethyl)-6-methoxyquinolinium bromide (MQAE, Beyotime). Cells were treated with DMEM containing 5 mM MQAE for 3 h and washed with PBS to remove excess dye. Cells were then treated with compound **L1** and **C2** at various concentrations for 24 h in DMEM medium. The MQAE fluorescence was measured via a Cytation5 imaging reader (BioTek, λ_{ex} = 330 nm and λ_{em} = 465 nm).

MTT assay

Human cancer cell lines (HCT116, RKO, HCT8 and A549) were seeded into a 96-well plate (Corning) at a density of 10^4 cells per well and allowed to grow overnight. Compounds **L1** and **C2** were added to each well at different concentrations by maintaining 0.2% N, N-dimethylformamide (DMF, Sigma-Aldrich) and incubated for 24 h. Then, cells were treated with 0.5 mg/mL MTT (Sigma-Aldrich) in DMEM medium and incubated in the dark for 4 h. Supernatant was removed and the precipitated formazan crystals were then dissolved with 100

μL dimethyl sulfoxide (DMSO, Sigma-Aldrich). Absorbance was measured at 560 nm using a Cytation5 imaging reader.[11]

Chloride mediated cell death analysis

Hank's balanced salt solution (HBSS with Cl^-, pH 7.4) was prepared by mixing 136.9 mM NaCl, 5.5 mM KCl, 0.34 mM Na_2HPO_4, 0.44 mM KH_2PO_4, 0.81 mM $MgSO_4$, 1.25 mM $CaCl_2$, 5.5 mM D-glucose, 4.2 mM $NaHCO_3$ and 10 mM HEPES. Hank's balanced salt solution (HBSS without Cl^-, pH 7.4) was prepared by mixing 136.9 mM Na-gluconate, 5.5 mM K-gluconate, 0.34 mM Na_2HPO_4, 0.44 mM KH_2PO_4, 0.81 mM $MgSO_4$, 1.25 mM Ca-gluconate, 5.5 mM D-glucose, 4.2 mM $NaHCO_3$ and 10 mM HEPES. Both HBSS solutions were used as cell culture medium by adding 20% dialyzed FBS (dFBS, Biological Industries) and 1% Penicillin-streptomycin (Gibco). HCT116 and RKO cells were seeded into a 96-well plate (Corning) at a density of 2×10^4 cells per well and cultured for 14 h. Then, cells were treated with compounds **L1** and **C2** in the two distinct HBSS buffers for 72 h. Cell viability was determined by MTT assay. HEK293 and NCM460 were treated with compound **C2** in Cl^- containing HBSS medium for 24 h. Cytotoxicity was determined by MTT assay with a Cytation5 imaging reader (BioTek).

Measurement of mitochondrial membrane potential

HCT116 cells were seeded into 35 mm dishes (glass bottom) at a density of 8×10^4 and cultured overnight. Cells were treated with compound **L1** and **C2** at different concentrations for 24 h and subsequently incubated with JC-1 dye (1 $\mu g\cdot mL^{-1}$) at 37 °C for 30 min. After washing with PBS, cell morphology was observed under a fluorescence microscope in both red and green channel. Image J software was used to analyze the ratio of pixel intensity (red/green) of each image.

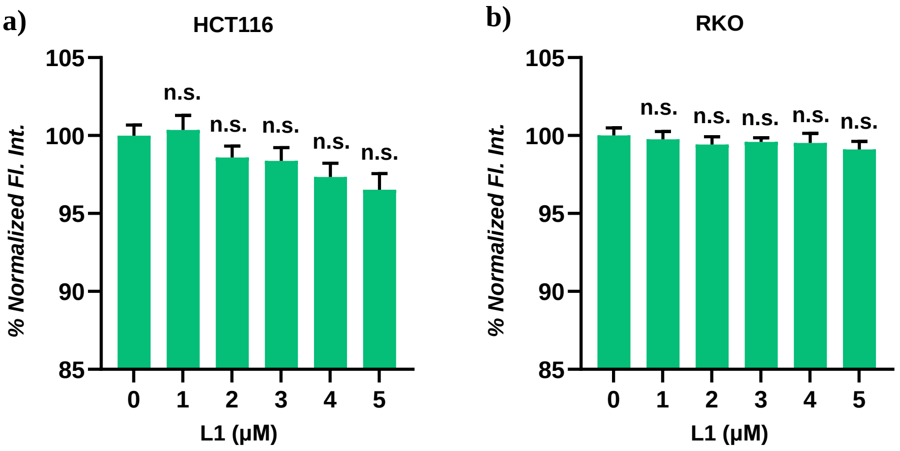

Figure S15. a) Normalized fluorescence intensity of HCT116 cells and b) RKO cells incubated with MQAE (5 mM) for 3 h followed by treatment of compound **L1** (0-5 μL) for 24 h. All bars in graph represent mean ± SEM. One-way ANOVA was performed (n = 3). *P<0.05; **P<0.01; ***P<0.001; n.s., not significant.

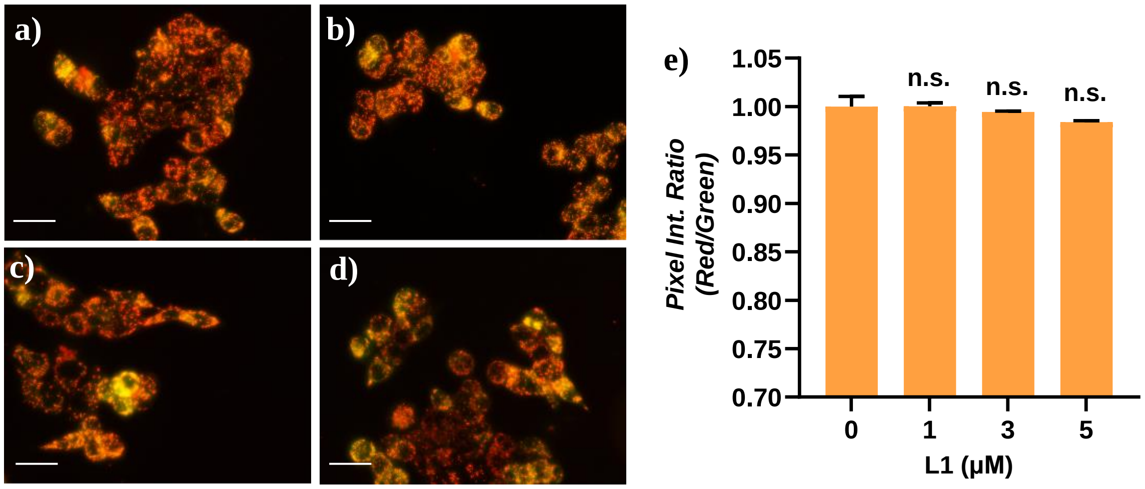

Figure S16. Cell imaging of HCT116 cells incubated with a) 0 μM, b) 1 μM, c) 3 μM, d) 5 μM of compound **L1** for 24 h followed by staining with JC-1. e) The pixel ratio (red/green) for each set of cells was plotted in the bar graph. *P<0.05; **P<0.01; ***P<0.001; n.s., not significant.

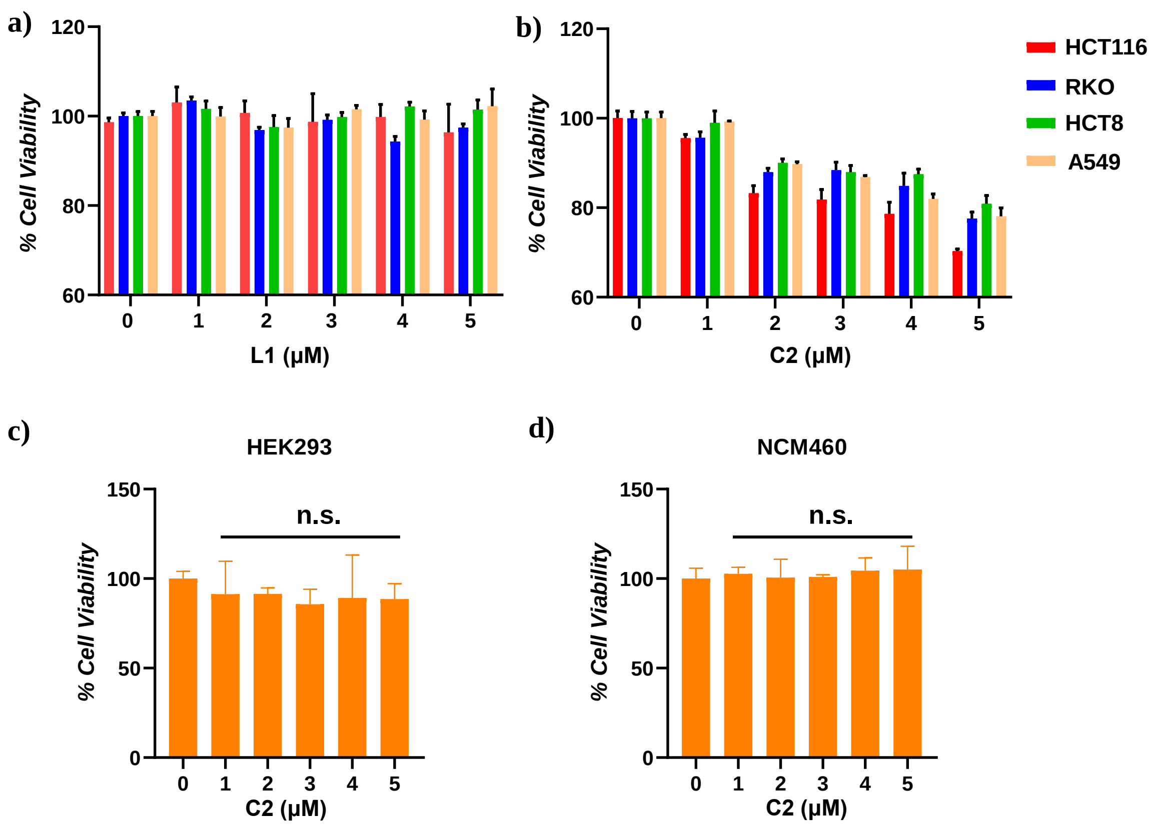

Figure S17. Cell viability obtained from MTT assay upon dose dependent treatment of a) **L1** and b) **C2** for 24 h in various cell lines. Cytotoxicity of compound **C2** was assessed by MTT method. HEK293 cells c) and NCM460 cells d) were cultured in HBSS solution (with Cl^-) upon treatment of compound **C2** (0, 1, 2, 3, 4, 5 μM) for 24 h. All bars in graph represent mean ± SEM. One-way ANOVA was performed (n=3). *P<0.05; **P<0.01; ***P<0.001, n.s., not significant.

13. The ¹H NMR, ¹³C NMR, COSY NMR, DOSY NMR, and ESI-MS spectra of ligands and metallacages

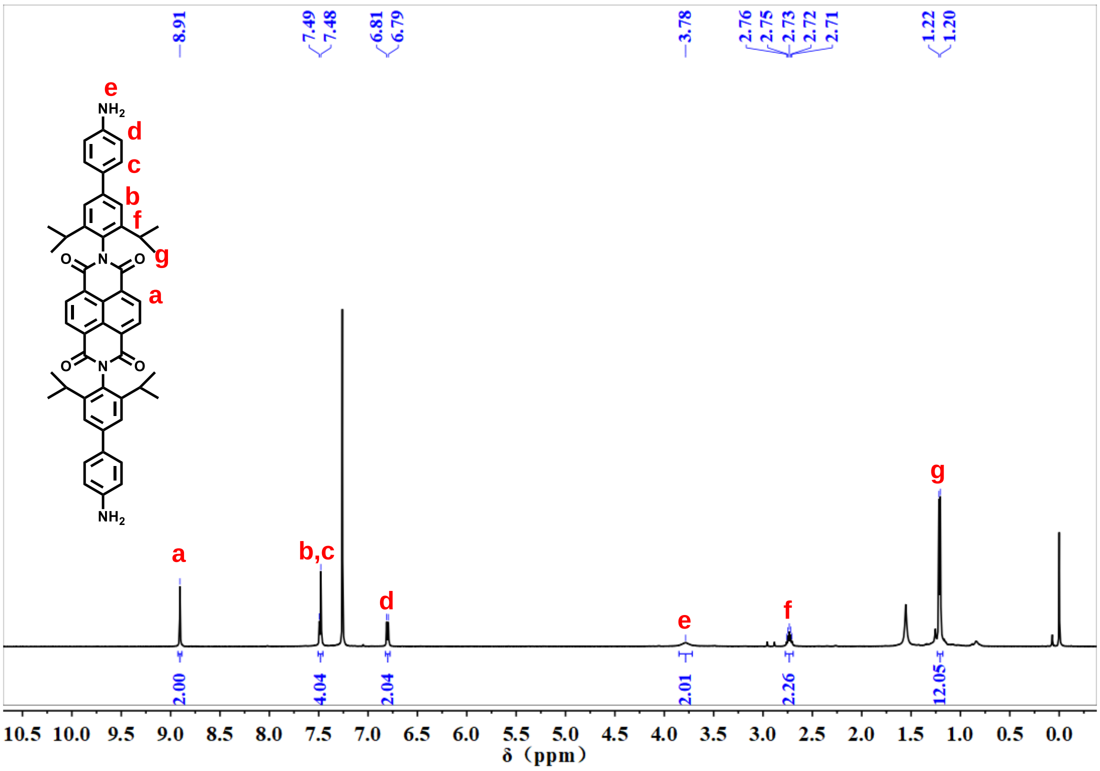

Figure S18. ¹H NMR (500 MHz, $CDCl_3$, 298 K) spectrum of **3**.

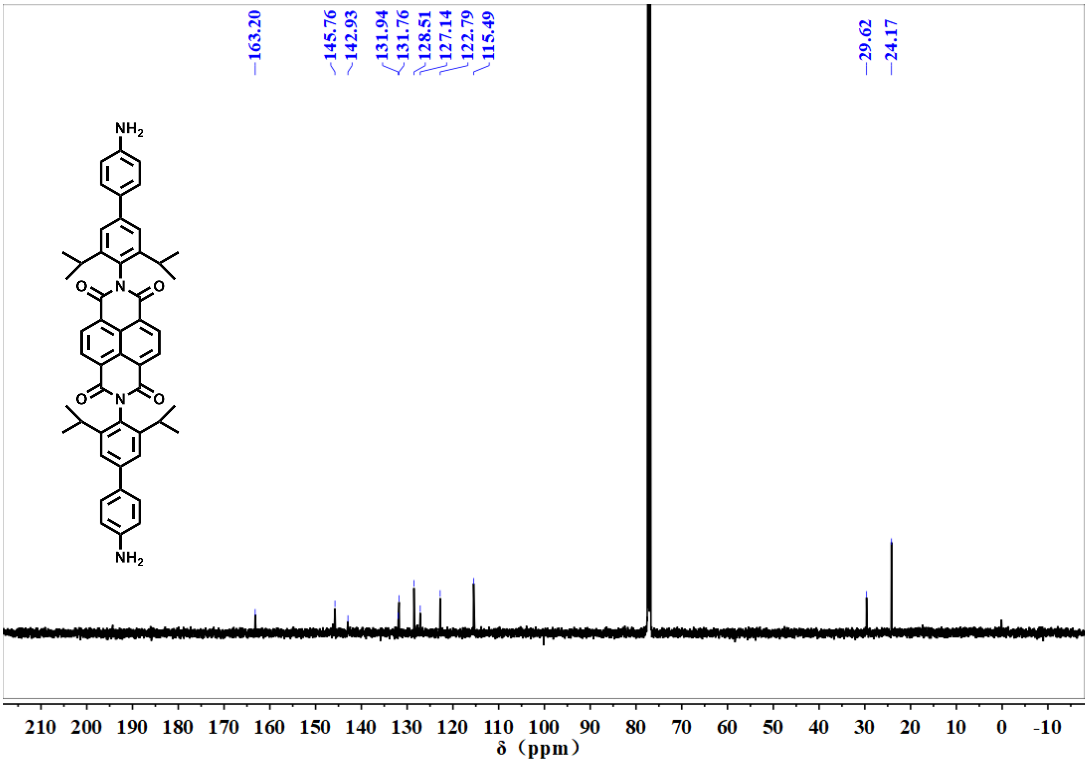

Figure S19. ¹³C NMR (126 MHz, $CDCl_3$, 298 K) spectrum of **3**.

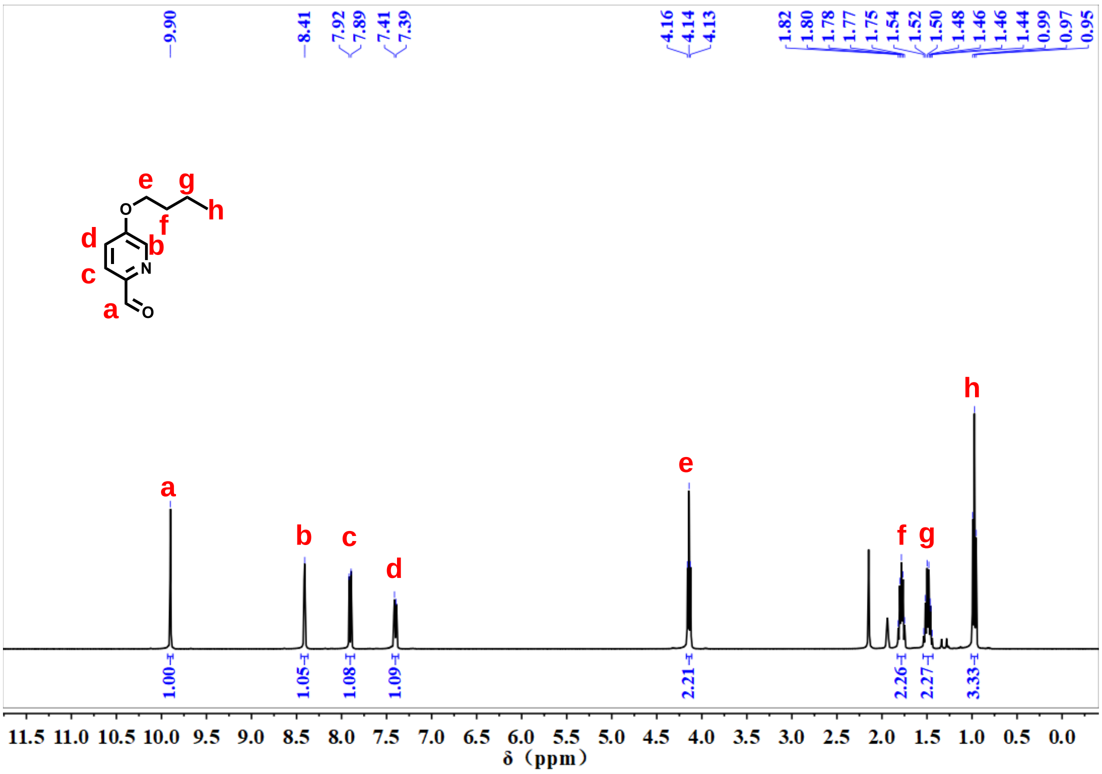

Figure S20. ^{1}H NMR (400 MHz, CD_3CN, 298 K) spectrum of **4**.

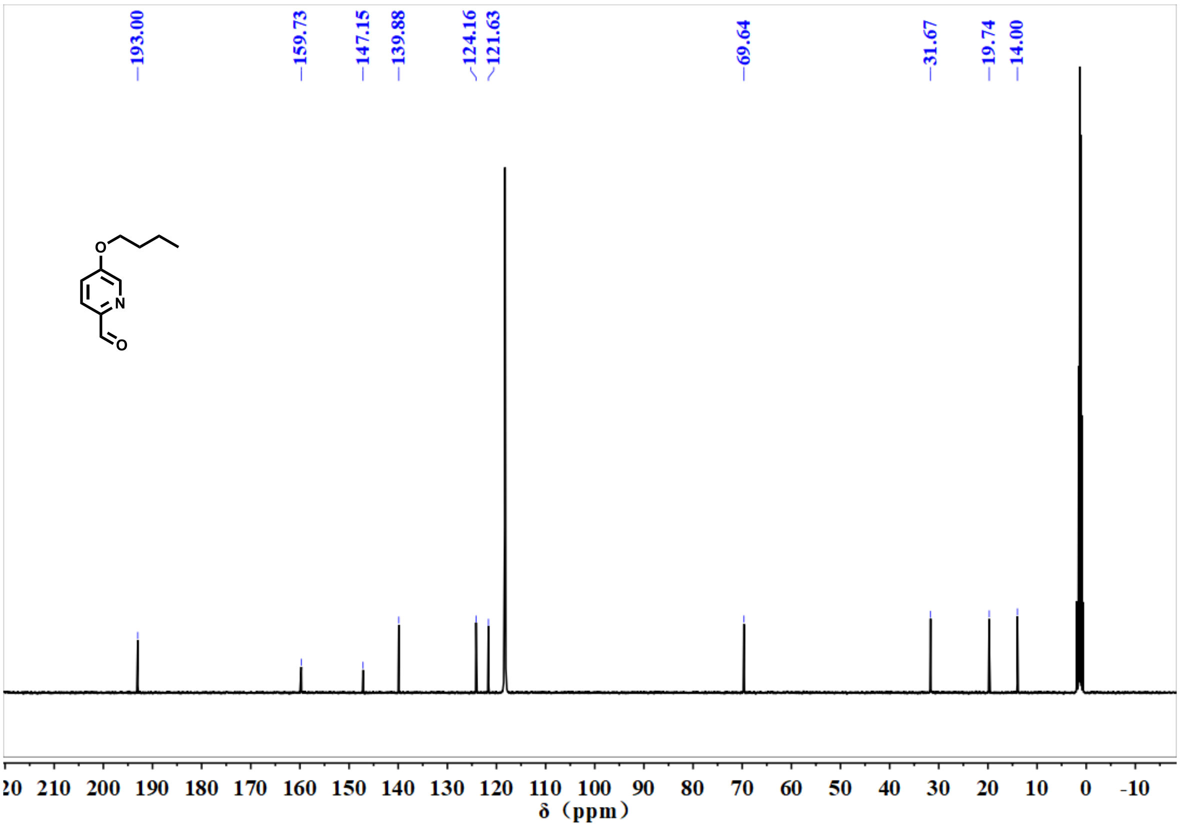

Figure S21. ^{13}C NMR (101 MHz, CD_3CN, 298 K) spectrum of **4**.

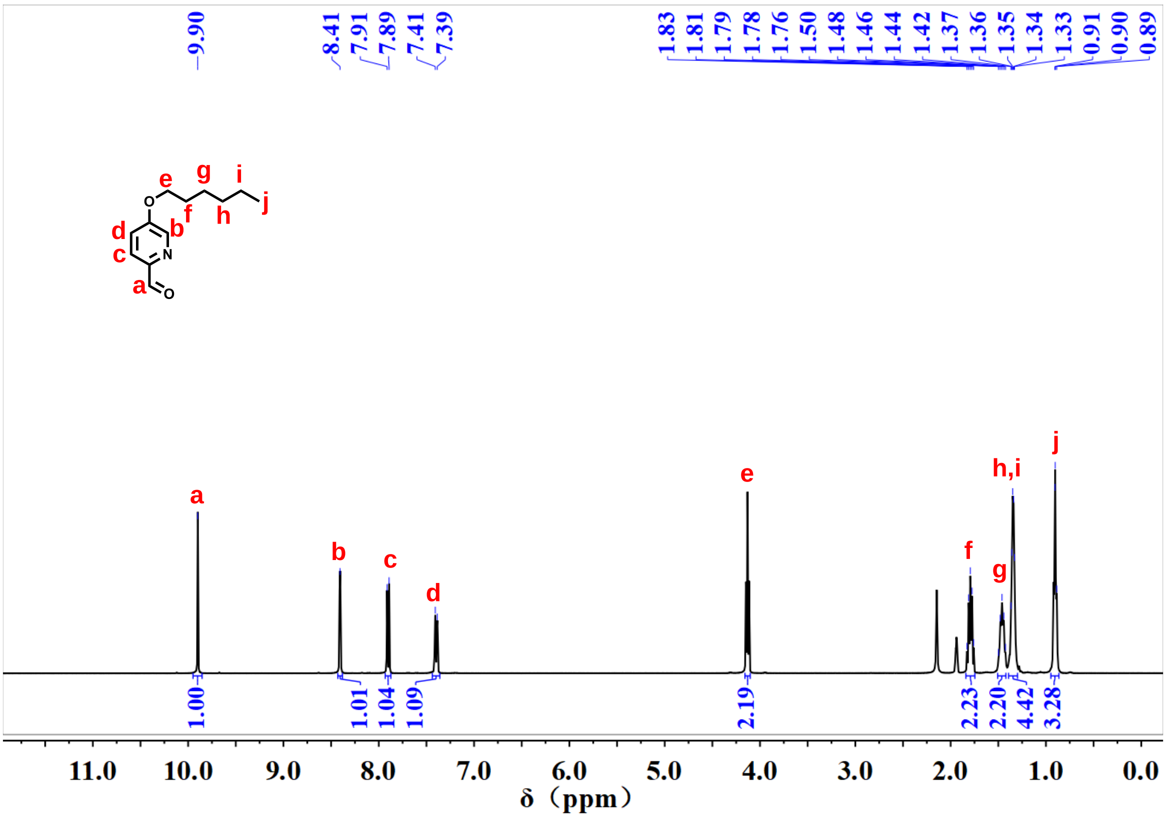

Figure S22. ^{1}H NMR (400 MHz, CD_3CN, 298 K) spectrum of **5**.

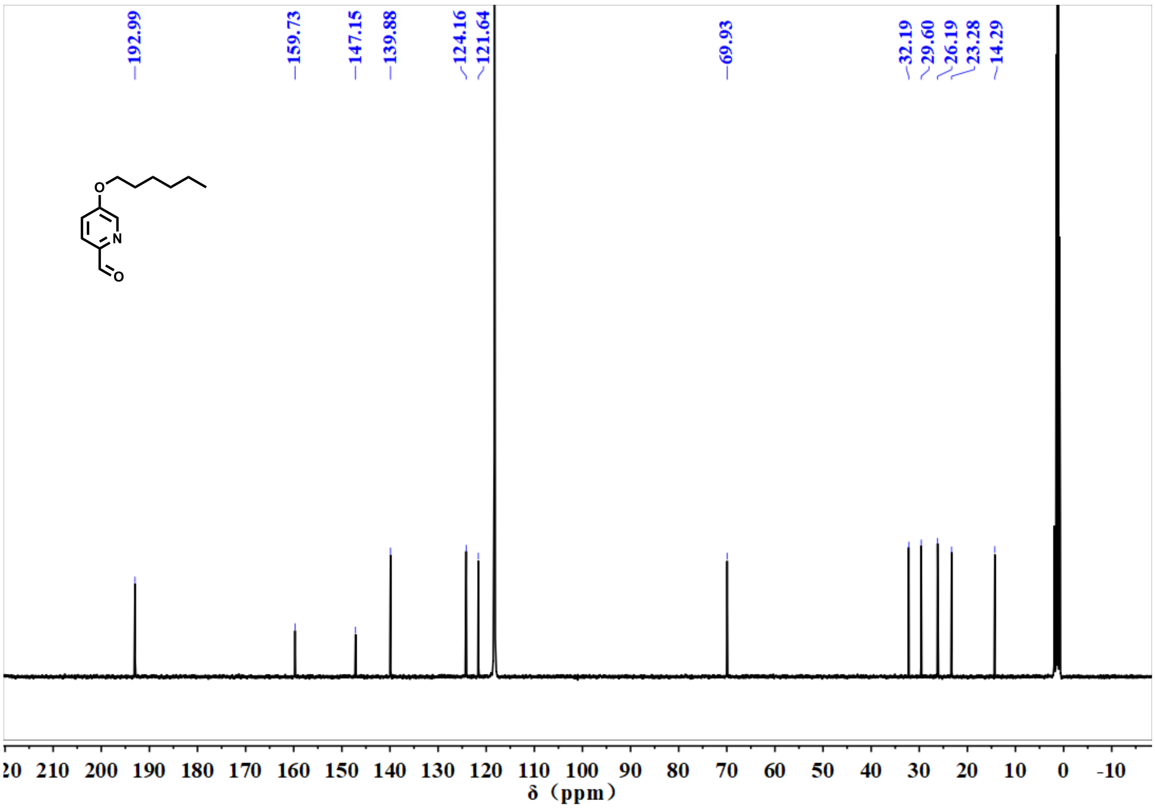

Figure S23. ^{13}C NMR (101 MHz, CD_3CN, 298 K) spectrum of **5**.

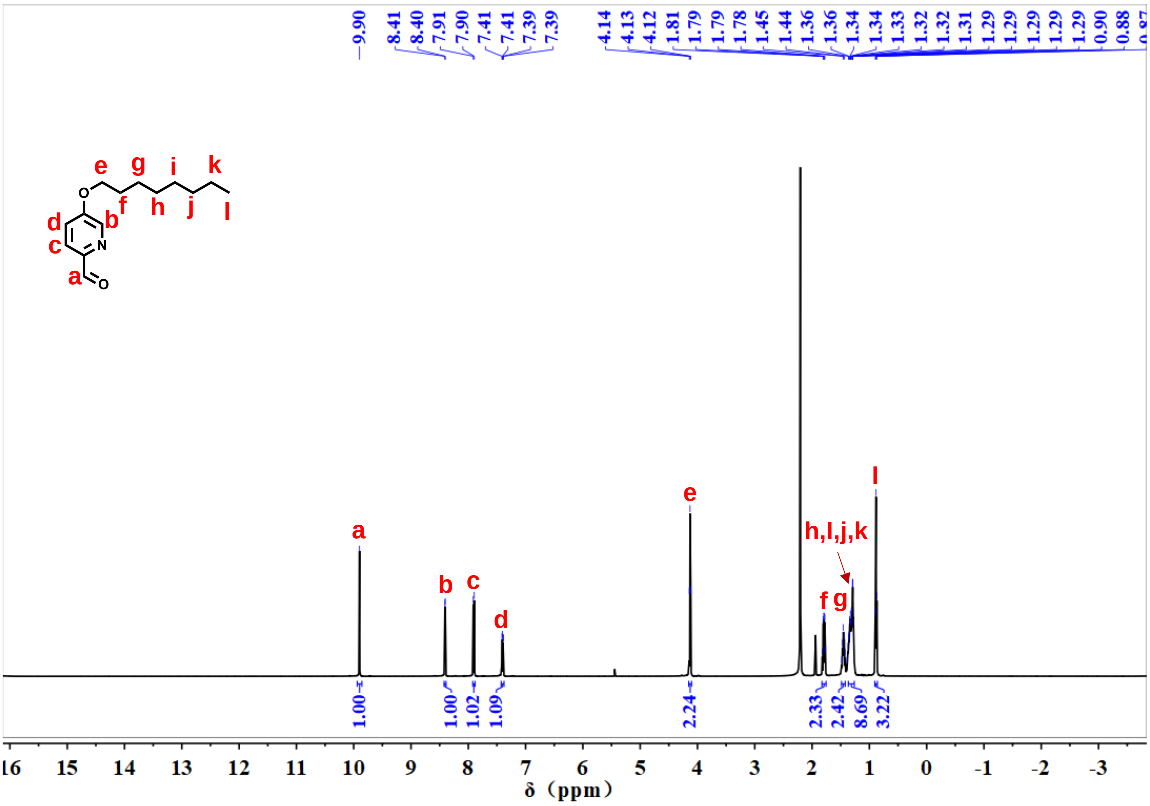

Figure S24. ^{1}H NMR (500 MHz, CD_3CN, 298 K) spectrum of **6**.

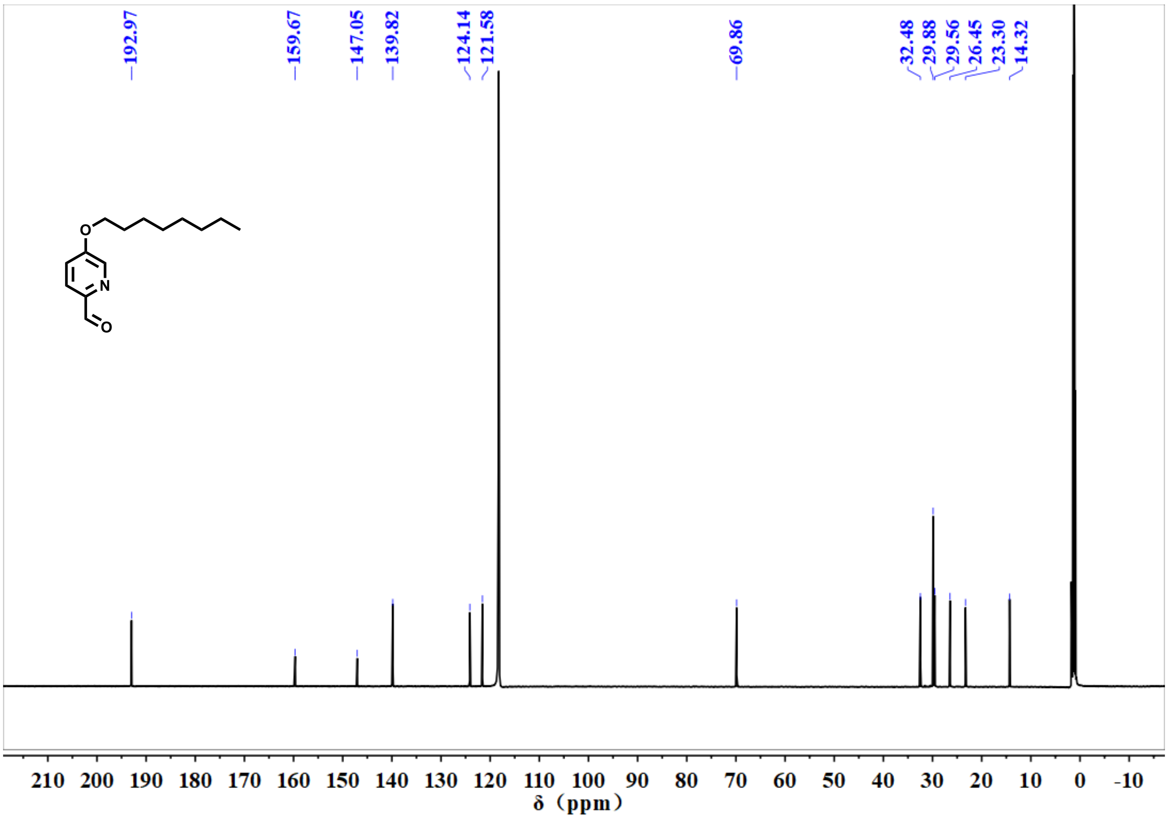

Figure S25. ^{13}C NMR (126 MHz, CD_3CN, 298 K) spectrum of **6**.

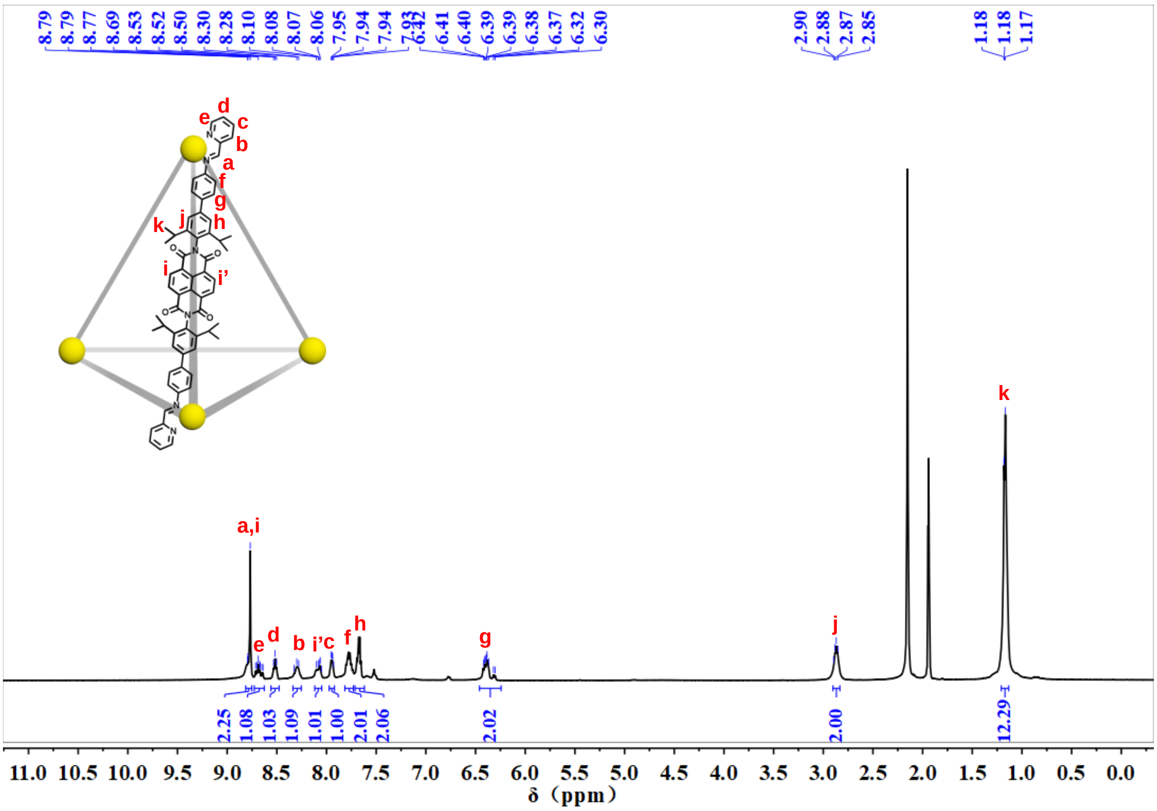

Figure S26. ^{1}H NMR (500 MHz, CD_3CN, 298 K) spectrum of **C1**.

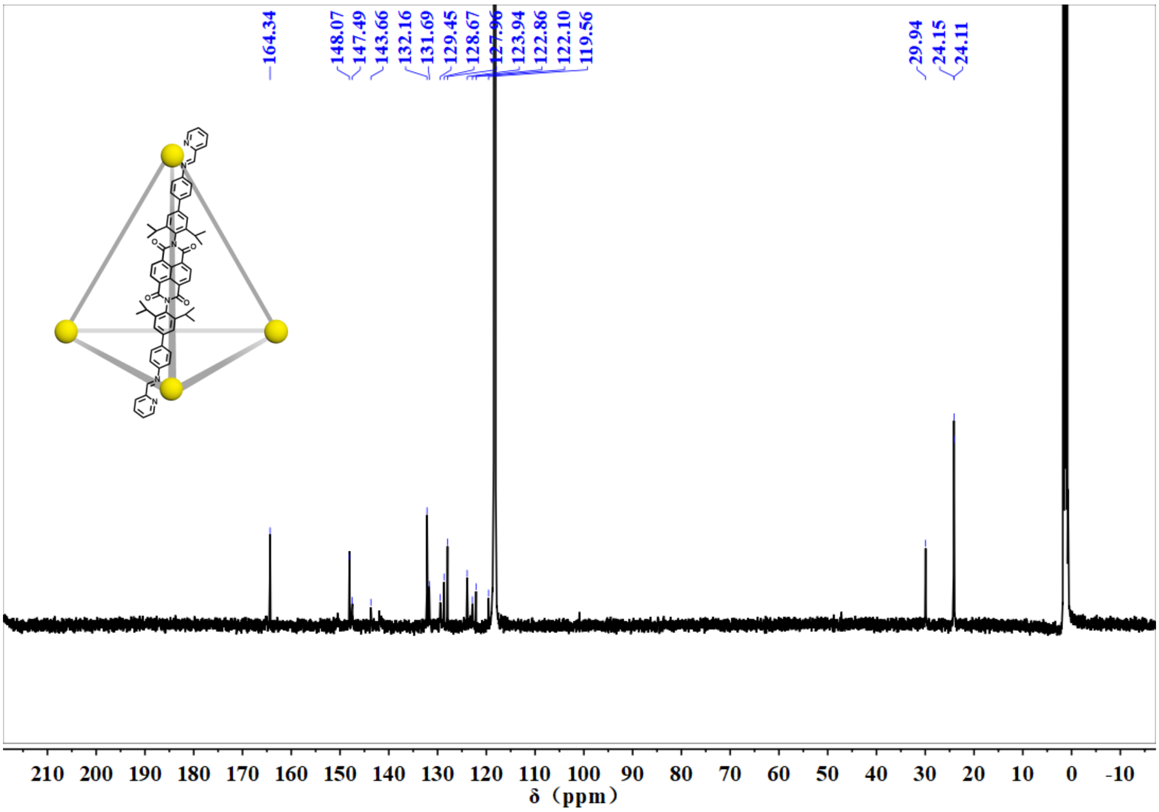

Figure S27. ^{13}C NMR (126 MHz, CD_3CN, 298 K) spectrum of **C1**.

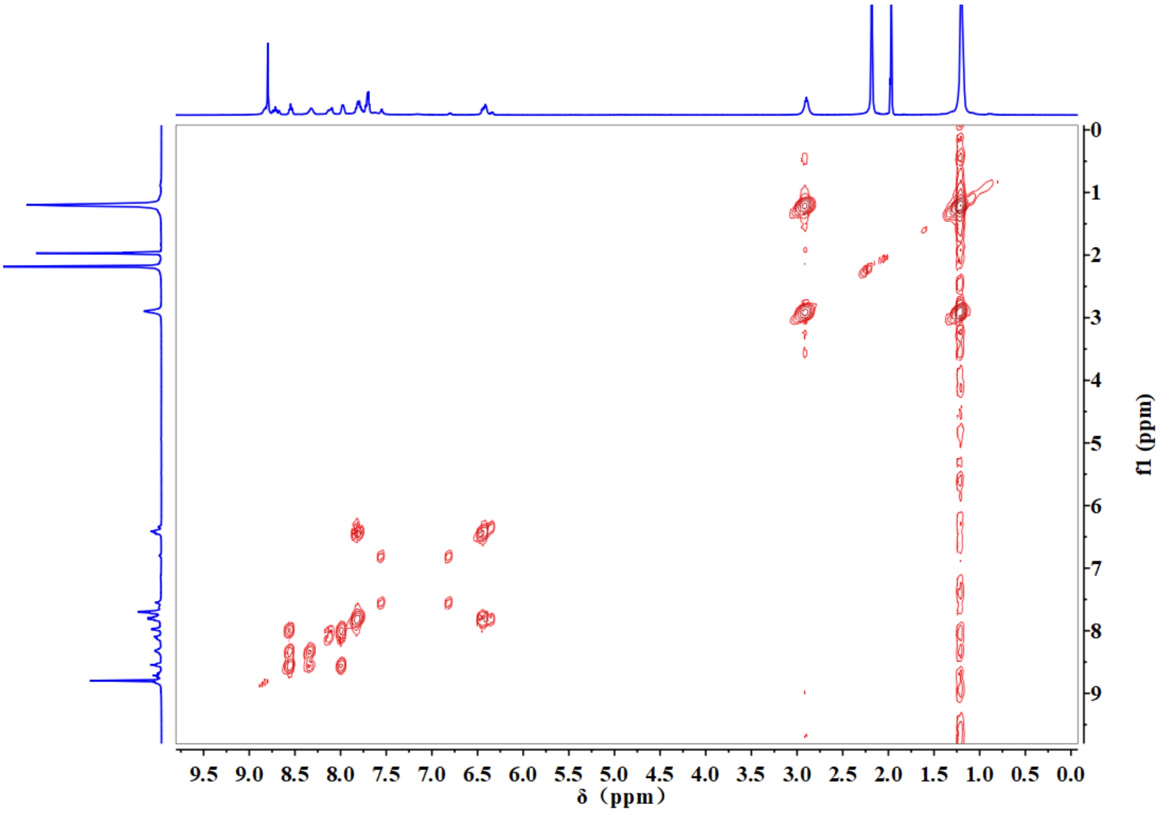

Figure S28. 2D COSY NMR (500 MHz, CD_3CN, 298 K) spectrum of **C1**.

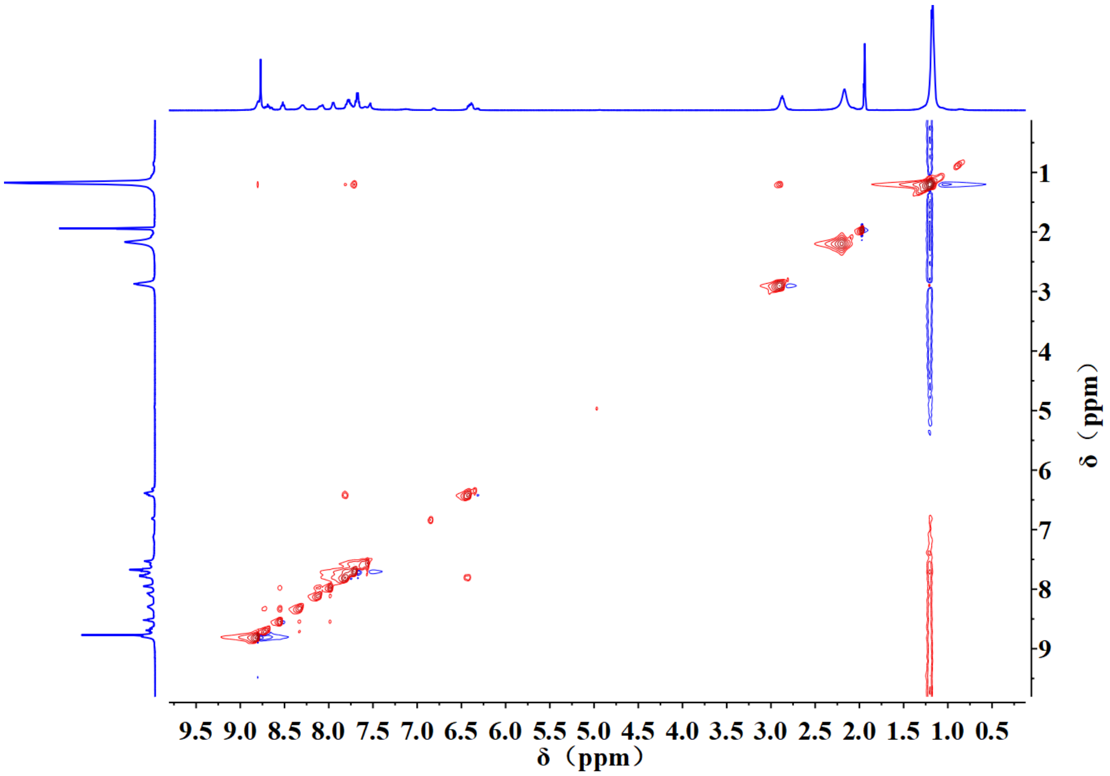

Figure S29. 2D NOESY NMR (500 MHz, CD_3CN, 298 K) spectrum of **C1**.

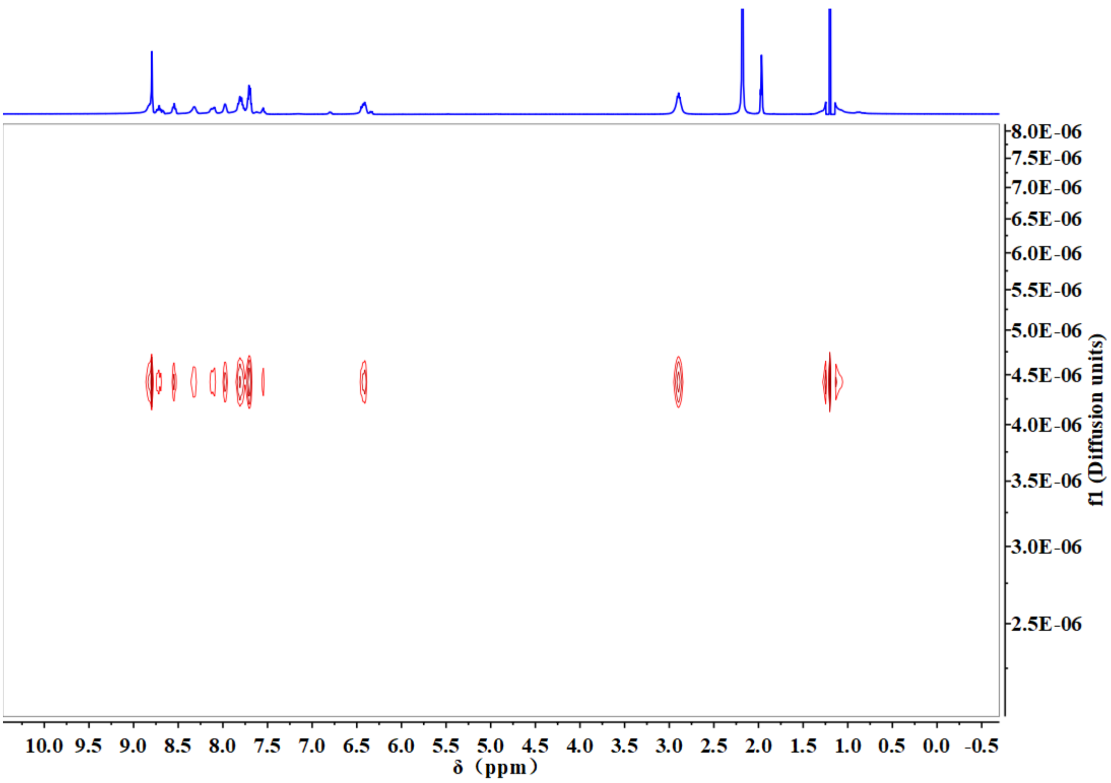

Figure S30. 2D DOSY NMR (500 MHz, CD_3CN, 298 K) spectrum of **C1**.

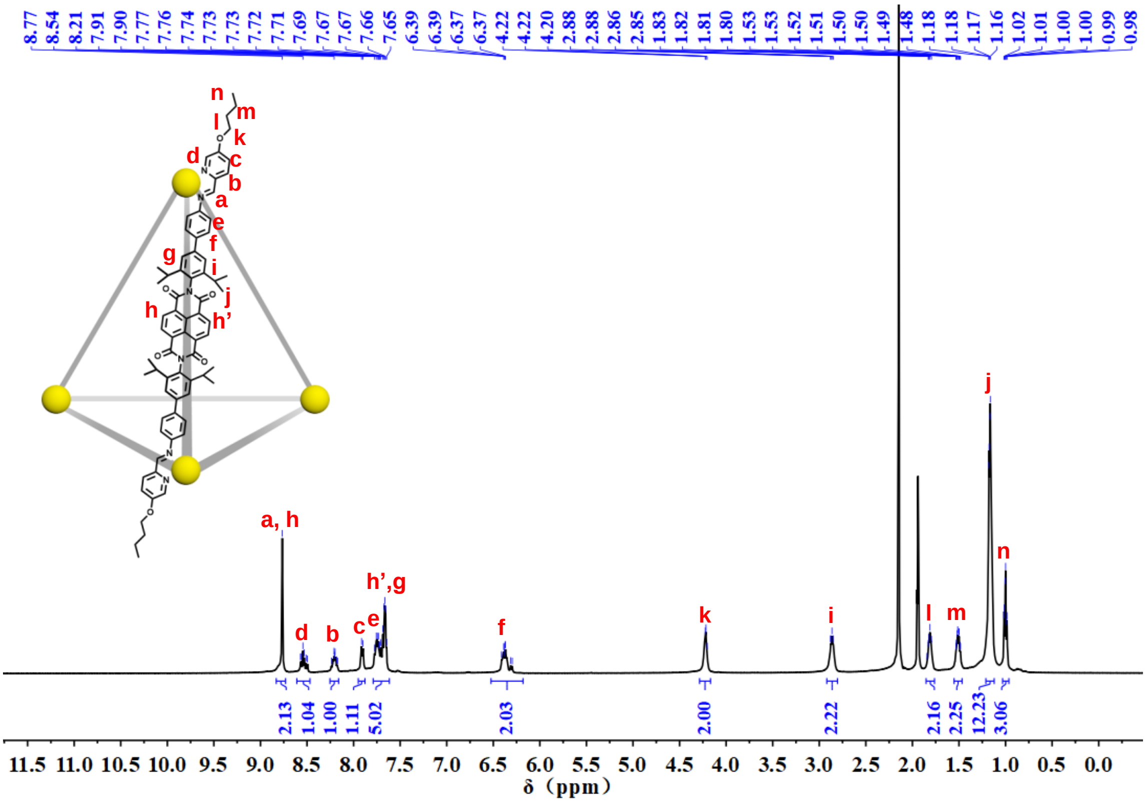

Figure S31. ^{1}H NMR (500 MHz, CD_3CN, 298 K) spectrum of **C2**.

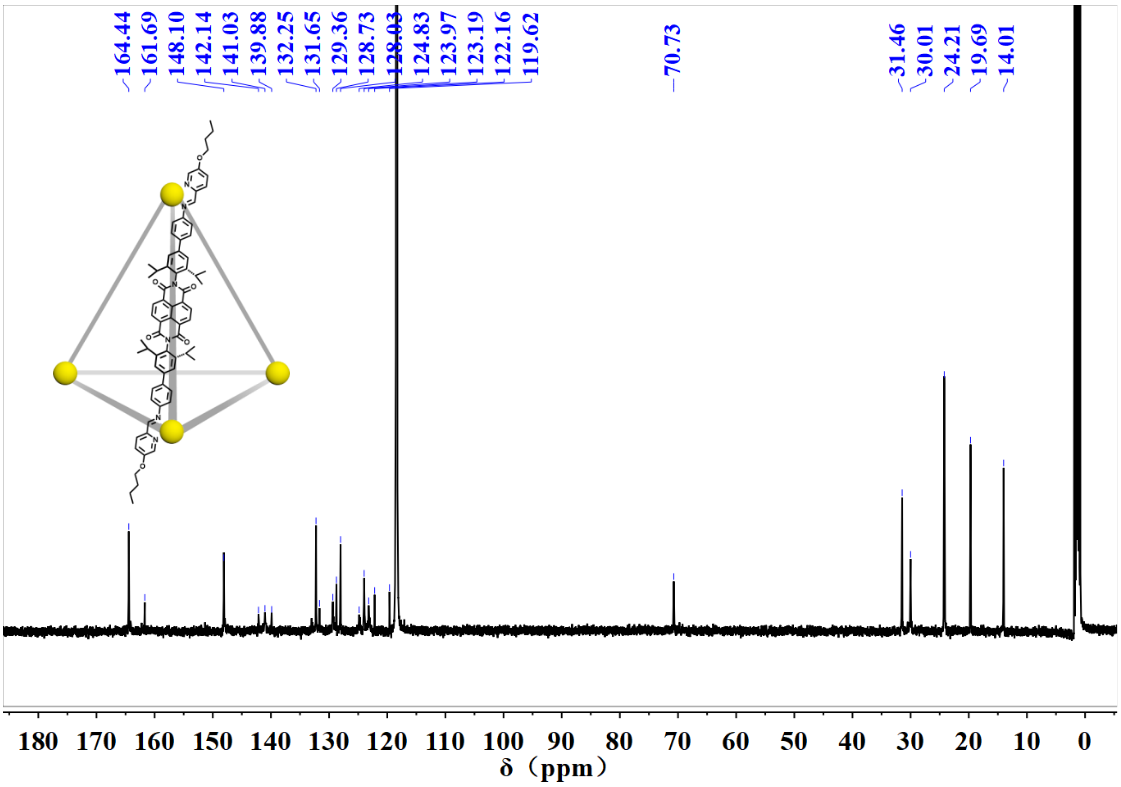

Figure S32. ^{13}C NMR (126 MHz, CD_3CN, 298 K) spectrum of **C2**.

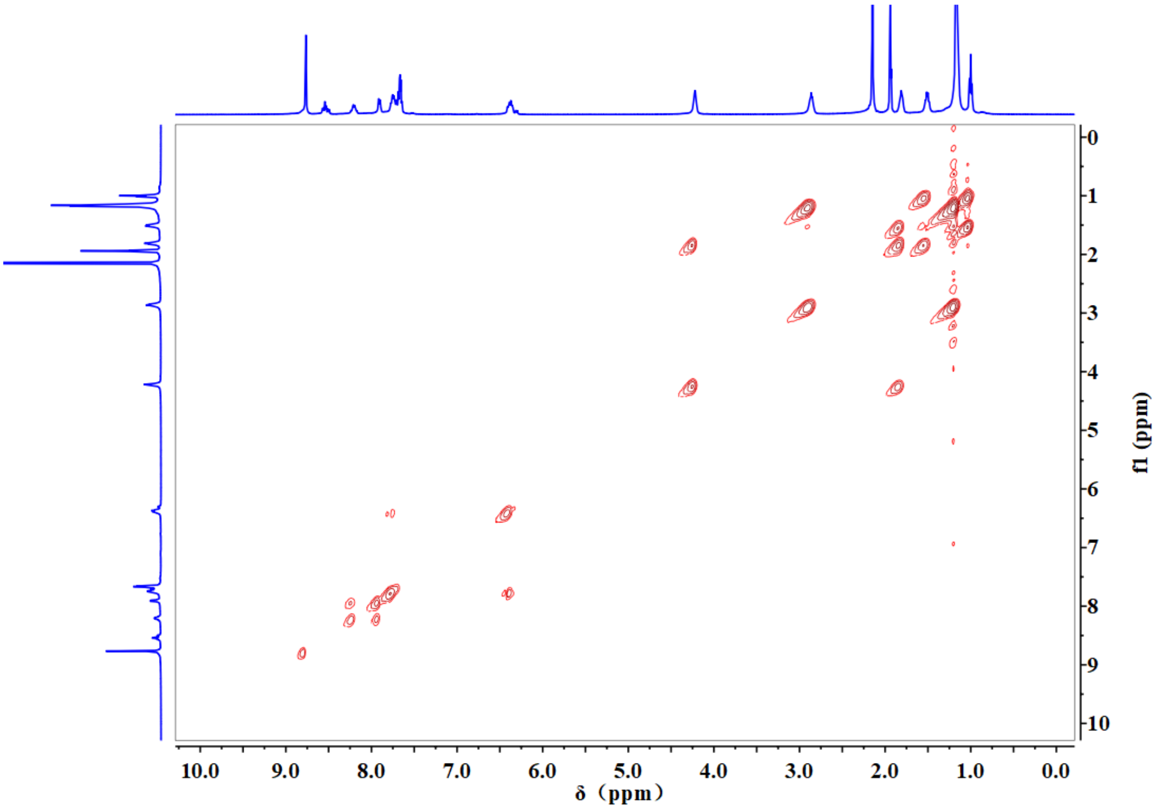

Figure S33. 2D COSY NMR (500 MHz, CD_3CN, 298 K) spectrum of **C2**.

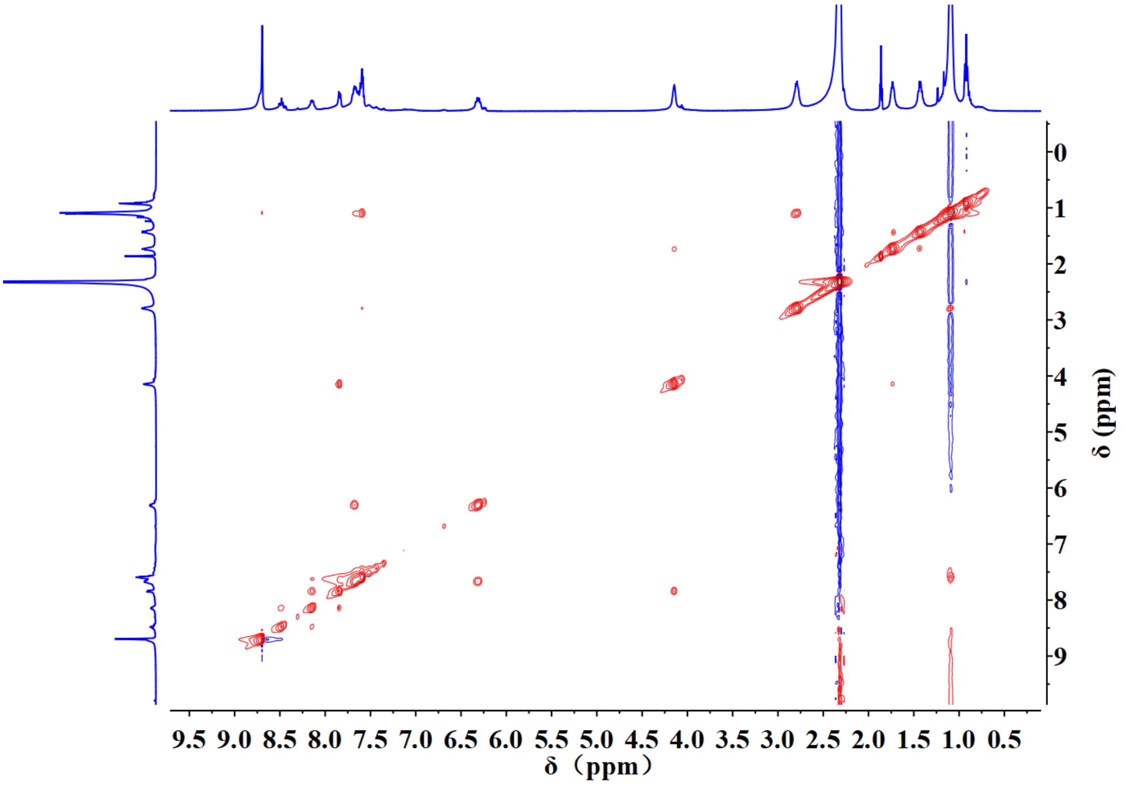

Figure S34. 2D NOESY NMR (500 MHz, CD_3CN, 298 K) spectrum of **C2**.

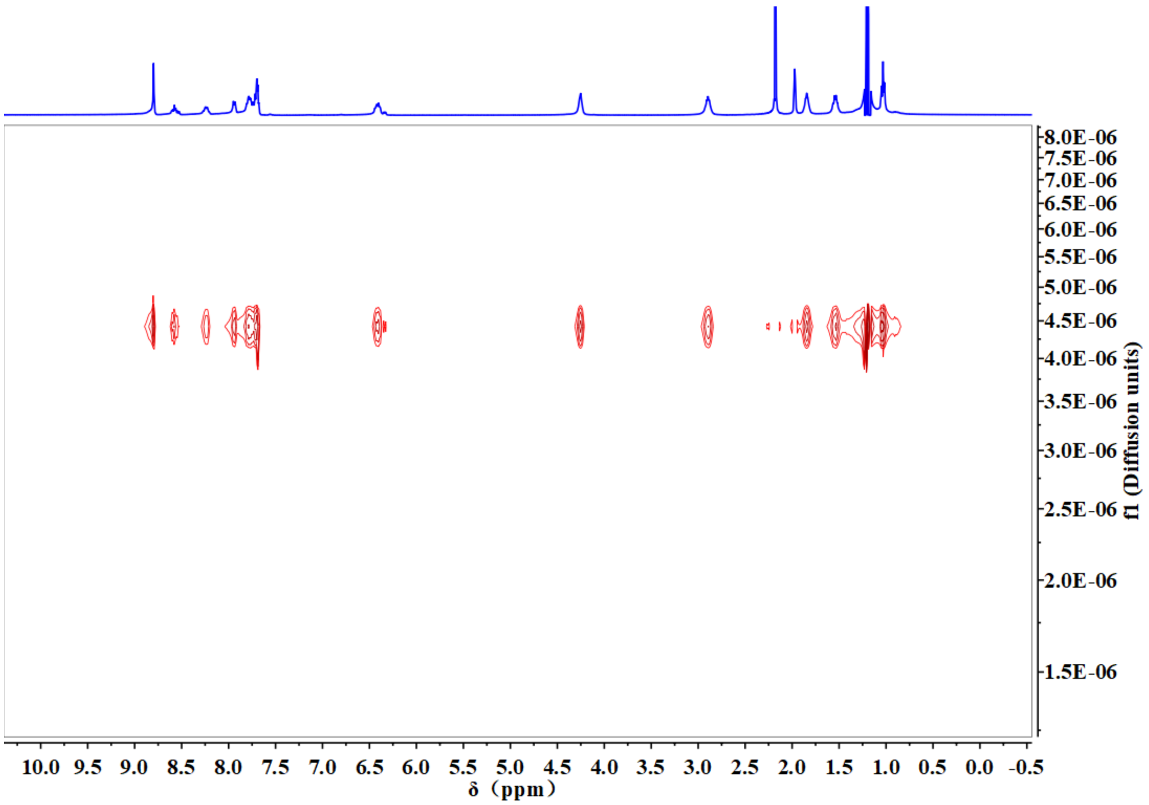

Figure S35. 2D DOSY NMR (500 MHz, CD_3CN, 298 K) spectrum of **C2**.

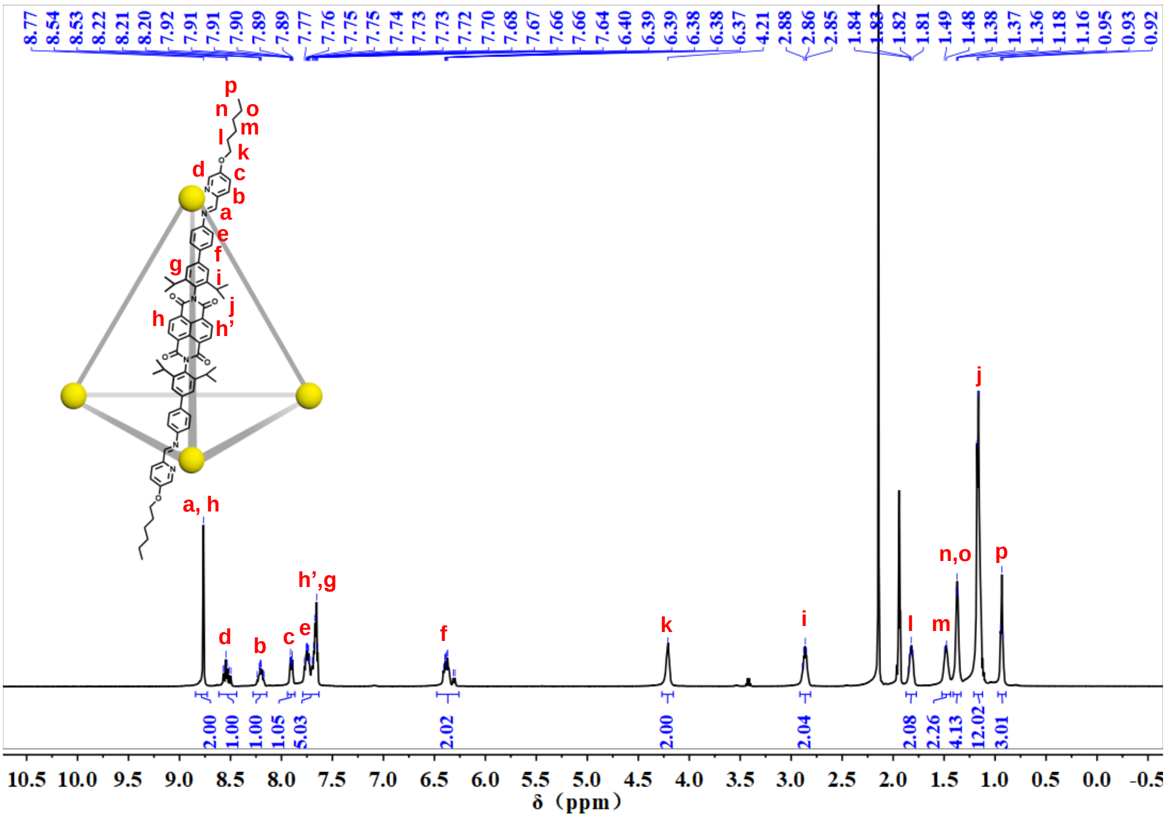

Figure S36. ^{1}H NMR (500 MHz, CD_3CN, 298 K) spectrum of **C3**.

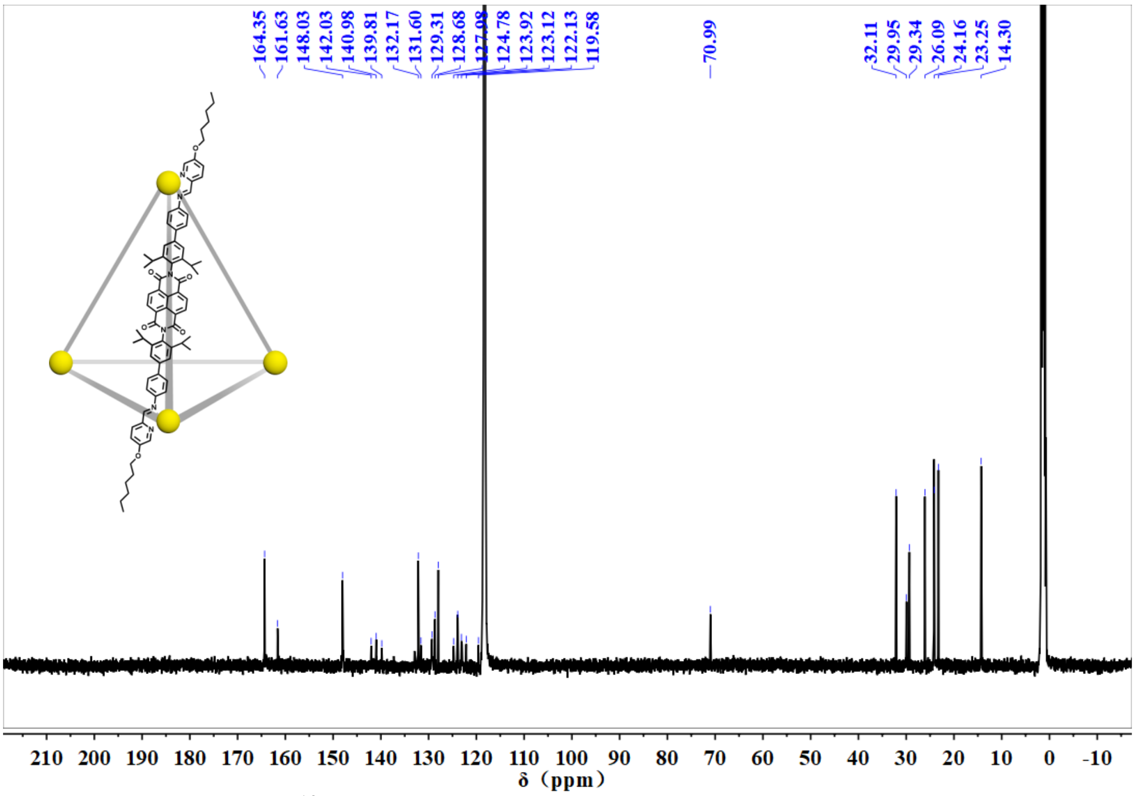

Figure S37. ^{13}C NMR (126 MHz, CD_3CN, 298 K) spectrum of **C3**.

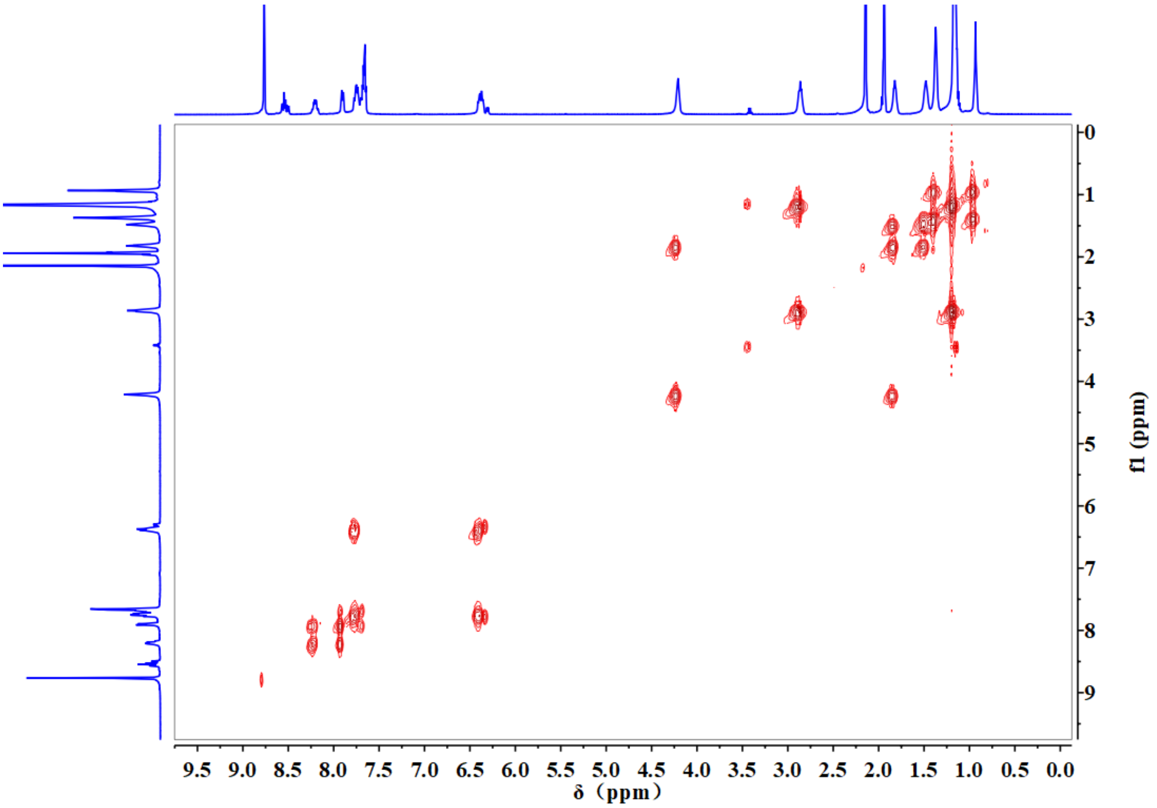

Figure S38. 2D COSY NMR (500 MHz, CD_3CN, 298 K) spectrum of **C3**.

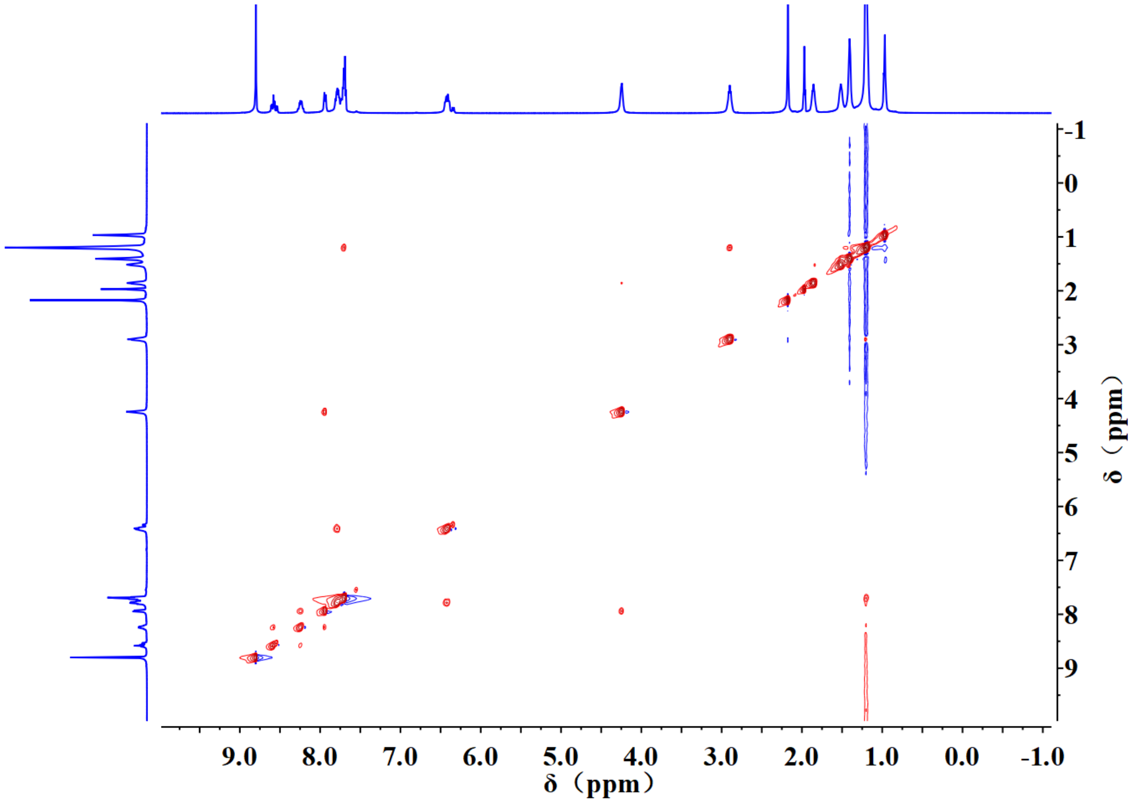

Figure S39. 2D NOESY NMR (500 MHz, CD_3CN, 298 K) spectrum of **C3**.

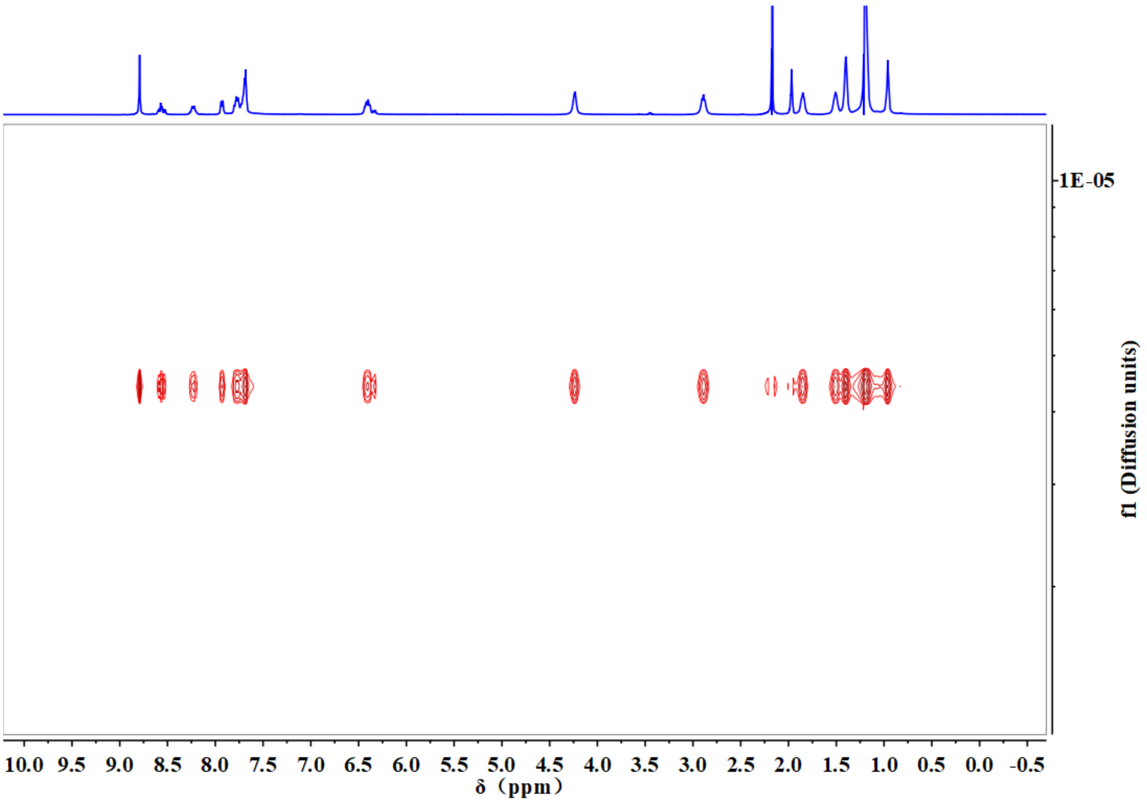

Figure S40. 2D DOSY NMR (500 MHz, CD_3CN, 298 K) spectrum of **C3**.

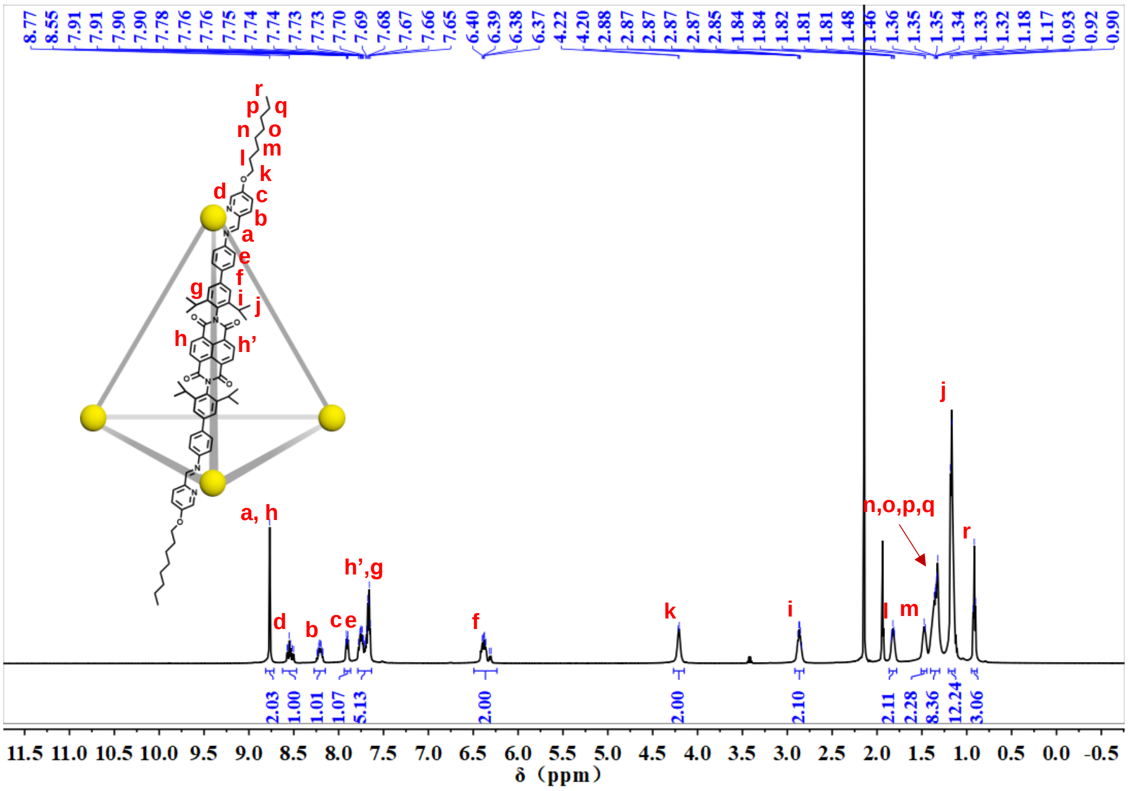

Figure S41. ^{1}H NMR (500 MHz, CD_3CN, 298 K) spectrum of **C4**.

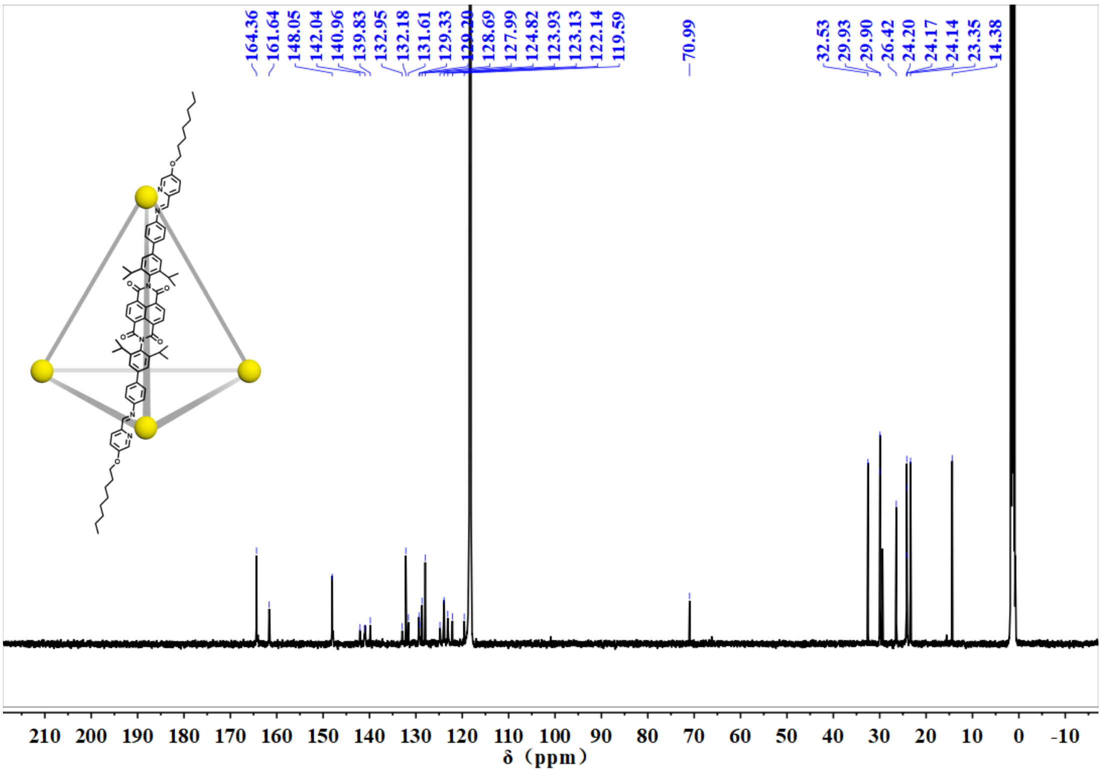

Figure S42. ^{13}C NMR (126 MHz, CD_3CN, 298 K) spectrum of **C4**.

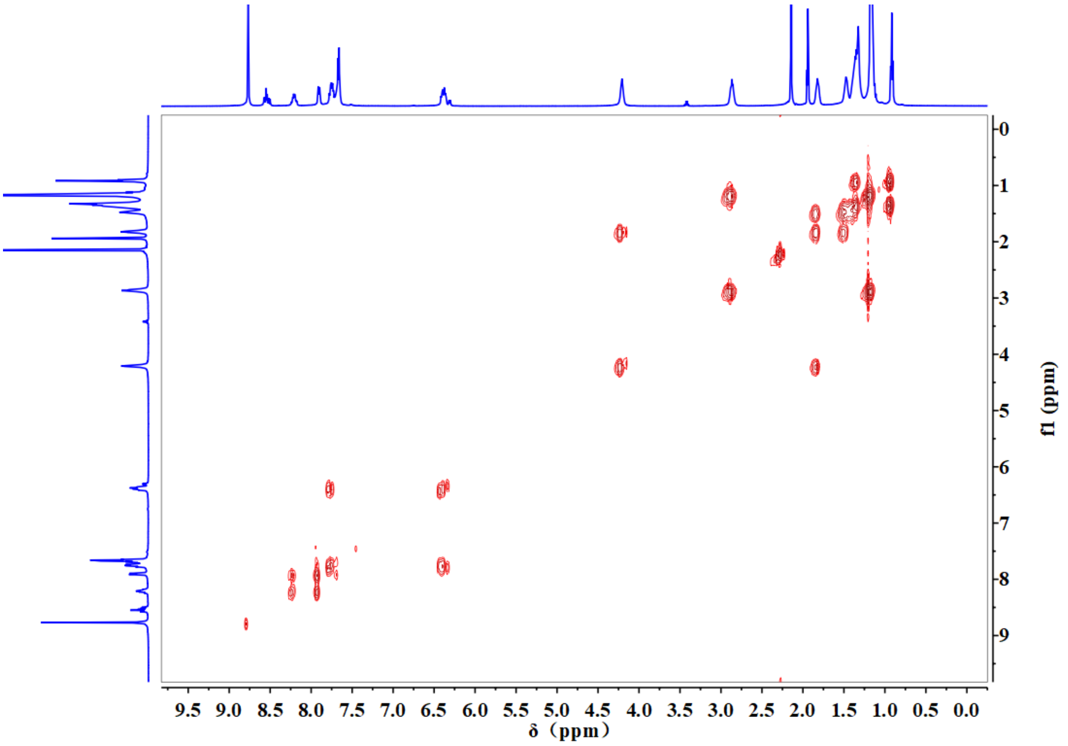

Figure S43. 2D COSY NMR (500 MHz, CD_3CN, 298 K) spectrum of **C4**.

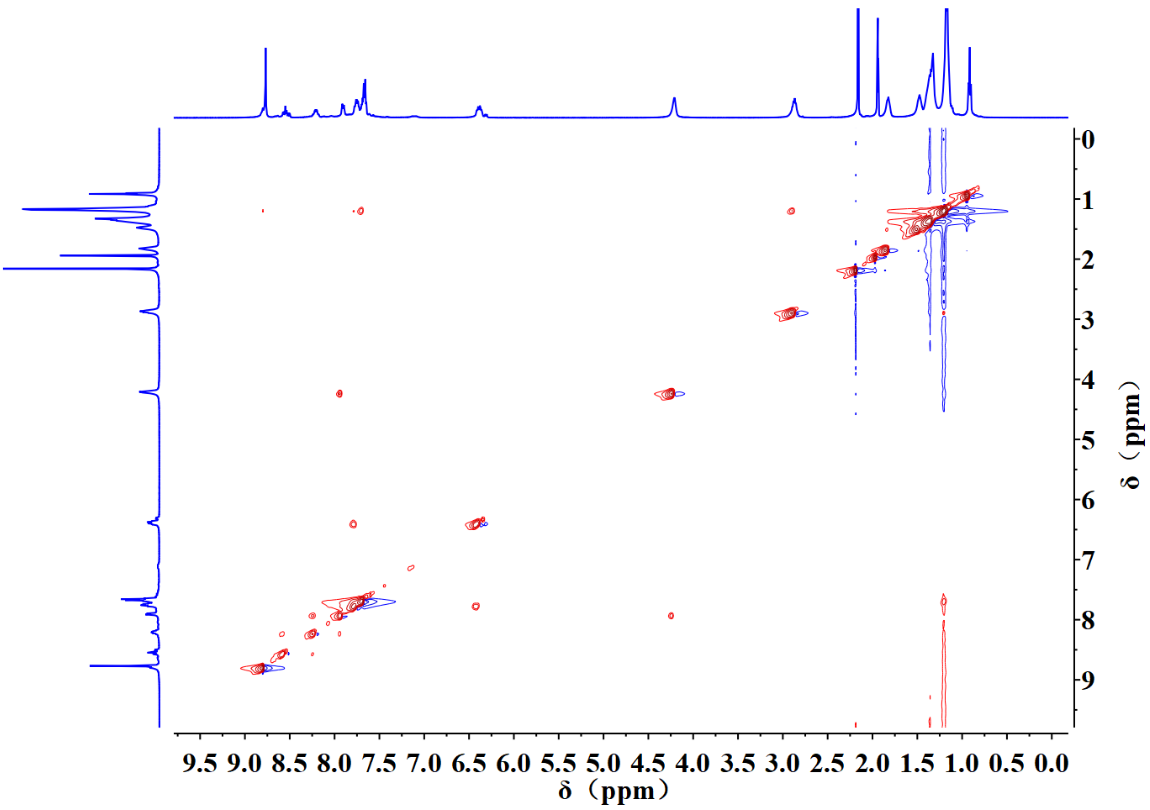

Figure S44. 2D NOESY NMR (500 MHz, CD_3CN, 298 K) spectrum of **C4**.

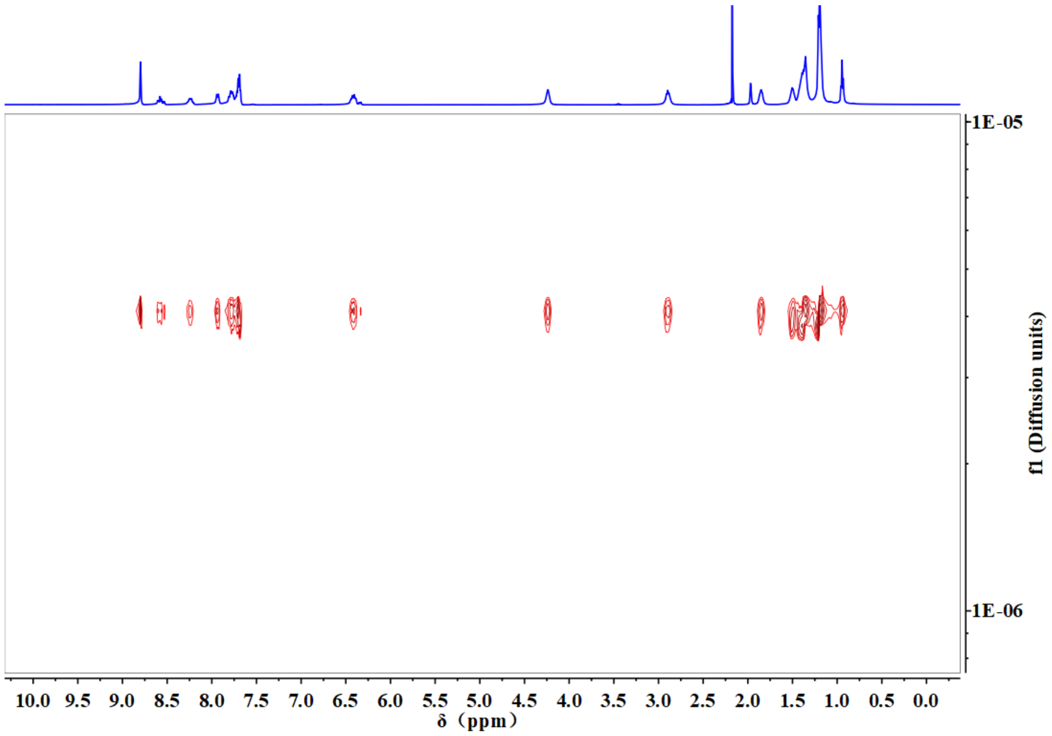

Figure S45. 2D DOSY NMR (500 MHz, CD_3CN, 298 K) spectrum of **C4**.

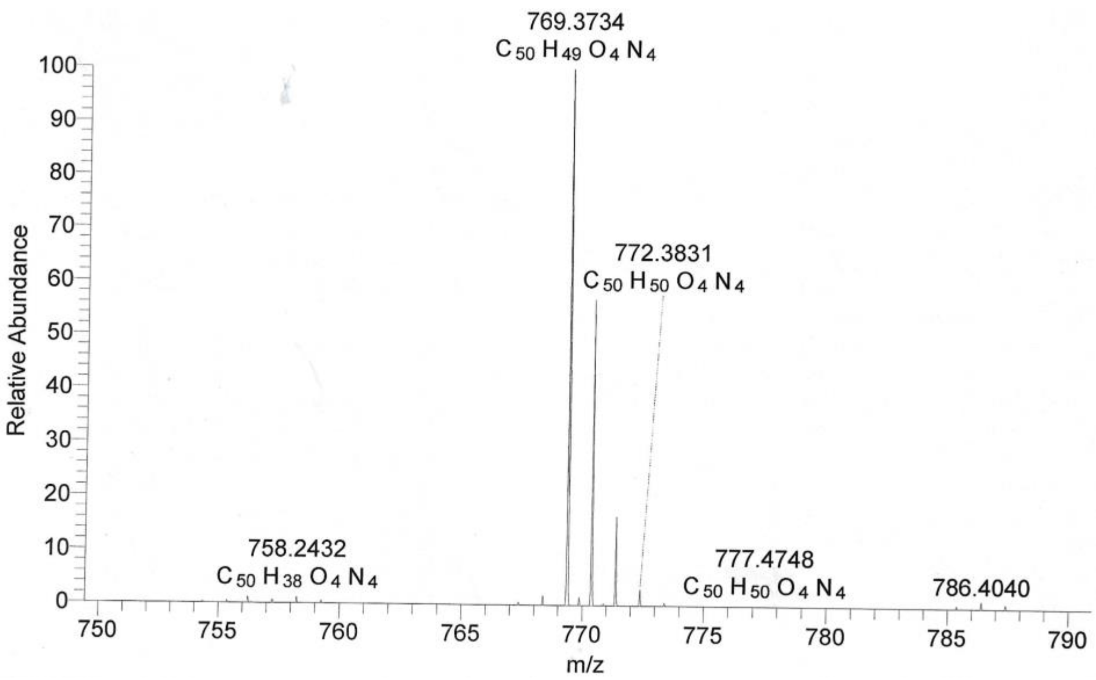

Figure S46. ESI-MS spectrum of **L1.**

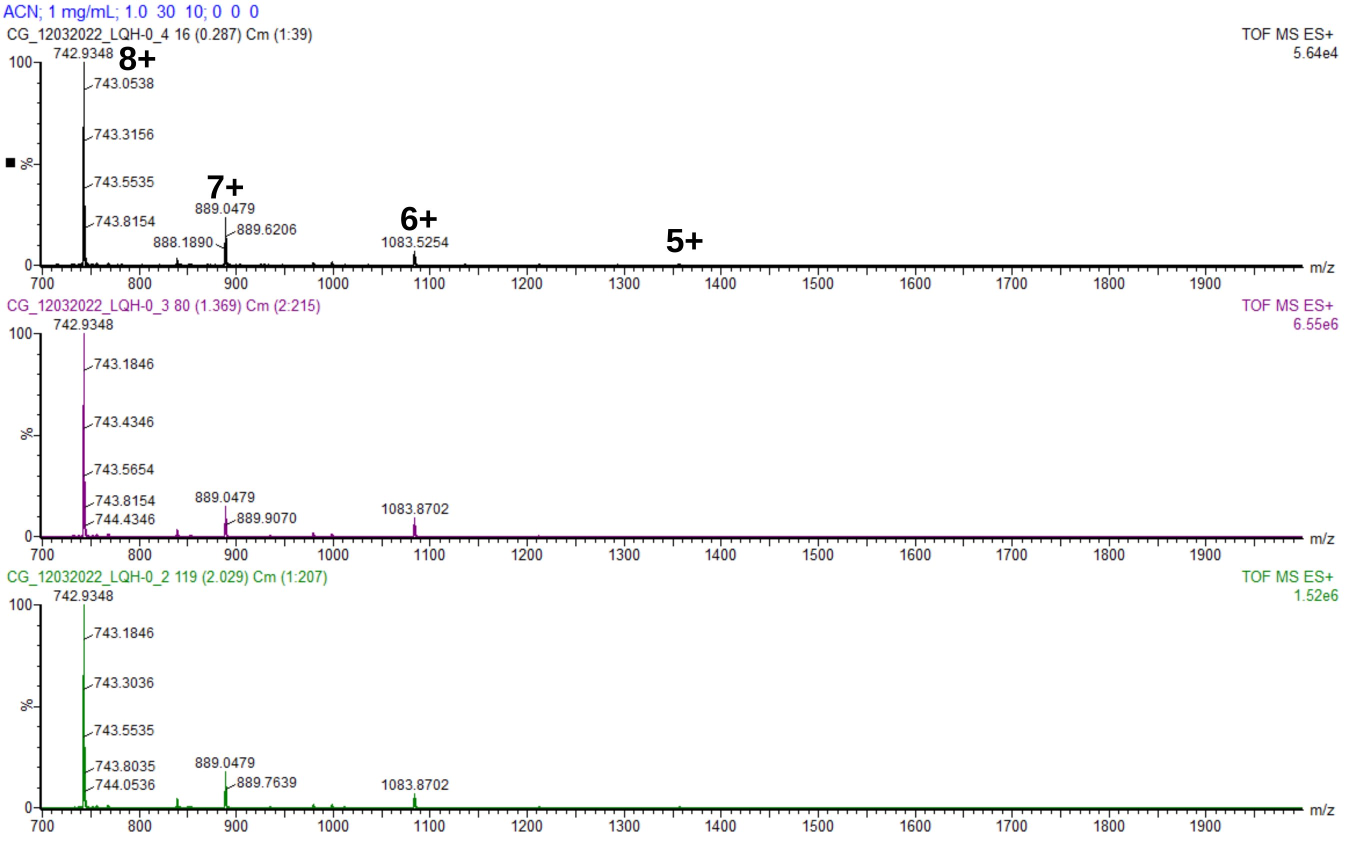

Figure S47. ESI-MS spectrum of **C1.**

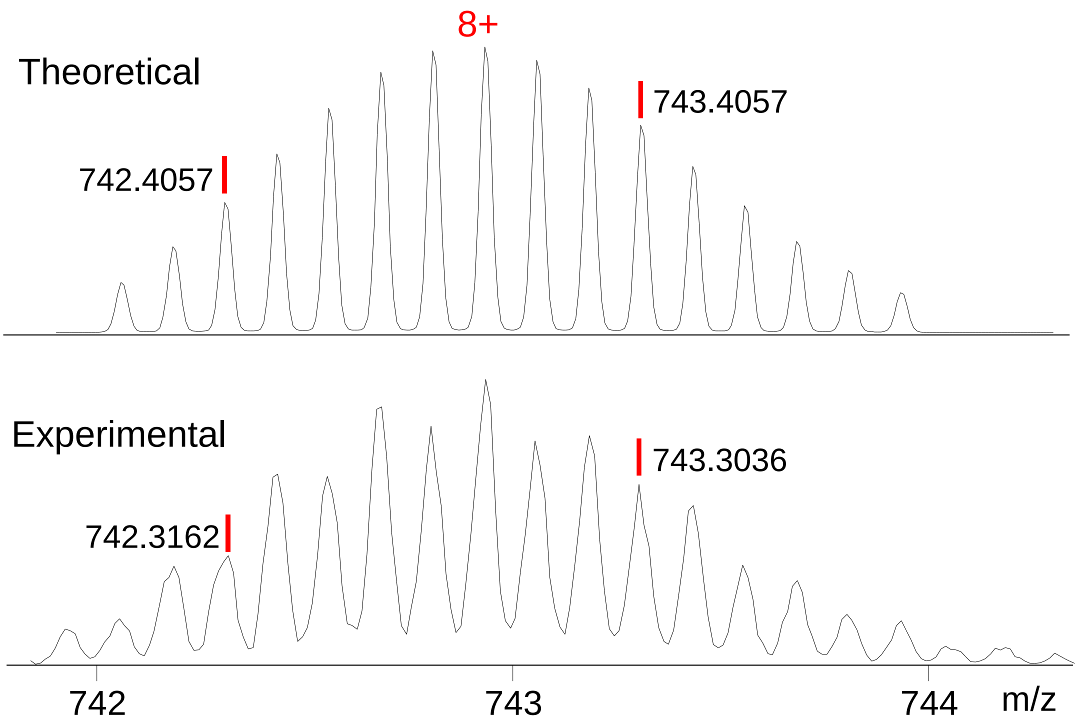

Figure S48. ESI-MS spectrometry analysis of **C1** showing the +8 peak.

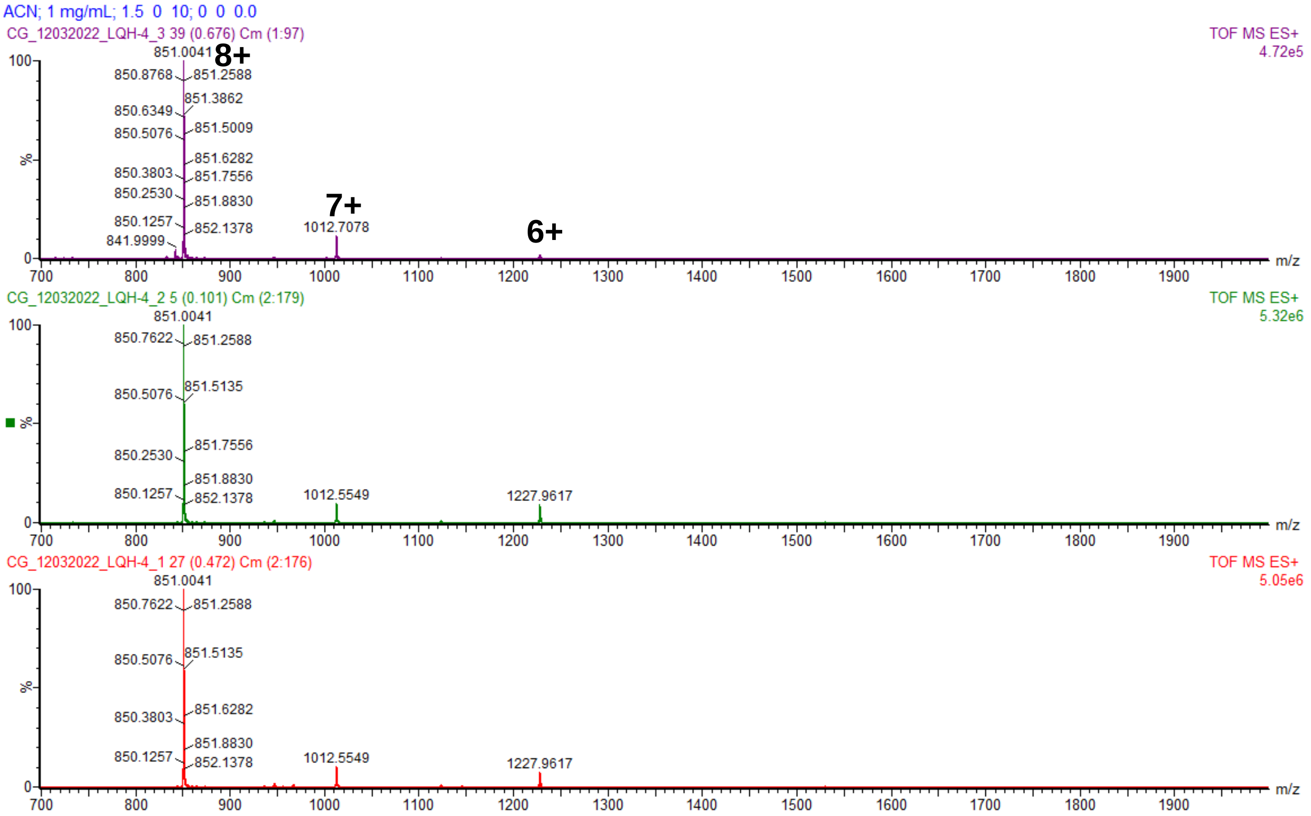

Figure S49. ESI-MS spectrum of **C2.**

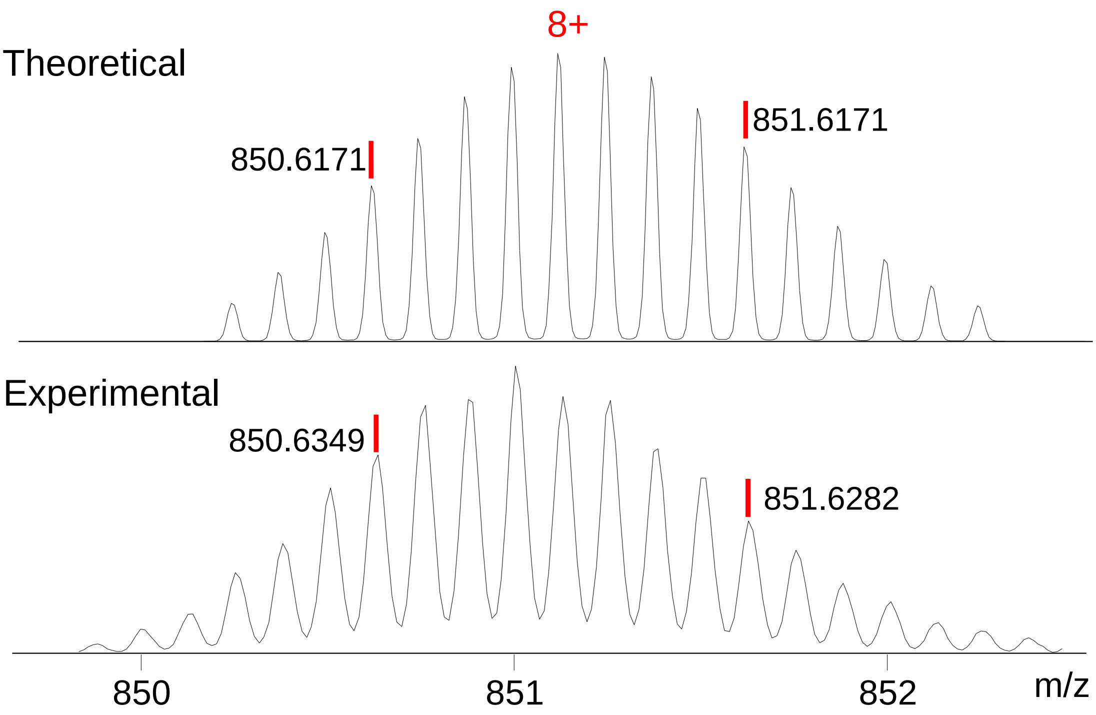

Figure S50. ESI-MS spectrometry analysis of **C2** showing the +8 peak.

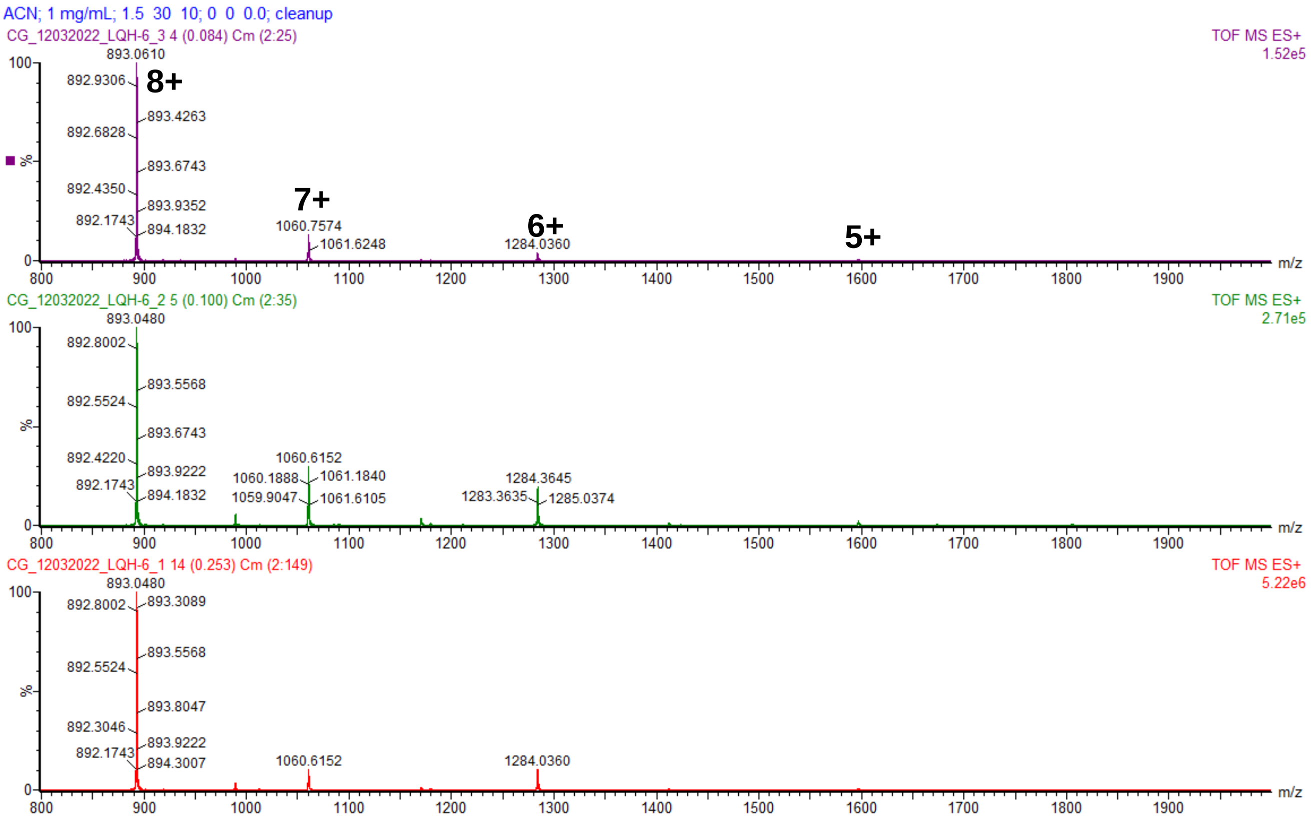

Figure S51. ESI-MS spectrum of **C3.**

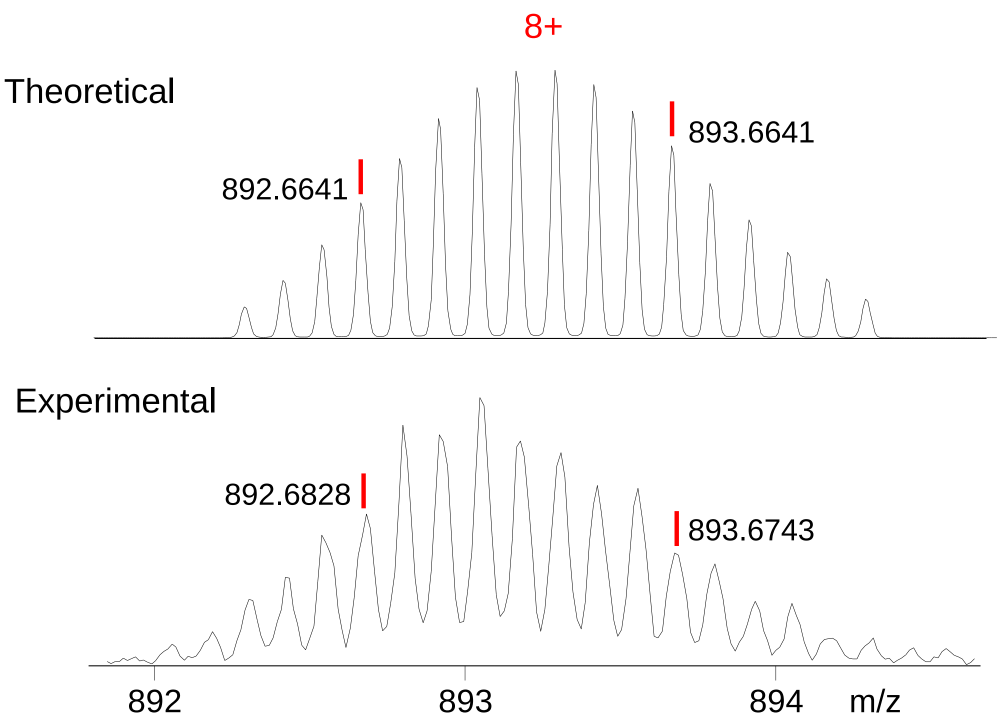

Figure S52. ESI-MS spectrometry analysis of **C3** showing the +8 peak.

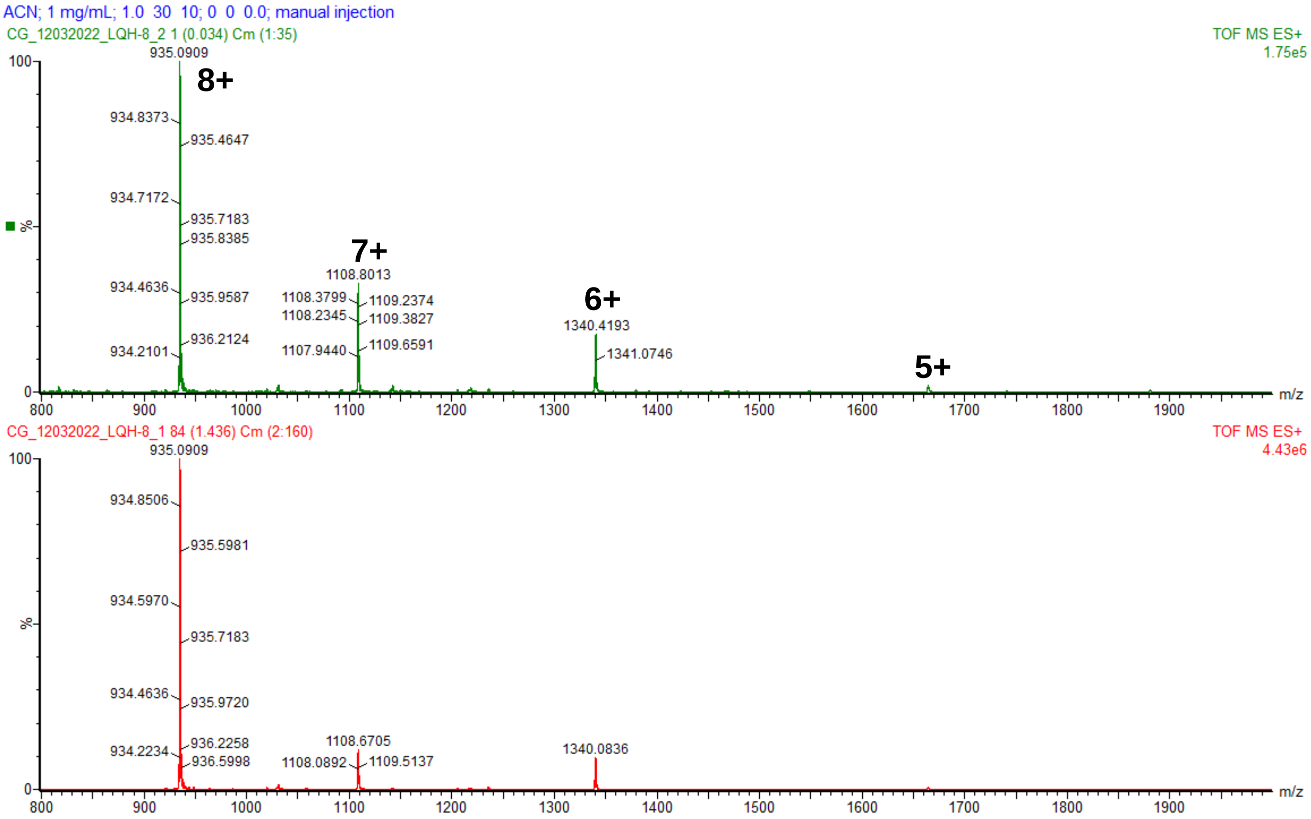

Figure S53. ESI-MS spectrum of **C4.**

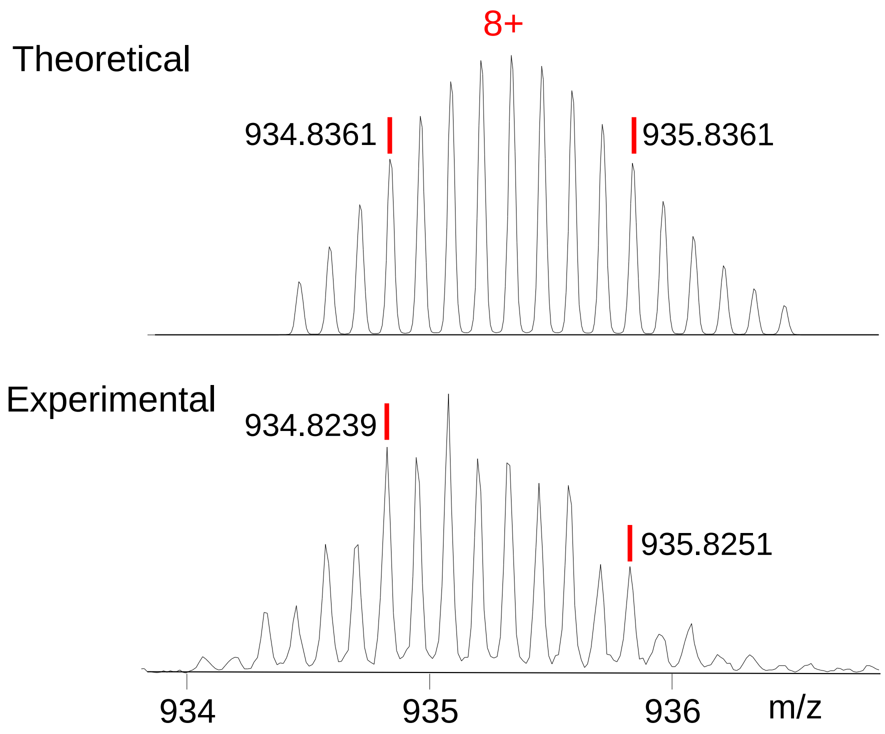

Figure S54. ESI-MS spectrometry analysis of **C4** showing the +8 peak.

a)

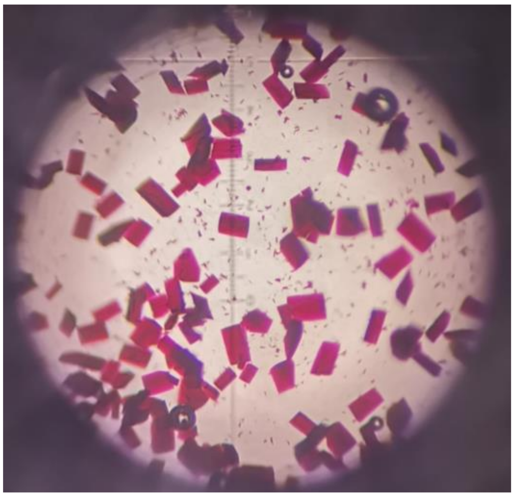

b)

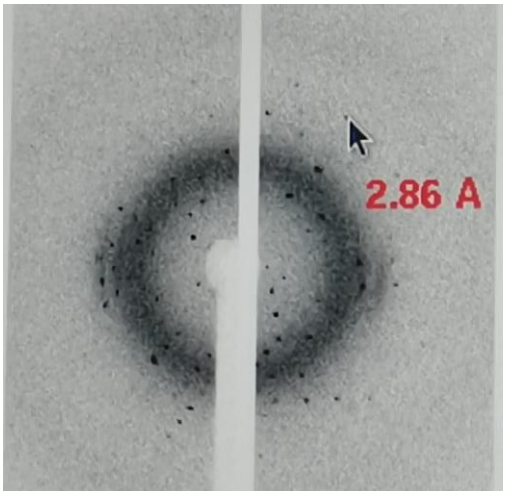

Figure S55. a) Micrograph and b) X-ray diffraction result of **C2'** single crystal.

Table S1. Crystal data and structure refinement for subcomponent **L1**.

Identification code	exp_2956
CCDC number	2255701
Empirical formula	$C_{25}H_{24}N_2O_2$
Formula weight	384.46
Temperature/K	294.7(2)
Crystal system	monoclinic
Space group	I2/a
a/Å	11.6759(10)
b/Å	16.7196(16)
c/Å	26.2350(16)
α/°	90
β/°	97.161(7)
γ/°	90
Volume/Å^3	5081.6(7)
Z	8
ρ_{calc}g/cm^3	1.005
μ/mm^{-1}	0.506
F(000)	1632.0
Crystal size/mm^3	0.36 × 0.22 × 0.18
Radiation	CuKα (λ = 1.54184)
2Θ range for data collection/°	6.282 to 134.15
Index ranges	$-13 \le h \le 13, -19 \le k \le 19, -31 \le l \le 31$
Reflections collected	51369
Independent reflections	4516 [R_{int} = 0.1288, R_{sigma} = 0.0732]
Data/restraints/parameters	4516/4/267
Goodness-of-fit on F^2	1.055
Final R indexes [I>=2σ (I)]	R_1 = 0.0796, wR_2 = 0.2114
Final R indexes [all data]	R_1 = 0.1322, wR_2 = 0.2408

14. References

[1] A. Mayr, M. Srisailas, Q. Zhao, Y. Gao, H. Hsieh, M. Hoshmand-Kochi, N. St. Fleur, *Tetrahedron* **2007**, *63*, 8206.

[2] W. Si, Z.-T. Li, J.-L. Hou, *Angew. Chem.* **2014**, *126*, 4666.

[3] a) N. Sakai, Y. Kamikawa, M. Nishii, T. Matsuoka, T. Kato, S. Matile, *J. Am. Chem. Soc.* **2006**, *128*, 2218; b) R. Kawano, N. Horike, Y. Hijikata, M. Kondo, A. Carné-Sánchez, P. Larpent, S. Ikemura, T. Osaki, K. Kamiya, S. Kitagawa, S. Takeuchi, S. Furukawa, *Chem* **2017**, *2*, 393.

[4] M. J. Abraham, T. Murtola, R. Schulz, S. Páll, J. C. Smith, B. Hess, E. Lindahl, *SoftwareX*, **2015**, *1*, 19.

[5] K. G. Sprenger, V. W. Jaeger, J. Pfaendtner, *J. Phys. Chem. B.* **2015**, *119*, 5882.

[6] T. Lu, F. Chen, *J. Comput. Chem.* **2012**, *33*, 580.

[7] D. J. Evans, B. L. Holian, *J. Chem. Phys.* **1985**, *83*, 4069.

[8] M. Parrinello, A. Rahman, *J. Appl. Phys.* **1981**, *52*, 7182.

[9] T. Darden, D. York, L. Pedersen, *J. Chem. Phys.* **1993**, *98*, 10089.

[10] H. J. C. Berendsen, J. P. M. Postma, W. F. Van Gunsteren, A. DiNola, J. R. Haak, *J. Chem. Phys.* **1984**, *81*, 3684.

[11] X. Sun, O. Klingbeil, B. Lu, C. Wu, C. Ballon, M. Ouyang, X. S. Wu, Y. Jin, Y. Hwangbo, Y.-H. Huang, T. D. D. Somerville, K. Chang, J. Park, T. Chung, S. K. Lyons, J. Shi, H. Vogel, M. Schulder, C. R. Vakoc, A. A. Mills, *Nature* **2023**, *613*, 195.
